# The impact of public policy on socioeconomic equity in physical activity: a systematic review

**DOI:** 10.1186/s12966-026-01880-6

**Published:** 2026-02-04

**Authors:** Fleur Heuvelman, Leonie Birkholz, Antonina Tcymbal, Jeroen Lakerveld, Joline W. J. Beulens, Catherine Woods, Karim Abu-Omar, Kevin Volf, Petru Sandu, Rasa Jankauskiene, Anna Gobis, Joanna Wachnicka, Joanna Żukowska, Peter Gelius, Linda J. Schoonmade, Sven Messing, Nicolette R. den Braver

**Affiliations:** 1 Department of Epidemiology and Data Science, Amsterdam University Medical Center, Meibergdreef 9, Amsterdam, The Netherlands; 2 Department of Sport Science and Sport, Friedrich-Alexander-Universität Erlangen-Nürnberg (FAU), Erlangen, Germany; 3 Physical Activity for Health Research Centre, Department of Physical Education and Sport Sciences, University of Limerick, Limerick, Ireland; 4 Department of Public Health, University Babes-Bolyai, Cluj-Napoca-Napoca, Romania; 5 Health Research and Innovation Science Centre, Klaipeda University, 92294 Klaipėda, Lithuania; 6 Faculty of Civil and Environmental Engineering, Gdansk University of Technology, Gabriela Narutowicza 11/12, 80-233 Gdansk, Poland; 7 Medical Library, Vrije Universiteit Amsterdam, De Boelelaan 1117, P.O. Box 7057, 1007 MB Amsterdam, The Netherlands; 8 Upstream Team, Amsterdam, Netherlands; 9 Amsterdam Public Health, Health Behaviours and Chronic Diseases, Amsterdam, the Netherlands; 10 Université de Lausanne, Institut Des Sciences du Sport, Lausanne, Switzerland

**Keywords:** Policy, Review, Physical activity, Equity

## Abstract

**Background:**

Increasing population-level physical activity (PA) requires system-level policy action. However, public policies targeting the general population, without addressing socially disadvantaged populations, might unintentionally increase socioeconomic inequities in PA. This is particularly concerning since disadvantaged groups are less likely to meet PA recommendations to begin with. This systematic review assesses evidence on the effects of public policies on equity in PA.

**Methods:**

A literature search was performed in seven bibliographic databases on May 7, 2024, in collaboration with a librarian. Studies were included if they a) focused on changes in PA behaviour, PA proxies, or the PA environment as outcomes, b) examined public policy as the independent variable, and c) included a low socioeconomic status (SES) (sub)population. Screening was done in duplicate. Key data extracted included: public policy information, target population and/or SES subgroup measures, PA outcomes, and equity-related findings. Policies were grouped into domains aligned with the eight investments of the International Society for Physical Activity and Health and categorized based on their impact on inequities: reduction, increase, no effect, or mixed effects.

**Results:**

Out of 10,350 records screened, 81 studies were included. Results showed that 27% of the public policies reduced inequities, 38% had no effect, 10% increased them, and 25% had mixed effects. The fewest PA policies were identified in the healthcare (*n* = 2) and workplace (*n* = 0) domains, the most in the community-wide domain (*n* = 22). Based on the available evidence, the school, transport, community-wide, and mass media policy domains most frequently demonstrated potential to reduce inequities in PA and/or to benefit high and low SES populations equally. Policies that most consistently reduced inequities or had a neutral equity effect included a) infrastructure policies, b) financial incentives supporting active transport, c) multi-component school-based PA and health policy programmes, d) school physical education policies, and e) policies supporting mass media campaigns. Conversely, urban design and sport for all policies varied in their effects on inequities.

**Conclusions:**

Most policies do not appear to exacerbate inequities. Policies in the school, transport, community-wide, and mass media domains show particular promise for promoting PA in an equitable way. These findings offer valuable insights for future policymaking.

**Supplementary Information:**

The online version contains supplementary material available at 10.1186/s12966-026-01880-6.

## Background

Physical activity (PA) has numerous well-known health benefits, including a reduction in the risk of non-communicable diseases such as type 2 diabetes and cardiovascular diseases [1–3]. It can, furthermore, enhance mental health and wellbeing, and bone health [4–9]. Despite these advantages, nearly one-third (31%) of adults worldwide did not meet the recommended levels of PA in 2022 [10], marking a concerning negative trend, as 23% of adults failed to meet these in 2000 [10]. If this trend continues, the World Health Organization (WHO) global target of achieving a 15% relative reduction in physical inactivity between 2010 and 2030 will likely remain unmet [2].

With global PA levels declining, population groups that are less active – such as those from lower socioeconomic backgrounds – may fall even further behind. Evidence, primarily from high-income countries, show that adults with higher socioeconomic status (SES) are more likely to participate in leisure-time PA compared to those with lower SES [11]. Conversely, those with lower SES tend to be more active in transport-related PA [11, 12]. Studies on occupational PA report inconsistent health outcomes [13, 14]. While some indicate beneficial effects, others show no effect or even adverse outcomes. Similar disparities exist among children and adolescents, with research indicating that those from lower SES backgrounds face an increased risk of low PA [15]. These differences may partly result from inequitable access to PA-supportive environments, such as urban green spaces and sport facilities [16–18]. Additionally, PA programmes might fail to address the needs of those groups, either by overlooking structural barriers (e.g., unavailability of resources, issues of literacy) or by not engaging these communities in programme development and implementation [19, 20]. Addressing these inequities in PA promotion is crucial, as PA plays a role in reducing broader health disparities [21, 22].

In the Global Action Plan for Physical Activity (GAPPA), the WHO defines health inequity as an avoidable, unfair, and unjust difference between groups of people [23]. It is also stated that inequities arise from circumstances linked to SES, living conditions and other social, geographical, and environmental determinants that can be improved through targeted interventions. GAPPA underscores that disparities in PA participation are evident across various groups, such as those defined by SES, age, gender, and disability, reflecting inequities in access to PA opportunities. In this study we focus specifically on inequities driven by SES disparities, as there is still a gap in our understanding of how to promote PA in an equitable way among socioeconomically disadvantaged groups [24–26]. Given the health disparities faced by low SES individuals [27, 28], identifying effective strategies to promote PA in an equitable way is imperative.

PA happens within broader environments that can either encourage or limit PA. Public policies play a crucial role in regulating and changing these environments and are thus important upstream determinants of population PA levels [29–32]. Examples include laws mandating participation in regular physical education (PE) lessons in schools, or infrastructural regulations that may promote walking and cycling [33]. The relevance of policy-driven approaches to improve PA is increasingly recognized across multiple sectors, including health, transport, schools, and the workplace [34–36]. Consequently, whole system approaches that leverage and integrate these sectors have been recommended to increase PA levels [23, 34, 37, 38], mirroring approaches recommended for enhancing population health by reducing tobacco use and promoting healthy food environments [39, 40]. Evidence from the tobacco and food sector suggests that public policies can positively impact equity [41, 42].

Public policies have the potential to create equitable opportunities for PA participation and reduce inequities in PA. However, despite their potential impact, reaching equity is often not a focus in PA policies [43]. This is concerning because population-based PA policies that do not account for existing disparities, might inadvertently increase health inequities by primarily reaching higher SES groups [44]. For example, non-refundable fitness tax credits intended to reduce the cost of participating in organized PA (including sport) primarily benefit families who owe a certain amount of income tax [45]. Because these credits can only reduce taxes owed, low-income families—who pay little or no income tax—receive no benefit from these tax credits, making this policy ineffective in helping those most in need of financial support. Ideally, equitable PA policies should consider the multiple barriers faced by disadvantaged populations such as limited access to information, financial constraints, environmental challenges, low health and physical literacy, and also address cultural considerations [20]. This underscores the need to examine the equity effects of PA-related policies to understand which policies can reduce or, by contrast increase socioeconomic inequities in PA. While such assessments exist for other areas of public health [46], there is, to our best of knowledge, so far no overview for the field of PA. Therefore, our aim was to assess the current evidence on the effects of public policies on equity in PA across different domains.

This review was conducted as part of the IMproving Physical Activity policies and their impact on health eQuiTy (IMPAQT) project [47], which aims to improve health equity in and through physical activity. Within IMPAQT, policy benchmarking is developed, tested, and promoted as a key tool to support this objective. The findings of this review inform the adaptation of the Physical Activity Environment Policy Index (PA-EPI) [48] to better incorporate an equity perspective.

## Methods

This study is a systematic literature review. The Preferred Reporting Items for Systematic Reviews and Meta-Analyses extension (PRISMA) 2020 was used as the foundation (see Additional file 1 for the completed checklist) [49]. The review protocol was registered on PROSPERO (ID: CRD42024569261) on July 15, 2024.

### Search strategy

In collaboration with a medical librarian (LS), a comprehensive search was performed on May 7, 2024 in seven bibliographic databases: PubMed, Embase, CINAHL Plus, Scopus, Web of Science Core Collection, SportDiscus and the International Bibliography of the Social Sciences (IBSS). Search terms included controlled terms (MeSH in Medline and Emtree in Embase) as well as free-text terms. The following terms were used (including synonyms and closely related words) as index terms or free-text words: ‘physical activity' and ‘equity' and 'policy' and 'impact'. The search was performed without date or language restrictions. References with 'trial' in title were excluded. Duplicate articles were excluded by a medical information specialist (LS) using DedupEndNote following a manual deduplication in EndNote X20.5 (Clarivate^tm^) [50]. The full search strategies for all databases can be found in the Supplementary material (See Additional file 2). Additional studies were identified by manually searching reference lists of included primary studies and systematic reviews.

### Study inclusion criteria

#### Study design

Only published peer-reviewed articles were included (i.e., no policy reports or conference abstracts). We included studies that allowed for the identification of policy effects, i.e., studies that collected data before and after policy implementation, as well as post-policy studies featuring a population not exposed to the policy (post-test design with control group). Additionally, studies that retrospectively assessed changes were included, such as those asking if PA behaviour changed following the policy implementation. Qualitative evidence was incorporated alongside quantitative data.

#### Exposures: policy

Studies were considered eligible for inclusion if they evaluated the direct or indirect impact of public policies- on PA behaviour and/or the PA environment, defined as the physical and social environment (e.g., facilities, equipment, action plans, programmes) hypothesized to lead to changes in PA outcomes as a result of a policy. Furthermore, they had to target either the general population or a specific socioeconomically disadvantaged subgroup. This meant that a subgroup of or the entire study population had to be socioeconomically disadvantaged, defined by income, education, occupation, area and/or setting-level disadvantage. Eligible studies had to examine differential effects across groups facing and not facing inequities (e.g., PA effects of a population-based policy in a low-income versus a high-income population) or the ‘overall’ effect of a (targeted) policy in a socioeconomically disadvantaged group (e.g., effect of a policy on PA in a population living in low-income areas).

We used the following definition of public policies: “purposeful decisions, plans or actions made by voluntary or authoritative actors in a system designed to create system-level change to directly or indirectly achieve specific societal goals. Within this definition, public policy is a form of government action usually expressed in a law, a regulation, or an order. Since it reflects an intent of government or its representative entities.” [51]. Both policies and research interventions were included, provided there was evidence of government action. To enhance readability and clarity, the term ‘policy’ is used broadly to encompass any form of government action, including research-driven interventions, actions or programmes. Under this broad definition, initiatives at for example the school or workplace level were considered policy if the government acted as an initiator, partner or provided funding. Studies assessing the effectiveness of initiatives or interventions presented as policies but lacking such evidence were excluded (e.g., organizational policies implemented independently of government). When necessary, government websites were consulted to verify whether an intervention or programme met our public policy criterion. Policies did not have to be enacted for health-related reasons to be included, and could have been implemented at local, regional or national levels. As a result, policies such as transport initiatives aimed at promoting environmental sustainability were also considered. While changes of built environments are often driven by policy, studies assessing the impact of such changes on PA were excluded unless they were explicitly connected to the implementation of a specific policy.

#### Outcomes

Studies were eligible for inclusion if they analysed PA as an outcome, i.e. changes in PA (e.g. walking, cycling, and meeting PA guidelines) or a proxy (e.g. physical fitness), assessed by means of self-report or wearable devices (e.g., accelerometer) or a change in features of the physical and social environment (e.g., facilities, equipment, action plans, programmes) hypothesized to lead to changes in PA outcomes as a result of a policy [52].

#### Participants

No age restrictions were applied to the study populations. We excluded studies targeted at clinical populations (i.e., specific patient groups). A subset of the study participants, or the entire study population, comprised individuals from lower SES backgrounds, who may face socioeconomic inequities. Therefore, studies were required to address inequity – explicitly or implicitly – within their scope.

### Study selection

All potentially relevant titles and abstracts were screened for eligibility twice, by two independent members of the research team. The web-based systematic review tool Covidence was used for facilitating the screening [53]. Differences in judgement were resolved by a third researcher. Subsequently, the same method was applied to full-text screening. When needed, disagreements were resolved through consensus discussion. Umbrella reviews were excluded during full-text screening but were used for manual reference searching. Relevant primary studies were identified from these initially excluded reviews and subsequently included.

### Data extraction

For each study, data were extracted by one researcher using a piloted extraction form. A random subsample of 10% was extracted in duplicate to assess the level of inconsistencies, which were resolved through discussion to reach agreement. The data extraction template included details related to policy, measure(s) of inequity, PA-related outcome(s), overall main finding(s) (if differential effects by SES were available; otherwise, this was not applicable) and finding(s) related to equity. Policy information also entailed the related policy domain. Domains were based on the eight investments of the International Society for Physical Activity and Health (ISPAH), i.e., whole-of-school programmes, active transport, active urban design, healthcare, public education, sport and recreation for all, workplaces, and community-wide programmes [35]. For the criteria used to cluster policies within domains, we refer to Additional file 3. If a policy was challenging to categorize, the core research team (LB, SM, NRdB, FH) collaboratively decided the most appropriate domain to assign it to. If it was not possible to categorize a policy based on the eight investments, a new ‘domain’ was created. To assess equity effects, we aimed to obtain studies that reported differential effects by measures of socioeconomic inequity. These studies were considered to provide strong evidence because they allowed for direct comparisons between SES groups. Where these cross SES results were unavailable, but there were indications of inequities affecting the broader study population, we extracted overall PA-related outcomes for disadvantaged populations (e.g., area or community level). This included studies where policies specifically targeted low SES groups or were implemented exclusively in economically deprived areas. However, since these studies lacked a high SES comparison group, they were considered to provide weaker evidence for assessing equity effects. If a primary study reported multiple PA outcomes, subgroup outcomes (based on measures of socioeconomic disadvantage) or policies, we extracted all of them. Results of the underlying relevant primary studies within reviews were included and extracted using a similar extraction sheet as for the primary studies. This means that extraction was performed at the highest possible level of detail, that is, by extracting reported results per primary paper and not the overall conclusions of the reviews, in order to outline data extraction of the primary studies. Results underpinning the assessment of impacts on inequities were verified for all primary studies. Other extracted information (e.g., policy characteristics) was cross-checked only when unclear from the review reports.

For included review studies, reference lists were checked for duplicates against the included primary studies to avoid double counting. Primary studies were excluded from data extraction, and only the (validated) detailed results from the reviews were included. If multiple reviews contained overlapping primary studies, we prioritized the review that aligned best with our extraction methods for primary studies. For example, if one review reported only a single relevant outcome from a primary study while another provided multiple, the latter was included and analysed.

### Risk of bias

Risk of bias (RoB) was assessed independently by two members of the research team. Discrepancies in scoring were resolved by two core team members (NRdB, FH). We applied a slightly adapted version of the RoB method used by Ogilvie et al. 2007 [54], as further applied and informed by Ding et al. (2024) [55], which uses five binary criteria to evaluate study quality across diverse study designs. This approach was deemed appropriate given the methodological heterogeneity of the studies included. The five criteria assess the presence of a control group, concurrent intervention/contamination, sample representativeness, group comparability, and either attrition or sample size. Detailed descriptions of these criteria are provided in Additional File 4.

For studies included via reviews, we incorporated the quality assessments of primary studies as reported in those reviews. We also documented the specific tools used in each review to provide contextual clarity (see Additional File 5).

### Data synthesis

The impact of a policy on inequities in PA was defined as follows [56]:

1. Reduced inequities: a) The most disadvantaged group (i.e., group experiencing inequities) responded more favourably to the policy compared to the least disadvantaged group (i.e., group not experiencing inequities) or b) The disadvantaged group responded favourably to the policy (i.e., when no advantaged comparison group was available).
2. Increased inequities: a) The most disadvantaged group responded less favourably to the policy compared to the least disadvantaged group or b) The disadvantaged group responded negatively to the policy.
3. No effect on inequities: a) The most disadvantaged and least disadvantaged groups responded similarly to the policy or b) The disadvantaged group did not respond to the policy.

Each extracted policy–outcome combination was categorized based on its impact on inequity. Assessments of the policies' effects on PA inequities were based on significant findings, e.g., significant interactions by SES measure/subgroup analyses or outcomes were significantly better or worse in a disadvantaged community after the policy implementation. If no significance test was available, assessment was based on the direction of the reported effect measures. A meta-analysis was not possible due to large heterogeneity in measurements, outcomes, and inclusion of qualitative data. Consequently, the effectiveness of these policies in addressing PA inequities was summarized narratively.

To synthesize the available evidence, an overall label was assigned to each policy based on the reported results, which meant that every policy was labelled as either reducing inequities, increasing inequities, having no impact on inequities or inconclusive/mixed impact on inequities. If a study examined multiple policies, each described policy was reported separately, resulting in multiple entries for that study. If different types of outcomes pointed towards opposing directions, the label was based on the most relevant outcome(s), i.e., the outcome(s) relating to PA behaviour/individual-level outcome instead of the physical environment that could potentially change PA. A policy was classified as having mixed effects when less than 70% — the clear majority — of the outcomes reported similar results. This classification (mixed effects) was applied not only when different outcome measures showed conflicting effects but also in cases where effects varied by SES indicator or population group (e.g., adults vs. children) or when there was a discrepancy between the overall effect on a socioeconomically disadvantaged population and the effects on PA within specific SES subgroups of that population. The reporting of results was grouped by policy domain.

## Results

**Fig. 1:**
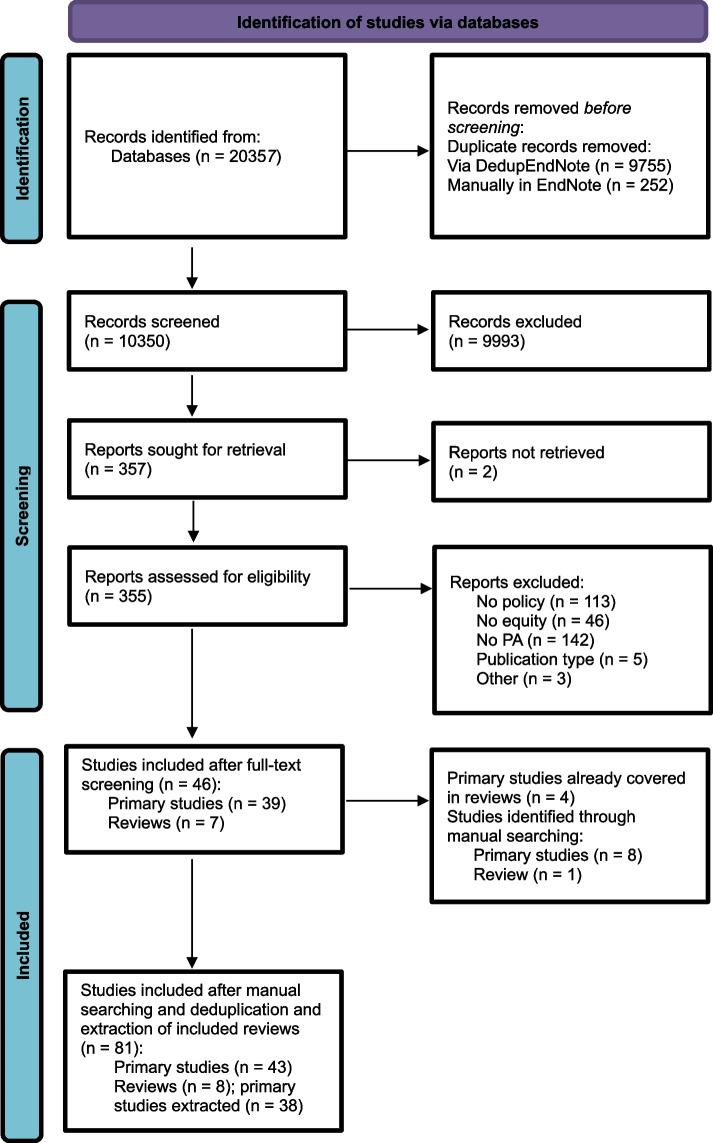
Adapted PRISMA Flow diagram for inclusion and exclusion of studies [49]

The literature search generated a total of 20,357 records (Fig. 1). After removing duplicates of records that were selected from more than one database, 10,350 records remained. After screening the titles and abstracts of these and reviewing the selected 355 full-text studies, there were 46 studies (39 primary studies and seven reviews) eligible for analysis. After excluding four primary studies (as they were already covered in an included review), nine additional studies were included through manual search resulting in a total of 51 studies (43 primary studies and eight reviews). As the eight reviews covered 38 additional primary studies, this review included the results of a total of 81 primary studies.

### Impact of policies within PA domains

Table 1 highlights the number of policy–outcome combinations for both cross SES results (*N* = 46) and low SES population (*N* = 51) specific results. Figure 3 illustrates the findings from cross-SES comparisons, while Fig. 4 focuses on outcomes within low SES populations. This distinction provides an indication of the strength of the evidence, as studies that report differential effects by SES can show whether the PA gap between high and low SES groups is narrowing. By contrast, studies focusing solely on low SES populations cannot provide this insight. The synthesized evidence yielded 85 overall impact ratings across the 81 studies. The number of ratings slightly exceeds the 81 primary studies because some of these studies reported relevant results for more than one policy [57–59]. Across the domains, 23 ratings showed reductions in inequities, 32 indicated no differences, nine showed increases, and 21 showed mixed effects. Most of the ratings fell in the domains community (*n* = 22), transport (*n* = 13), urban design (*n* = 14), schools (*n* = 10), sport and recreation for all (*n* = 10), and mass media (*n* = 8). Healthcare only had two ratings, and workplace had zero ratings. Policies were identified for two new domains, which were the ‘childcare’ (*n* = 4) and ‘social’ (*n* = 2) domain. Additional file 5 provides a summary of the PA outcomes of the policies across different SES subgroups, as well as within low SES populations, categorized by policy domain. The complete extraction sheet can be found in Additional file 6.

**Table 1:** The number of policy–outcome combinations for cross SES results and low SES population specific results

Policy domain	SES perspective	N studies^a^
Community-wide	Across SES	11
	Low SES	19
Transport	Across SES	10
	Low SES	3
Urban design	Across SES	5
	Low SES	11
Schools	Across SES	3
	Low SES	7
Sport for all	Across SES	6
	Low SES	6
Mass media	Across SES	8
	Low SES	0
Healthcare	Across SES	0
	Low SES	2
Childcare	Across SES	1
	Low SES	3
Social	Across SES	2
	Low SES	0
Total	Across SES	46
	Low SES	51

^a^Note that some studies reported both ‘cross SES’ and ‘low SES’ results

### Risk of bias

**Fig. 2:**
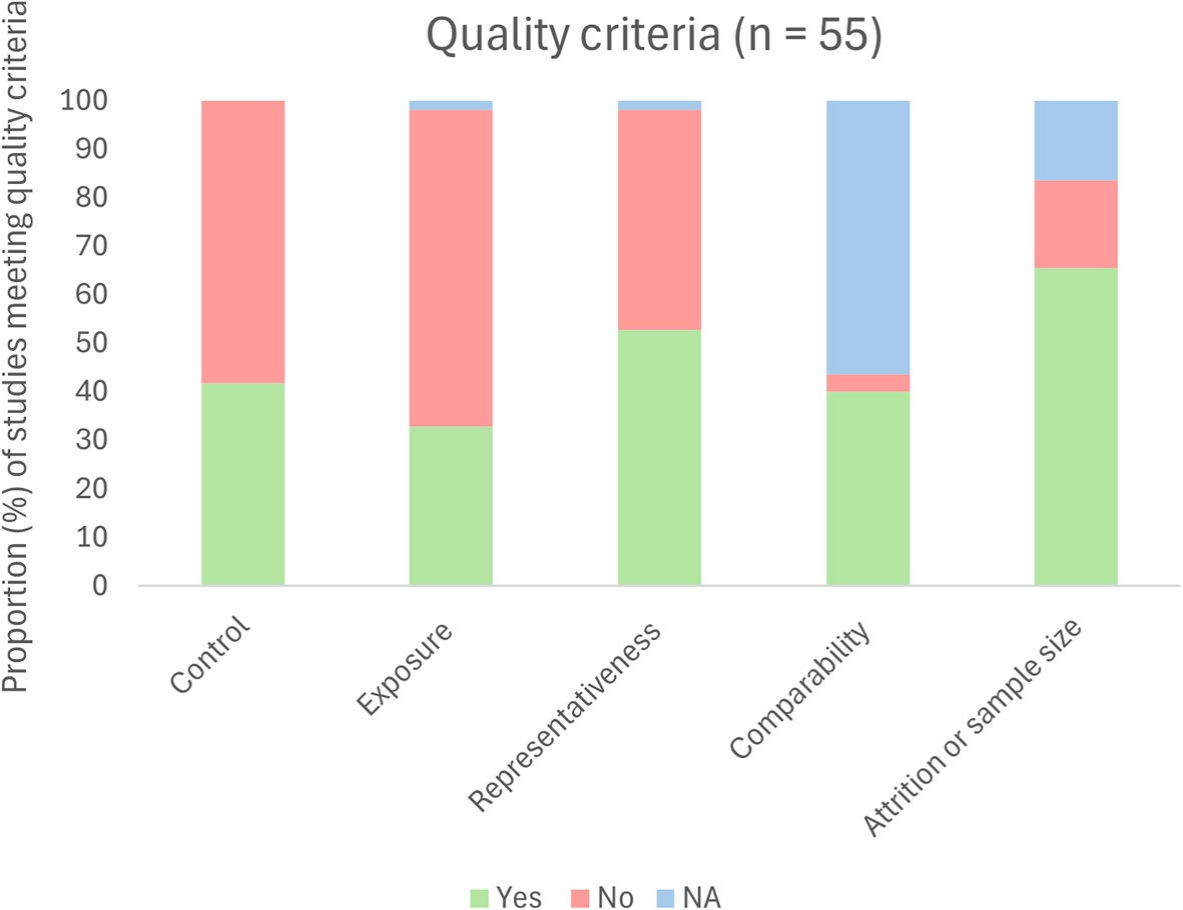
Proportion of studies meeting quality criteria

For 55 studies, we assessed the risk of bias (RoB). Among these studies, 17 (31%) met four or five criteria, seven (13%) met three, 10 (18%) met two, 13 (24%) met one, and eight (15%) met none. The attrition and sample size criteria were most frequently met, whereas the exposure criteria were least frequently met (Fig. 2). Only five out of 55 studies were randomized controlled trials. Based on the mean domain scores, studies in the school and community domains scored highest (3.7 and 3.0, respectively), while sport scored lowest (1.1). The full RoB assessments for all 55 studies are provided in Additional file 4.

Of the studies evaluated in the review by Hunter et al. (2019; *n* = 9) using the Twohig-Bennett criteria [60], five (56%) were classified as high quality. Other primary studies from reviews (*n* = 17) were assessed using a similar scoring method [56, 61–63]. Among these, 4 (23%) were rated as strong, 10 (59%) as moderate, and 3 (18%) as weak in quality.

### Community-wide

#### Overall

Of the 22 studies examining policies within this domain, three reported reductions in inequities, seven found no differences, and one observed an increase in inequities. Three studies reported mixed effects. Eight studies provided both cross SES and low SES results, with these results being largely consistent (Additional file 5) [62, 64, 65].

#### Across SES

**Fig. 3:**
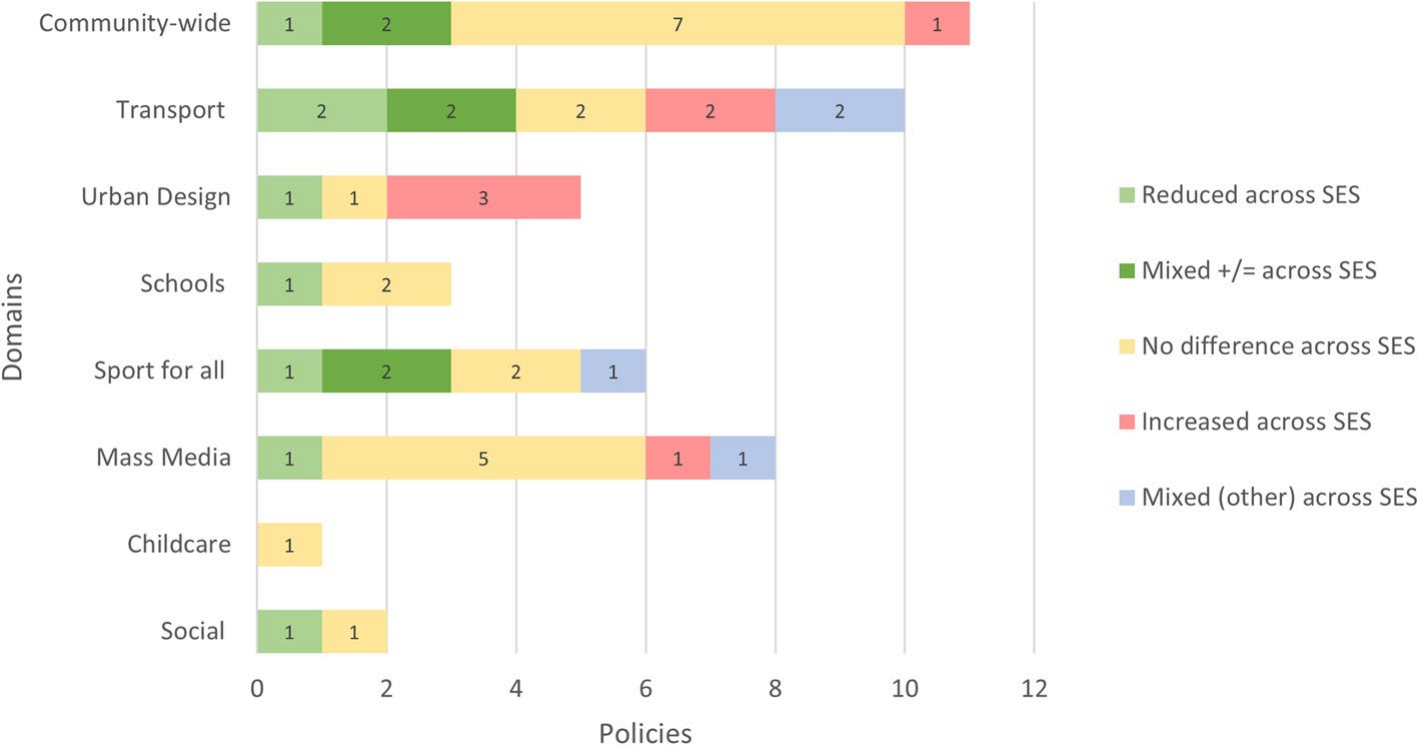
Evidence on policy and inequities in PA by domain: ‘across SES’ results. Mixed (+/=) indicates studies that showed both a positive and a neutral effect. Mixed (other) indicates studies that showed either a neutral and negative effect or both positive and negative effects

Among the eleven studies that reported differential effects by SES, one (9%) found reductions in inequities, two found both reductions and no differences (18%), seven (64%) found no differences, and one (9%) observed increases in inequities (Fig. 3). Policies associated with reduced or unchanged inequities were diverse, including free access to activities at leisure centres with community outreach, a mix of cycling promotion initiatives, and comprehensive land use planning [56, 62, 64, 66, 67]. Increased inequities were observed following a PA community programme [65].

#### Low SES

**Fig. 4:**
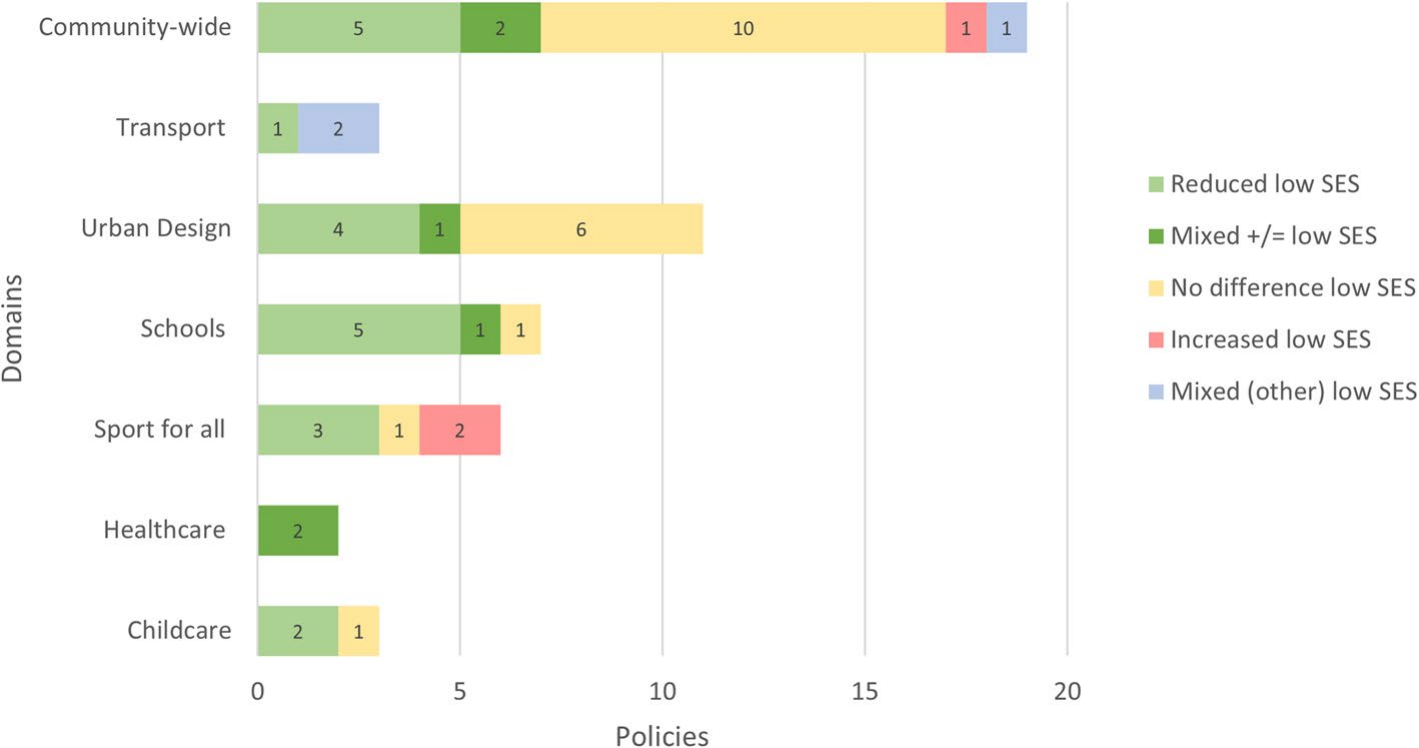
Evidence on policy and inequities in PA by domain: ‘low SES’ results. Mixed (+/=) indicates studies that showed both a positive and a neutral effect. Mixed (other) indicates studies that showed either a neutral and negative effect or both positive and negative effects

Among the 19 studies that focused on low SES populations, five (26%) reported positive impacts, two (11%) found both positive and no impacts, ten (53%) found no impact, one (5%) found a negative impact, and one (5%) reported mixed impacts (both positive and negative impacts) (Fig. 4). As with the cross SES results, the policies with positive impacts in low SES populations were diverse. Many community PA/health programmes had no impact [62, 64, 65, 68, 69]. Two studies on urban renewal programmes and one on health activities within such programmes showed no impact, though one urban renewal study reported a positive policy impact [62, 70–72]. For park renovations combined with park initiatives, contrasting results emerged, with one study reporting no impact and another showing both a positive and no policy effect [58, 60]. A negative impact was observed following one community health program [62]. Walking increased and vigorous PA decreased following an environmental intervention [73, 74].

### Transport

#### Overall

Of the twelve studies examining thirteen transport policies, three reported reductions in inequities, two found no differences, and two observed increases. Additionally, six studies showed mixed results. The one study examining two policies reported both reduced inequities and mixed effects.

#### Across SES

Among the ten studies analysing differential effects by SES, two (20%) reported reduced inequities, two (20%) reported both reduced inequities and no differences, two (20%) found no differences, two (20%) observed increased inequities, and two (20%) indicated mixed effects (Fig. 3). Policies linked to reduced or unchanged inequities in PA included infrastructure improvements, road pricing and a national free bus pass [56, 63, 75, 76]. By contrast, increased inequities were observed only for infrastructure-related policies [77, 78]. Mixed effects were seen following a regional transportation plan, which reduced inequities for transport-related walking but widened inequities in leisure walking [79]. Similarly, new transit infrastructure showed varied effects depending on gender and type of PA: reduced inequities (transport PA) and increased inequities (recreational PA) were seen in men, while in women reduced inequities were observed across all outcomes (transport and recreational PA) [63]. Overall, more favourable impacts were mainly observed on transport-related PA outcomes, rather than recreational PA.

#### Low SES

Among the two studies examining three policies focused on low SES populations, a positive impact was observed following the expansion of a bicycle sharing system (BSS) to deprived areas (33% of the policies had a positive impact) [59], while the other policies (67%) showed mixed impacts (Fig. 4). These included a BSS price increase, which showed both a negative and neutral impact on BSS usage among residents of deprived areas [59], and improvements on pedestrian route use, which yielded inconsistent results [80].

### Urban design

#### Overall

Of the fourteen studies examining urban design policies, three reported reductions in inequities, six observed no differences, and three observed increased inequities. Two studies provided both cross SES and low SES results, which were largely consistent [81].

#### Across SES

Among the five studies that examined differential effects by SES, one (20%) found reduced inequities following trail construction, one (20%) found no difference following changes in greenery, while three (60%) reported increased inequities after playground and park renovations and a housing policy (Fig. 3) [60, 81–83].

#### Low SES

Among the eleven studies focusing on low SES populations, four (36%) reported reduced inequities, one (9%) reported reduced inequities and no difference, and six (55%) found no difference (Fig. 4). Positive policy impacts were reported following park renovations and trail construction, while a housing policy showed no beneficial impact [60, 81, 84, 85]. However, most studies on park renovations found no policy effect [60, 61]. Additionally, greenery interventions either had a positive or no impact in adolescents, or no impact in adults [60].

### Schools

#### Overall

Of the ten studies examining school policies, six reported reductions in inequities, three found no differences, none observed increased inequities, and one reported mixed results.

#### Across SES

Among the three studies reporting differential effects by SES, one (33%) found reduced inequities, while two (67%) reported no difference in inequities related to school PE policies and broader state-level policies and programmes (Fig. 3) [56, 86].

#### Low SES

Among the seven studies focusing on low SES populations, five (72%) reported reduced inequities, one (14%) found both reduced inequities and no difference, and one (14%) found no difference (Fig. 4). Positive policy impacts on PA were reported following multi-component health or PA-promoting school policies, teacher or facilitator training to promote PA in schools, and a Walking School Bus programme [58, 87–91]. However, one of the two studies on teacher training also showed no impact on PA [91], and one study found that playground renovations did not impact PA [61].

### Sport and recreation for all

#### Overall

Of the nine studies examining ten sports for all policies, three reported reductions in inequities, two found no differences, and two observed increases. Additionally, three policies across two studies showed mixed results. Two studies provided both cross SES and low SES results, which were largely consistent (Additional file 5) [92, 93].

#### Across SES

Among the five studies analysing differential effects by SES, six policy–outcome combination were identified. Of these, one (17%) found reduced inequities, two (33%) observed both reduced inequities and no difference, two (33%) reported no differences and one (17%) found mixed effects (no difference and increased inequities) (Fig. 3). Policies associated with reduced or unchanged inequities included financial incentives, higher municipal sport expenditure, sport policy programmes (children), and indoor sport restrictions [57, 92–95]. However, both no impact on inequities and an increase in inequities were observed following sport policy programmes for adults [57].

#### Low SES

Among the six studies targeting low SES populations, three (50%) reported reduced inequities, one (17%) found no difference, and two (33%) observed increased inequities (Fig. 4). Consistent with the cross SES findings, positive impacts were seen following financial incentives [93, 96, 97]. However, a voucher scheme did not impact PA [92]. A decrease in PA facility use was observed following government subsidies for sport facilities and when a leisure centre shifted away from equitable service provision [98, 99].

### Mass media

#### Across SES

Of the eight studies on mass media campaigns, one (13%) reported a reduction in inequities, five (63%) observed no differences, one (13%) found an increase, and one (13%) reported mixed results (no difference and increased inequities) [100, 101]. All findings were based on differential effects by SES (Fig. 3). The mixed effect observed was due to a difference in how socioeconomic measures (e.g., income vs. education) influenced the outcome.

### Healthcare

#### Low SES

The two studies examining healthcare policies reported mixed impacts on inequities, with both positive and no impacts observed. Neither study reported differential effects by SES (Fig. 4). One study evaluated the replication of an existing health promotion program, while the other assessed the integration of a community resource guide used by clinic staff to connect families with local PA resources [61, 102].

### Childcare

#### Overall

Of the four studies examining childcare policies, two reported reductions in inequities, while the other two found no differences.

#### Across SES

One study that analysed differential effects by SES found no impact on inequities following obesity prevention policies (Fig. 3) [103].

#### Low SES

Among three studies targeting low SES populations, positive impacts were observed following the implementation of a health promotion programme for childcare services and initiatives aimed at supporting health-promoting changes at childcare sites (67% of policies had positive impact) (Fig. 4) [58, 104]. In contrast, no impact on PA was observed following the introduction of a guide for improving PA, alongside supporting resources (33% of policies had no impact) [105].

### Social

#### Across SES

One study examined a social assistance policy providing cash benefits [106]. The study found no differences in inequities following the policy (Fig. 3). Another study evaluated the Children’s Fitness Tax credit and reported reduced inequities in PA; however, the population reached by the policy was predominantly of higher socioeconomic status [56].

## Discussion

The aim of this systematic review was to provide an overview of the evidence on the impact of public policies on equity in PA. Overall, our review shows that a majority of the analysed policies did not exacerbate inequities in PA. The greatest amount of evidence was available for the community-wide domain and in low SES populations. Policies within the community-wide, transport, schools, and mass media domains generally showed reduced inequities or neutral effects on inequities in PA. By contrast, policies in the urban design and sport and recreation for all domains varied to a larger extent with regards to their effects on inequities. There was insufficient evidence to assess the impact of policies in the workplace and healthcare domains on inequity.

The following policy types appear to have the potential to reduce inequities and/or benefit both high and low SES groups equally: infrastructure policies and financial incentives that support active transport (e.g., road pricing and free bus passes), multi-component school-based PA and health policy programmes, school PE-related policies, and policies supporting mass media campaigns. However, for the community-wide domain, no specific equitable policy types could be clearly identified, suggesting that their effectiveness may be highly context-dependent. While evidence for the sport and recreation for all domain was more variable and the domain had the lowest mean quality score, findings indicate that financial incentives (e.g., free access to sports) may help reduce inequities.

### Impact on inequities in PA – results across settings

The majority of studies were found within the community-wide domain. Previous reviews have emphasized the importance of combining multiple policy components to achieve a substantial impact on health, which was central to the community-wide policies included in this review [107, 108]. Our review results indicated that community-wide policies generally had a neutral impact on, or reduced socioeconomic inequities. This suggests that these multi-component policies hold promise as more equitable strategies compared to policies targeting a single element. However, most studies were conducted in low SES populations rather than across different SES groups, indicating that they were primarily targeted at high-risk populations rather than implementing population-based approaches. This aligns with an umbrella review by Kohler et al. (2023) on population-based PA promotion with a focus on health equity, which found that the included reviews were unable to demonstrate the impact of community-wide approaches on health equity [24].

Most school-based policies reduced inequities, emphasizing the potential of these types of policies to promote equity in PA. These findings align with a review by Olstad et al. (2017) that examined the impact of both organizational and governmental school policies on health behaviours among socially disadvantaged children, suggesting that similar mechanisms may be at play, even though organizational policies were not included in this review [61]. Notably, three of the school policies focused on multi-component strategies which highlights their potential within the school setting. However, it should be taken into account that most results were obtained for low SES populations and not across SES populations, similar to community-wide policies. These findings give meaningful insights into the policy effects on inequities, but future research may want to investigate how these community-wide and school-related policy effects relate to findings in higher SES individuals.

Both the mass media and transport domains were primarily composed of studies that included results across SES groups, representing stronger evidence. Based on our assessment, both domains had a mean quality score of 1.9 (see Additional file 4). Within the transport domain, most studies incorporating cross SES results indicated either a reduction in inequities or no difference, particularly with respect to transport-related PA. These findings are consistent with existing evidence suggesting that individuals with lower SES engage in higher levels of transport-related PA [12], potentially making them more responsive to transport-related policies than high SES individuals (or at least equally responsive). Overall, media campaigns had no impact on PA inequities, showing similar effects across SES groups. This suggests that mass media campaigns do not disproportionally benefit or disadvantage lower SES populations, making them neutral.

Notably, for the urban design policies, from the limited number of studies reporting cross SES results, a relatively high number of increased inequities were reported. However, these effects may not stem from the types of policies themselves. Some policies that had a more favourable impact on higher SES groups than on lower SES groups (i.e., cross SES results) shared characteristics with those that had a positive or neutral effect in low SES populations. It has been highlighted in political science that once the goals and contents of a policy are officially decided, policy implementation – i.e. "the effort, knowledge, and resources devoted to translating a policy decision into action" – becomes important [109]. Studies across domains suggest that barriers such as insufficient resources and shifting priorities can impede implementation, limiting policy impact on PA. For example, the lack of extra funding for the healthy district approach – activities to improve the health of the local population as an addition to the urban regeneration programme (community-wide) – has been shown to hamper policy implementation [71]. Similarly, budget cuts affecting park renovations have led to reduced programming (urban design) [110]. Furthermore, new leisure centers’ equity goals were undermined by a decline in long-term commitment to equity (sport and recreation for all) [99]. These cases highlight the risks of suboptimal implementation; thus, concluding that a policy is (in)effective without considering implementation barriers may be misleading. Additionally, policy effectiveness is highly context-specific, and what works in one setting may not work in another [111]. This is especially relevant when comparing high- and low-income countries [112]. Most of the reviewed studies were conducted in high-income contexts, where lower socioeconomic groups tend to be less physically active. However, in middle- and low-income countries, the relationship between SES and PA may differ [112], emphasizing the importance of local context and the limitations of applying policies across diverse settings. Monitoring and analysing both the policy context and implementation processes, including the policy instruments utilized, is crucial for interpreting results accurately and informing future policymaking.

### Nature of policy instruments

Evidence is growing on the policy instruments deemed to be most effective for shaping health behaviours. As seen in the tobacco and alcohol policy field [113, 114], using more coercive policy instruments that compel compliance (i.e., the hardest instruments) or make certain actions easier or more difficult (i.e., medium hard instruments) may be especially effective for increasing PA levels in comparison to the abundance of persuasive strategies or ‘soft’ policy instruments (e.g., nudging) currently used to address physical inactivity [115]. Based on our findings, it is reasonable to assume that a limited number of coercive policy instruments are used to support PA in an equitable way. Economic and fiscal policy instruments were identified across domains (e.g., road pricing, free bus passes, free sport), while regulatory instruments were linked primarily to mandatory PE, with most evidence indicating that policies using these instruments tend to be equitable. Other policies may also have incorporated coercive measures, though these are not always clearly identifiable from policy descriptions. For example, it is sometimes unclear whether urban design improvements stem from regulations or voluntary agreements. Similar trends have been observed in other public health policy areas. A study on smoking showed that individuals from lower SES groups, who typically have higher smoking rates, were more responsive than their higher SES counterparts to price/tax increases — medium-strength instruments — highlighting their potential to reduce inequities [42]. Similar effects have been observed for food taxes [41]. While harder instruments like road pricing may enhance equity in PA by encouraging bus ridership among low-income populations, they can also raise ethical concerns by restricting the choices of low SES individuals more than those of higher SES groups. Specifically, road pricing could disproportionally burden low-income individuals who rely on cars, for example when public transport options are limited or unreliable [116]. These potential trade-offs must be carefully considered in policy design. Further research is needed to better understand how different policy instruments contribute to addressing inequities in PA.

### Individual agency in PA policy

Moreover, policies requiring individual agency (i.e., personal resources and effort) may widen inequities, whereas structural policies might yield equitable outcomes. A recent review by Mackenbach et al. (2024) on population-based, low-agency policies for reducing the burden of type 2 diabetes, including those targeting risk factors such as dietary intake and PA, found some evidence of favourable equity effects [108]. In the context of PA, a high-agency policy programme would, for example, require individuals to proactively seek out information, submit applications, and maintain ongoing participation. In contrast, school-based PE can be considered a low-agency policy, as it is universally implemented and affects all students without requiring individual initiative. Based on the domains, low-agency policies appear to be more common in urban design, transport, and schools. Transport and school policies tended to support equity, whereas the impact of urban design policies on inequities was more inconsistent. On the other hand, policies in the community-wide, mass media, healthcare, sport and recreation for all, and workplace domains tend to require higher levels of individual agency, though this may vary depending on the specific details of the policy—particularly in the community-wide domain, as reflected in the included policies. Given this, the inequitable impacts observed for urban design policies and the equitable impact of mass media campaigns are somewhat unexpected. This suggests that being low-agency alone may not be sufficient for urban design policies to ensure equitable outcomes; additional strategies (e.g., safety improvements) may be necessary to enhance their impact and promote equity in PA. Interestingly, mass media campaigns can be integrated into broader community-wide initiatives [117]. Given the equitable effects observed in the community-wide domain, similar mechanisms may also play a role in shaping outcomes within the mass media domain.

### Gaps in the evidence

Some ISPAH domains were underrepresented in this review, with little to no evidence on policies in the healthcare and workplace domains. This gap may be due to an actual lack of policies in these areas or to the absence of evaluations for the existing ones. It is likely that healthcare and workplace policies are predominantly designed at the organizational level (e.g., hospital or workplace-specific policies), which fell outside the scope of this review as we focused specifically on governmental action. For example, an umbrella review by Gelius et al. 2020 on effective policies for promoting PA included only one review addressing the workplace setting [33], which examined organizational workplace policies rather than public policies targeting workplaces [118]. Furthermore, evaluating public policies in workplace and healthcare settings may present unique methodological challenges, which could also contribute to the limited evidence base.

Second, there is a gap in strong evidence capable of accurately identifying the impact of policies on inequities. The findings suggest a distinction between results reported specifically for low SES populations and those observed across SES groups. In general, results from studies including low SES communities tend to be somewhat more positive. In contrast, results across SES groups more often indicated increased inequities. One possible explanation is that policies specifically designed for low-SES populations are often more tailored to their contexts and needs, providing more benefits. In contrast, policies implemented across SES groups may be more generic and less responsive to the unique barriers faced by disadvantaged populations. Another possible explanation is that, even when a policy is designed for and/or implemented in lower SES areas, individuals from higher SES backgrounds may disproportionately benefit. Their greater capacity to access, engage with, and sustain the policy may lead to more favourable outcomes in these groups. As a result, stratified analyses can reveal less positive patterns than those seen in population-level findings for lower SES groups, potentially masking inequities in reach or effectiveness. Importantly, apparent reductions in inequities can even be misleading if reach is not considered. This is illustrated by Spence et al. (2010) [119], who found that low-income individuals who claimed the Children’s Fitness Tax Credit reported increased PA. However, because most low-income individuals did not claim the credit, the policy’s potential to reduce inequities at the population level will be limited. These nuances emphasize the importance of designing and implementing policies in ways that effectively reach and support those most in need. Therefore, studies that analyse differential effects for SES provide the most accurate insights into reductions in inequities—specifically when low SES populations benefit while high SES groups do not—compared to studies focusing solely on low SES populations. The overall lack of studies on equity impacts of PA policies is recognized by previous reviews, underscoring the relevance to consider equity during the design, implementation and evaluation of policies [24, 108].

### Strengths and limitations

A strength of this review is the application of a robust and comprehensive method to capture a broad range of policies, providing a highly relevant base for future research and policy making. To categorize policies, we used the ISPAH’s eight investments that work for changing PA behaviour [120], and additional categories were introduced to ensure inclusivity and relevance. Moreover, we adopted a systematic approach to literature identification, screening, and data extraction.

Meanwhile, this study also has several limitations. The first limitation is the heterogeneity in policies, definitions of socioeconomic disadvantage, study designs, and outcome measures. This variation made it impossible to conduct a meta-analysis, thereby limiting the strength of the findings and the ability to draw conclusions about the impact of PA policies on inequities in PA. Nonetheless, similar to other reviews in related fields, we narratively summarized the results [56, 62, 121]. Furthermore, grey literature was not included in our search. While previous reviews suggest that grey literature can provide valuable insights into policy evaluation [122], we chose to focus exclusively on peer-reviewed studies to ensure at least a minimum level of evidence quality. The substantial number of studies identified in this review suggests that this approach still provided a sufficiently comprehensive overview of the available evidence. After pre-registering the review protocol with PROSPERO, we made some modifications that can be found in Additional file 7, including their rationales. Another protocol-related limitation concerns the timing of our initial registration (July 15, 2024), which ideally should have occurred before the start of the screening phase (May 17, 2024) to ensure full transparency. However, in line with PROSPERO’s eligibility criteria, data extraction had not been initiated at the time of registration [123]. Finally, we included studies of lower quality; therefore, the conclusions should be interpreted considering the RoB assessments. As policy evaluations often rely on natural experiments, it is important not to assess methodological rigor in isolation for these types of studies, but to look beyond RoB assessments and consider the broader study context [124]. Quality can also be highly domain-dependent: some domains allow for more controlled conditions, which can give a misleading impression of study relevance in domains where controlled conditions are less feasible.

## Conclusions

This systematic review shows mixed outcomes across various sectors in reducing PA-related socioeconomic inequities, with the key encouraging takeaway being that most policies did not increase inequities. Community-wide, transport, school-based, and mass media policies most frequently appeared to reduce inequities in PA or to benefit high and low SES populations equally. Specifically, infrastructure policies and financial incentives supporting active transport, multi-component school-based PA and health programmes, school PE policies, and mass media campaigns showed potential for reducing inequities or producing equal effects across SES groups. In contrast, the impact of urban design and sport and recreation for all policies was less clear, with the evidence for sport for all policies being of lower quality. Due to limited evidence, no conclusions can be made regarding workplace and healthcare policies. Future research should focus on understanding the mechanisms behind these outcomes, considering the knowledge from political science, the policy instruments used, and the levels of individual agency. Additionally, examining the effects of policies across different SES groups will be crucial to better inform the development of equitable PA policies.

## Supplementary Information

Additional file 1. PRISMA 2020 checklist.

Additional file 2. Search Strategy.

Additional file 3. Policies by ISPAH domain.

Additional file 4. Risk of Bias assessments.

Additional file 5. Summary of public policies that have an impact on inequity in PA, by policy domains.

Additional file 6. Overview of study results.

Additional file 7. Adapted PROSPERO protocol.

## Data Availability

No datasets were generated or analysed during the current study.

## Abbreviations

BSS: Bicycle Sharing System
GAPPA: Global Action Plan for Physical Activity
IBSS: International Bibliography of the Social Sciences
ISPAH: International Society for Physical Activity and Health
PA: Physical Activity
PE: Physical Education
RCT: Randomized Controlled Trial
RoB: Risk of Bias
SES: Socioeconomic Status
WHO: World Health Organization
