# Supplementary material for: The impact of public policy on socioeconomic equity in physical activity: a systematic review
Source: Int J Behav Nutr Phys Act. 2026 Feb 4;23:20. doi: 10.1186/s12966-026-01880-6 (PMC12964968; doi:10.1186/s12966-026-01880-6)
Supplement: Supplementary file 2 — Additional file 2. Search Strategy. [file 12966_2026_1880_MOESM2_ESM.docx]

Additional file 2: Search Strategy

### Search Histories and Search Details May 7, 2024

| **Search** | **Search Query PubMed, May 7 2024** | **Results** |
| --- | --- | --- |
| #1 | "Policy"[Mesh] OR "Policy Making"[Mesh] OR "Legislation as Topic"[Mesh] OR policy[tiab] OR policies[tiab] OR "national framework*"[tiab] OR legislation*[tiab] OR strateg*[tiab] | 2,253,457 |
| #2 | "Exercise"[Mesh] OR "Sedentary Behavior"[Mesh] OR "Sports"[Mesh] OR "Physical Education and Training"[Mesh] OR "Leisure Activities"[Mesh] OR "physical activ*"[tiab] OR "physical inactiv*"[tiab] OR sedentar*[tiab] OR "active life*"[tiab] OR "inactive life*"[tiab] OR "active liv*"[tiab] OR "inactive liv*"[tiab] OR "sitting"[tiab] OR sport*[tiab] OR "physical education"[tiab] OR bike*[tiab] OR bicycl*[tiab] OR cycl*[tiab] OR walk*[tiab] OR "active transport*"[tiab] OR "leisure activit*"[tiab] | 2,115,611 |
| #3 | "Health Disparate Minority and Vulnerable Populations"[Mesh] OR "Vulnerable Populations"[Mesh] OR "Health Inequities"[Mesh] OR "Health Equity"[Mesh] OR "Low Socioeconomic Status"[Mesh] OR "vulnerable population*"[tiab] OR "vulnerable group*"[tiab] OR deprived[tiab] OR disadvantaged[tiab] OR equit*[tiab] OR inequit*[tiab] OR inequalit*[tiab] OR equalit*[tiab] OR "health dispar*"[tiab] OR "low socioeconomic*"[tiab] OR "low socio-economic*"[tiab] OR "lower socioeconomic*"[tiab] OR "lower socio-economic*"[tiab] OR underprivileged[tiab] OR underserved[tiab] OR marginali*[tiab] | 423,660 |
| #4 | "Program Evaluation"[Mesh] OR "Evaluation Study" [Publication Type] OR "Cost-Effectiveness Analysis"[Mesh] OR evaluat*[tiab] OR impact*[tiab] OR apprais*[tiab] OR effect*[tiab] OR assess*[tiab] | 14,274,586 |
| #5 | #1 AND #2 AND #3 AND #4 | 3,455 |
| #6 | #5 NOT (("Animals"[Mesh] NOT "Humans"[Mesh]) NOT ("in vitro"[tiab] OR "in vivo"[tiab] OR trial[ti] OR "clinical trial"[pt])) | 3,422 |

| **Search** | **Search Query Embase.com, May 7 2024** | **Results** |
| --- | --- | --- |
| #1 | 'policy'/exp OR (policy OR policies OR "national framework*" OR legislation* OR strateg*):ti,ab,kw | 2,628,617 |
| #2 | 'exercise'/exp OR 'sedentary lifestyle'/exp OR 'sport'/exp OR 'physical education'/exp OR 'physical activity'/exp OR 'active transport'/exp OR 'leisure'/exp OR ('physical activ*' OR 'physical inactiv*' OR sedentar* OR 'active life*' OR 'inactive life*' OR 'active liv*' OR 'inactive liv*' OR 'sitting' OR sport* OR 'physical education' OR bike* OR bicycl* OR cycl* OR walk* OR 'active transport*' OR 'leisure activit*'):ti,ab,kw | 3,095,765 |
| #3 | 'vulnerable population'/exp OR 'diversity, equity and inclusion'/exp OR 'low socioeconomic status'/exp OR ('vulnerable population*' OR 'vulnerable group*' OR deprived OR disadvantaged OR equit* OR inequit* OR inequalit* OR equalit* OR 'health dispar*' OR 'low socioeconomic*' OR 'low socio-economic*' OR 'lower socioeconomic*' OR 'lower socio-economic*' OR underprivileged OR underserved OR marginali*):ti,ab,kw | 324,658 |
| #4 | 'program evaluation'/exp OR 'evaluation study'/exp OR 'cost effectiveness analysis'/exp OR (evaluat* OR impact* OR apprais* OR effect* OR assess*):ti,ab,kw | 18,574,086 |
| #5 | #1 AND #2 AND #3 AND #4 | 4,339 |
| #6 | #5 NOT ('conference abstract'/it OR 'conference review'/it) | 3,062 |
| #7 | #6 NOT ([animals]/lim NOT [humans]/lim) NOT (('in vitro' OR 'in vivo'):ti,ab,kw OR trial:ti) | 2,868 |

| **Search** | **Search Query SportDiscus/Ebsco, May 7 2024** | **Results** |
| --- | --- | --- |
| S1 | TI (policy OR policies OR "national framework*" OR legislation* OR strateg*) OR AB (policy OR policies OR "national framework*" OR legislation* OR strateg*) OR KW (policy OR policies OR "national framework*" OR legislation* OR strateg*) | 110,828 |
| S2 | DE ("PHYSICAL activity" OR "EXERCISE" OR "PHYSICAL education" OR "SPORTS" OR "SEDENTARY lifestyles" OR "SEDENTARY behavior" OR "CYCLING" OR "BICYCLE commuting" OR "WALKING" OR"LEISURE") OR TI ("physical activ*" OR "physical inactiv*" OR sedentar* OR "active life*" OR "inactive life*" OR "active liv*" OR "inactive liv*" OR "sitting" OR sport* OR "physical education" OR bike* OR bicycl* OR cycl* OR walk* OR "active transport*" OR "leisure activit*") OR AB ("physical activ*" OR "physical inactiv*" OR sedentar* OR "active life*" OR "inactive life*" OR "active liv*" OR "inactive liv*" OR "sitting" OR sport* OR "physical education" OR bike* OR bicycl* OR cycl* OR walk* OR "active transport*" OR "leisure activit*") OR KW ("physical activ*" OR "physical inactiv*" OR sedentar* OR "active life*" OR "inactive life*" OR "active liv*" OR "inactive liv*" OR "sitting" OR sport* OR "physical education" OR bike* OR bicycl* OR cycl* OR walk* OR "active transport*" OR "leisure activit*") | 812,676 |
| S3 | TI ("vulnerable population*" OR "vulnerable group*" OR deprived OR disadvantaged OR equit* OR inequit* OR inequalit* OR equalit* OR "health dispar*" OR "low socioeconomic*" OR "low socio-economic*" OR "lower socioeconomic*" OR "lower socio-economic*" OR underprivileged OR underserved OR marginali*) OR AB ("vulnerable population*" OR "vulnerable group*" OR deprived OR disadvantaged OR equit* OR inequit* OR inequalit* OR equalit* OR "health dispar*" OR "low socioeconomic*" OR "low socio-economic*" OR "lower socioeconomic*" OR "lower socio-economic*" OR underprivileged OR underserved OR marginali*) OR KW ("vulnerable population*" OR "vulnerable group*" OR deprived OR disadvantaged OR equit* OR inequit* OR inequalit* OR equalit* OR "health dispar*" OR "low socioeconomic*" OR "low socio-economic*" OR "lower socioeconomic*" OR "lower socio-economic*" OR underprivileged OR underserved OR marginali*) | 19,423 |
| S4 | TI (evaluat* OR impact* OR apprais* OR effect* OR assess*) OR AB (evaluat* OR impact* OR apprais* OR effect* OR assess*) OR KW(evaluat* OR impact* OR apprais* OR effect* OR assess*) | 590,484 |
| S5 | S1 AND S2 AND S3 AND S4 | 669 |
| S6 | S5 NOT TI ("trial") | 661 |

| **Search** | **Search Query Cinahl Plus/Ebsco, May 7 2024** | **Results** |
| --- | --- | --- |
| S1 | MH ("Public Policy+" OR "Policy Making" OR "Policy Studies+" OR "Legislation+") OR TI (policy OR policies OR "national framework*" OR legislation* OR strateg*) OR AB (policy OR policies OR "national framework*" OR legislation* OR strateg*) | 647,086 |
| S2 | MH ("Leisure Activities+" OR "Physical Activity" OR "Physical Education and Training+" OR "Exercise+" OR "Sports+" OR "Life Style, Sedentary+") OR TI ("physical activ*" OR "physical inactiv*" OR sedentar* OR "active life*" OR "inactive life*" OR "active liv*" OR "inactive liv*" OR "sitting" OR sport* OR "physical education" OR bike* OR bicycl* OR cycl* OR walk* OR "active transport*" OR "leisure activit*") OR AB ("physical activ*" OR "physical inactiv*" OR sedentar* OR "active life*" OR "inactive life*" OR "active liv*" OR "inactive liv*" OR "sitting" OR sport* OR "physical education" OR bike* OR bicycl* OR cycl* OR walk* OR "active transport*" OR "leisure activit*") | 488,840 |
| S3 | MH ("Low Socioeconomic Status" OR "Diversity, Equity, Inclusion") OR TI ("vulnerable population*" OR "vulnerable group*" OR deprived OR disadvantaged OR equit* OR inequit* OR inequalit* OR equalit* OR "health dispar*" OR "low socioeconomic*" OR "low socio-economic*" OR "lower socioeconomic*" OR "lower socio-economic*" OR underprivileged OR underserved OR marginali*) OR AB ("vulnerable population*" OR "vulnerable group*" OR deprived OR disadvantaged OR equit* OR inequit* OR inequalit* OR equalit* OR "health dispar*" OR "low socioeconomic*" OR "low socio-economic*" OR "lower socioeconomic*" OR "lower socio-economic*" OR underprivileged OR underserved OR marginali*) | 110,283 |
| S4 | MH ("Program Evaluation" OR "Evaluation" OR "Evaluation Research+") OR TI (evaluat* OR impact* OR apprais* OR effect* OR assess*) OR AB (evaluat* OR impact* OR apprais* OR effect* OR assess*) | 2,868,084 |
| S5 | S1 AND S2 AND S3 AND S4 | 1,285 |
| S6 | S5 NOT TI (trial) | 1,223 |

| **Search** | **Search Query Web of Science Core Collection, May 7 2024** | **Results** |
| --- | --- | --- |
| #1 | TS = (policy OR policies OR "national framework*" OR legislation* OR strateg*) | 4,361,400 |
| #2 | TS = ("physical activ*" OR "physical inactiv*" OR sedentar* OR "active life*" OR "inactive life*" OR "active liv*" OR "inactive liv*" OR "sitting" OR sport* OR "physical education" OR bike* OR bicycl* OR cycl* OR walk* OR "active transport*" OR "leisure activit*") | 3,924,976 |
| #3 | TS = ("vulnerable population*" OR "vulnerable group*" OR deprived OR disadvantaged OR equit* OR inequit* OR inequalit* OR equalit* OR "health dispar*" OR "low socioeconomic*" OR "low socio-economic*" OR "lower socioeconomic*" OR "lower socio-economic*" OR underprivileged OR underserved OR marginali*) | 881,469 |
| #4 | TS = (evaluat* OR impact* OR apprais* OR effect* OR assess*) | 24,283,125 |
| #5 | #1 AND #2 AND #3 AND #4 | 6,608 |
| #6 | #5 NOT TI=("trial") | 6,449 |

| **Search** | **Search Query Scopus, May 7 2024** | **Results** |
| --- | --- | --- |
| #1 | TITLE-ABS ({policy}OR {policies} OR "national framework*" OR legislation* OR strateg*) OR AUTHKEY ({policy} OR {policies} OR "national framework*" OR legislation* OR strateg*) | 5,963,676 |
| #2 | TITLE-ABS ("physical activ*" OR "physical inactiv*" OR sedentar* OR "active life*" OR "inactive life*" OR "active liv*" OR "inactive liv*" OR "sitting" OR sport* OR "physical education" OR bike* OR bicycl* OR cycl* OR walk* OR "active transport*" OR "leisure activit*") OR AUTHKEY ("physical activ*" OR "physical inactiv*" OR sedentar* OR "active life*" OR "inactive life*" OR "active liv*" OR "inactive liv*" OR "sitting" OR sport* OR "physical education" OR bike* OR bicycl* OR cycl* OR walk* OR "active transport*" OR "leisure activit*") | 4,606,396 |
| #3 | TITLE-ABS ("vulnerable population*" OR "vulnerable group*" OR deprived OR disadvantaged OR equit* OR inequit* OR inequalit* OR equalit* OR "health dispar*" OR "low socioeconomic*" OR "low socio-economic*" OR "lower socioeconomic*" OR "lower socio-economic*" OR underprivileged OR underserved OR marginali*) OR AUTHKEY ("vulnerable population*" OR "vulnerable group*" OR deprived OR disadvantaged OR equit* OR inequit* OR inequalit* OR equalit* OR "health dispar*" OR "low socioeconomic*" OR "low socio-economic*" OR "lower socioeconomic*" OR "lower socio-economic*" OR underprivileged OR underserved OR marginali*) | 899,675 |
| #4 | TITLE-ABS (evaluat* OR impact* OR apprais* OR effect* OR assess*) OR AUTHKEY (evaluat* OR impact* OR apprais* OR effect* OR assess*) | 33,592,198 |
| #5 | #1 AND #2 AND #3 AND #4 | 5,193 |
| #6 | TITLE ({trial}) | 353,186 |
| #7 | #5 AND NOT #6 | 5,060 |

| **Search** | **Search Query International Bibliography of the Social Sciences/ProQuest, May 7 2024** | **Results** |
| --- | --- | --- |
| #1 | MAINSUBJECT.EXACT("Health care policy") OR ti,ab(policy OR policies OR "national framework*" OR legislation* OR strateg*) | 814,678 |
| #2 | MAINSUBJECT.EXACT("Exercise" OR "Sports" OR "Physical activity" OR "Sedentary" OR "Physical education" OR "Leisure") OR ti,ab("physical activ*" OR "physical inactiv*" OR sedentar* OR "active life*" OR "inactive life*" OR "active liv*" OR "inactive liv*" OR "sitting" OR sport* OR "physical education" OR bike* OR bicycl* OR cycl* OR walk* OR "active transport*" OR "leisure activit*") | 101,613 |
| #3 | MAINSUBJECT.EXACT("Equity" OR "Inequality" OR "Health disparities" OR "Low status people") OR ti,ab("vulnerable population*" OR "vulnerable group*" OR deprived OR disadvantaged OR equit* OR inequit* OR inequalit* OR equalit* OR "health dispar*" OR "low socioeconomic*" OR "low socio-economic*" OR "lower socioeconomic*" OR "lower socio-economic*" OR underprivileged OR underserved OR marginali*) | 193,138 |
| #4 | MAINSUBJECT.EXACT("Appraisal" OR "Impact analysis" OR "Cost analysis" OR "Strategic impact assessment") OR ti,ab(evaluat* OR impact* OR apprais* OR effect* OR assess*) | 1,022,793 |
| #5 | [S1] AND [S2] AND [S3] AND [S4] | 674 |
