# Supplementary material for: The impact of public policy on socioeconomic equity in physical activity: a systematic review
Source: Int J Behav Nutr Phys Act. 2026 Feb 4;23:20. doi: 10.1186/s12966-026-01880-6 (PMC12964968; doi:10.1186/s12966-026-01880-6)
Supplement: Supplementary file 3 — Additional file 3. Policies by ISPAH domain. [file 12966_2026_1880_MOESM3_ESM.docx]

Additional file 3: Policies by ISPAH domain

| **Policy domain** | **Policy** |
| --- | --- |
| Schools | - Physical education policies - School-related physical activity programmes - Shared use agreements (of school spaces) - Safe active travel (to school) policies - Policies for enhancing the physical education workforce |
| Transport | - Active transport (walking, cycling, and wheeling) infrastructure policies - Traffic calming measures - Vehicle traffic reduction policies - Active transport promotion programmes (e.g., bicycle sharing programmes)   - Including public transport - Policies improving public transportation |
| Urban Design | - Urban layout policies (layout of buildings, streets, and public spaces) - Design of communal areas (e.g., parks, squares, and playgrounds) - Visual quality standards (architectural style, landscaping, and street furniture) - Mixed land use development policies |
| Healthcare | - Exercise prescription programmes - Training programmes/tools for health professionals (e.g., improving assessment, advice giving and referral) - Financing clinical preventive services - Physical activity programmes for patient groups |
| Mass media | - Mass media campaigns (print, audio and electronic media, digital and social media, outdoor billboards and posters, public relations, point of decision prompts and mass-distribution of information.) - Can be complemented with community initiatives |
| Community-wide | - Multi-approach/setting policy programmes to tackle physical inactivity: this suggests that community-wide initiatives could incorporate elements from various sectors (e.g., Transport, Education and Urban Design). |
| Sport and Recreation for all | - Policies for improving/increasing sport and recreation opportunities (e.g., financial objectives) - Policies targeting the development and improvement of sport and recreation spaces - Mass sport and recreation events - Visibility of elite sportspeople - Policies for enhancing the sporting workforce and delivery organizations (with the exception of physical education teachers (Education domain)) |
| Workplace | - Policies for adapting the workplace environment to promote physical activity - Active commuting programmes - Workplace wellness/PA programmes - Paid time for exercise for employees (or other health incentives) and/or flexible time for physical activity |
