# Supplementary material for: The impact of public policy on socioeconomic equity in physical activity: a systematic review
Source: Int J Behav Nutr Phys Act. 2026 Feb 4;23:20. doi: 10.1186/s12966-026-01880-6 (PMC12964968; doi:10.1186/s12966-026-01880-6)
Supplement: Supplementary file 4 — Additional file 4. Risk of Bias assessments. [file 12966_2026_1880_MOESM4_ESM.docx]

Additional file 4: Risk of Bias assessment**^1^**

| **Author/year** | **Randomisation/**  **control** | **Exposure** | **Representative-ness** | **Comparability** | **Attrition or sample size** | **Criteria met (out of 5)** | **Applicable criteria met (%)** |
| --- | --- | --- | --- | --- | --- | --- | --- |
| **Community-wide** | | | | | | | |
| Andersen et al., 2017 | 0 | 1 | 1 | NA | 1 | 3 | 75 |
| Aytur et al., 2008 | 0 | 1 | 1 | NA | NA | 2 | 67 |
| Bijlani et al., 2024 | 0 | 0 | 1 | NA | 1 | 2 | 50 |
| Boelens et al., 2022 | 1 | 1 | 0 | 1 | 1 | 4 | 80 |
| Buscemi et al., 2019 | 1 | 1 | 0 | 1 | 1 | 4 | 80 |
| Cheadle et al., 2018 | 0 | 0 | 0 | NA | 1 | 1 | 25 |
| Derose et al., 2019 | 1 | 1 | 0 | 1 | 1 | 4 | 80 |
| Heath and Bilderback, 2019 | 0 | 0 | 1 | NA | 1 | 2 | 50 |
| Herens et al., 2016 | 1 | 1 | 0 | 1 | 0 | 3 | 60 |
| Jalaludin et al., 2012 | 0 | 0 | 1 | NA | 1 | 2 | 50 |
| Ruijsbroek et al., 2022 | 1 | 0 | 1 | 1 | 1 | 4 | 80 |
| Kramer et al., 2014 | 1 | 1 | 1 | 1 | 1 | 5 | 100 |
| Mean domain score |  |  |  |  |  | 3 |  |
| **Transport** | | | | | | | |
| Adams and Cavill, 2015 | 0 | 0 | 0 | NA | 1 | 1 | 25 |
| Agarwal and Koo, 2016 | 1 | 1 | 1 | 1 | 1 | 5 | 100 |
| Chang et al., 2017 | 1 | 0 | 0 | 1 | 1 | 3 | 60 |
| Cook et al., 2016 | 0 | 0 | 0 | NA | 1 | 1 | 25 |
| Goodman and Cheshire, 2014a | 0 | 0 | 1 | NA | 1 | 2 | 50 |
| Iroz-Elardo et al., 2020 | 0 | NA | NA | NA | NA | 0 | 0 |
| Karlstrom and Franklin, 2009 | 1 | 0 | 1 | 1 | 1 | 4 | 80 |
| Martin et al., 2021 | 0 | 0 | 1 | NA | 1 | 2 | 50 |
| Norwood et al., 2014 | 1 | 0 | 0 | 1 | 1 | 3 | 60 |
| Panter et al., 2017 | 0 | 0 | 0 | NA | 0 | 0 | 0 |
| Mean domain score |  |  |  |  |  | 1.9 |  |
| **Urban Design** | | | | | | | |
| Brownson et al., 2000 | 0 | 0 | 1 | NA | NA | 1 | 33 |
| Dulin-Keita et al., 2015 | 0 | 0 | 0 | NA | 1 | 1 | 25 |
| Kodali et al., 2024 | 1 | 1 | 1 | 1 | 1 | 5 | 100 |
| Lopes et al., 2023 | 0 | 0 | 0 | NA | 1 | 1 | 25 |
| Zenk et al., 2021 | 1 | 1 | 1 | 1 | 0 | 4 | 80 |
| Mean domain score |  |  |  |  |  | 2.4 |  |
| **Schools** | | | | | | | |
| Barbosa Filho et al., 2019 | 1 | 1 | 1 | 1 | 1 | 5 | 100 |
| Carson et al., 2014 | 1 | 0 | 0 | 1 | 0 | 2 | 40 |
| Mendoza et al., 2009 | 1 | 1 | 1 | 0 | 1 | 4 | 80 |
| Nathan et al., 2015 | 0 | 0 | 1 | NA | 1 | 2 | 50 |
| Sutherland et al., 2016 | 1 | 1 | 1 | 1 | 0 | 4 | 80 |
| Van der Ploeg et al., 2014 | 1 | 1 | 1 | 1 | 1 | 5 | 100 |
| Mean domain score |  |  |  |  |  | 3.7 |  |
| **Sport for all** | | | | | | | |
| Andrade et al., 2018 | 1 | 0 | 1 | 1 | NA | 3 | 75 |
| Candio et al., 2020 | 0 | 0 | 0 | NA | 0 | 0 | 0 |
| Higgerson et al., 2018a | 1 | 1 | 1 | 1 | NA | 4 | 100 |
| Hoekman et al., 2017 | 0 | 0 | 1 | NA | NA | 1 | 33 |
| Rabiee et al., 2015 | 0 | 0 | 0 | NA | NA | 0 | 0 |
| Reilly et al., 2021 | 0 | 0 | 0 | NA | 0 | 0 | 0 |
| Taylor et al., 2011 | 0 | 0 | 0 | NA | 1 | 1 | 25 |
| Virmasalo et al., 2023 | 0 | 0 | 1 | NA | NA | 1 | 33 |
| Williams, 2017 | 0 | 0 | 0 | NA | 0 | 0 | 0 |
| Mean domain score |  |  |  |  |  | 1.1 |  |
| **Mass Media** | | | | | | | |
| Bauman et al., 2001 | 1 | 1 | 1 | 1 | 1 | 5 | 100 |
| Booth et al., 1992 | 0 | 0 | 1 | NA | 1 | 2 | 50 |
| Croker et al., 2012 | 1 | 1 | 0 | 1 | 0 | 3 | 60 |
| Hillsdon et al., 2001 | 0 | 0 | 0 | NA | 0 | 0 | 0 |
| Leavy et al., 2013 | 0 | 1 | 1 | NA | 1 | 3 | 75 |
| Leavy et al., 2014 | 0 | 0 | 0 | NA | 1 | 1 | 25 |
| Owen et al., 1995 | 0 | 0 | 0 | NA | 1 | 1 | 25 |
| Pena-Y-Lillo and Lee, 2019 | 0 | 0 | 0 | NA | NA | 0 | 0 |
| Mean domain score |  |  |  |  |  | 1.9 |  |
| **Healthcare** | | | | | | | |
| Tomioka et al., 2012 | 0 | 0 | 0 | NA | 1 | 1 | 25 |
| **Childcare** | | | | | | | |
| Esquivel et al., 2016 | 1 | 0 | 1 | 1 | 1 | 4 | 80 |
| Tomayko et al., 2017 | 0 | 0 | 0 | NA | 1 | 1 | 25 |
| Yoong et al., 2016 | 0 | 0 | 1 | NA | 1 | 2 | 50 |
| Mean domain score |  |  |  |  |  | 2.3 |  |
| **Social** | | | | | | | |
| Vahid Shahidi et al., 2019 | 1 | 0 | 1 | 1 | 1 | 4 | 80 |

^1^ **Randomisation criteria**: Were participants, groups, or areas randomly allocated to intervention and control status, or was there a control group?

**Exposure criteria**: Did authors show both that participants did not receive concurrent intervention that could have differentially influenced physical activity behaviour in intervention and control groups and that control group was not contaminated by receiving part or all of intervention being studied?

**Representativeness criteria**: Were study samples randomly recruited from study population with response rate of at least 60%, or were they otherwise shown to be representative of study population?

**Comparability criteria**: Were baseline characteristics of intervention and control groups, populations, or areas comparable, or if there were important differences in potential confounders at baseline, were these appropriately adjusted for in analysis?

- Comparability can be demonstrated by indicating the control group was recruited either from the same population as the intervention group, or from a neighbouring area chosen for its similarity (i.e. without showing that baseline characteristics are similar between intervention and control groups, or adjusting for any differences in such characteristics).

**Attrition or sample size criteria**: Were outcomes studied in a sample of respondents with attrition/dropout rate of less than 30%, or were results based on repeated cross sectional design with minimum achieved sample of at least 100 participants in each wave in both intervention and control groups?
