# Supplementary material for: The impact of public policy on socioeconomic equity in physical activity: a systematic review
Source: Int J Behav Nutr Phys Act. 2026 Feb 4;23:20. doi: 10.1186/s12966-026-01880-6 (PMC12964968; doi:10.1186/s12966-026-01880-6)
Supplement: Supplementary file 5 — Additional file 5. Summary of public policies that have an impact on inequity in PA, by policy domains. [file 12966_2026_1880_MOESM5_ESM.docx]

Additional file 5. Summary of public policies that have an impact on inequity in PA, by policy domains

|  |  | | **Summary of public policies’ impact on inequity in PA** | | | |  |
| --- | --- | --- | --- | --- | --- | --- | --- |
|  | *Policy* | | | *Low SES population* | *SES subgroup measure* | *Impact on inequity^1^* | *Quality rating^3^* |
| **Community-wide** | |  | | | | |  |
| Andersen et al., 2017 [1] | Urban renewal | | | Disadvantaged district |  | Area-level: Reduced | 3 |
| Aytur et al., 2007^2^ [2] | Land use and transportation plans | | |  | Income | Subgroup: No difference | Cochrane Public Health Review Group-recommended Effective Public Health Practice Project Quality Assessment Tool for Quantitative Studies: moderate |
| Aytur et al., 2008 [3] | Comprehensive land use planning | | |  | Income | Subgroup: Reduced | 2 |
| Bijlani et al., 2024 [4] | Health promotion intervention community | | | Deprived ward | Deprivation | Area-level: No difference  Subgroup: No difference | 2 |
| Boelens et al., 2022 [5] | Neighbourhood community programme including health promotion | | |  | Parental education | Subgroup: No difference | 4 |
| Buscail et al., 2016^2^ [6] | Health promotion programme community | | | Socially disadvantaged community | Professional status | Area-level: Reduced  Subgroup: no difference | Global rating according to EPHPP: 2 (moderate) |
| Buscemi et al., 2019 [7] | Health promotion intervention community | | | Low-income parents and children |  | Area-level: No difference | 4 |
| Cheadle et al., 2018 [8] | Park renovation along exercise programmes | | | Low-income community |  | Area-level: No difference | 1 |
| Derose et al., 2019 [9] | Health promotion intervention community | | | Disadvantaged in terms of SES (income, education, wealth, employment) |  | Area-level: No difference | 4 |
| Goodman et al., 2013a^2^ [6] | A mix of cycling promotion initiatives | | | Socially disadvantaged | Deprived area | Area-level: Reduced  Subgroup: Mixed (=, +) | Global rating according to EPHPP: 2 (moderate) |
| Heath and Bilderback, 2019 [10] | Policy and environmental interventions | | | Economically depressed areas |  | Area-level: Mixed (+, -) | 2 |
| Herens et al., 2016 [11] | PA programme community | | | Socially vulnerable groups | Education | Area-level: No difference  Subgroup: Increased | 3 |
| Higgerson et al., 2018b^2^ [6] | Free access to activities in leisure centres along with community outreach activities | | | Areas of deprivation | Socioeconomic group (not specified) | Area-level: Reduced  Subgroup: Mixed (=, +) | Global rating according to EPHPP: 2 (moderate) |
| Jalaludin et al., 2012 [12] | Urban renewal | | | Socially disadvantaged community |  | Area-level: No difference | 2 |
| Mohan et al., 2017^2^ [6] | Urban renewal | | | Socially disadvantaged community | Education, employment | Area-level: No difference  Subgroup: No difference | Global rating according to EPHPP: 1 (strong) |
| O’Loughlin et al., 1999^2^ [6] | Health promotion programme community | | | Socially disadvantaged community | Education | Area-level: No difference  Subgroup: No difference | Global rating according to EPHPP: 3 (weak) |
| Phillips et al., 2014^2^ [6] | Health promotion programme community | | | Socially disadvantaged community | Education, employment | Area-level: No difference  Subgroup: No difference | Global rating according to EPHPP: 1 (strong) |
| Raine et al., 2013^2^ [6] | Health promotion intervention community | | | Socially disadvantaged community |  | Area-level: Increased | Global rating according to EPHPP: 1 (strong) |
| Ruijsbroek et al., 2022 [13] | Activities to improve the health of the local population as an addition to the urban regeneration program | | | Deprived areas |  | Area-level: No difference | 4 |
| Schulz et al., 2015^2^ [6] | Walking group intervention (behaviour change program) | | | Socially disadvantaged community |  | Area-level: Reduced | Global rating according to EPHPP: 2 (moderate) |
| Tester and Baker, 2009^2^ [14] | Park renovation and a park initiative to improve family and youth involvement | | | Poor neighbourhoods |  | Area-level: Mixed (males: +,=; females: +) | [Twohig-Bennett and Jones (2018)](https://www.sciencedirect.com/science/article/pii/S0160412018331751?via%3Dihub#bb0350) tool: 9/11 (high quality) |
| Kramer et al., 2014 [15] | Area-based initiatives/ Environmental interventions | | | Deprived districts |  | Area-level: Mixed (=, +) | 5 |
| **Transport** |  | |  | | | |  |
| Adams and Cavill, 2015 [16] | Small-scale environmental improvements on pedestrian route use | | | Deprived communities |  | Area-level: Mixed (=, +, -) | 1 |
| Agarwal and Koo, 2016^2^ [17] | Road pricing | | |  | Income | Subgroup: Reduced | 5 |
| Chang et al., 2017^2^ [18] | New transit infrastructure/access (Bus rapid transit) | | |  | Education | Subgroup: Mixed (males: -, +; females: +) | 3 |
| Cook et al., 2016 [19] | Bicycle-and-pedestrian bridge that linked two separate segments of the regional greenway | | |  | Income | Subgroup: Increased | 1 |
| Coronini-Cronberg et al., 2012^2^ [2] | Free national bus pass | | |  | Home ownership | Subgroup: Mixed (=, +) | Cochrane Public Health Review Group recommended Effective Public Health Practice Project Quality Assessment Tool for Quantitative Studies: Moderate |
| Goodman and Cheshire, 2014a [20] | Extension of bicycle sharing system to deprived areas | | | Highly-deprived areas |  | Area-level: Reduced | 2 |
| Goodman and Cheshire, 2014a [20] | Doubling of bicycle sharing system prices after extension of bicycle sharing system to deprived areas | | | Highly-deprived areas |  | Area-level: Mixed (=, -) | - |
| Goodman et al., 2014b^2^ [21] | Building or improving walking and cycling routes | | |  | Income, education, employment | Subgroup: No difference | EPHPP criteria: weak |
| Iroz-Elardo et al., 2020 [22] | New housing units, higher levels of expanded transit (bus rapid transit and commuter rail) and active transportation facilities | | |  | Poverty area | Subgroup: Mixed (+, -) | 0 |
| Karlstrom and Franklin, 2009^2^ [17] | Road pricing | | |  | Income | Subgroup: No difference | 4 |
| Martin et al., 2021 [23] | Bicycle infrastructure investment | | |  | Education | Subgroup: Increased | 2 |
| Norwood et al., 2014^2^ [18] | Active transport intervention (including walking infrastructure) | | |  | Education | Subgroup: Reduced | 3 |
| Panter et al., 2017^2^ [18] | Constructing or improving routes. | | |  | Income, education | Subgroup: Mixed (=, +) | 0 |
| **Urban Design** |  | |  | | | |  |
| Bohn-Goldhaum et al., 2013^2^ [14] | Park renovation | | | Low socioeconomic neighbourhood |  | Area-level: No difference | [Twohig-Bennett and Jones (2018)](https://www.sciencedirect.com/science/article/pii/S0160412018331751?via%3Dihub#bb0350) tool: 6/11 |
| Brownson et al., 2000 [24] | Walking trail construction and promotion | | | Region with more poverty, and lower educational levels | Income, education | Area-level: Reduced  Subgroup: Reduced | 1 |
| Cohen et al., 2009^2^ [25] | Park renovation | | | Poverty area |  | Area-level: No difference | Cochrane Public Health Review Group-recommended Effective Public Health Practice Project Quality Assessment Tool for Quantitative Studies: Moderate |
| Cohen et al., 2012^2^ [14] | Park renovation | | | Poverty area |  | Area-level: No difference | [Twohig-Bennett and Jones (2018)](https://www.sciencedirect.com/science/article/pii/S0160412018331751?via%3Dihub#bb0350) tool: 7/11 |
| Cohen et al., 2014^2^ [14] | New parks | | | Poverty area |  | Area-level: No difference | [Twohig-Bennett and Jones (2018)](https://www.sciencedirect.com/science/article/pii/S0160412018331751?via%3Dihub#bb0350) tool: 9/11 (high quality) |
| Droomers et al., 2015^2^ [14] | Green interventions | | | Severely deprived neighbourhoods |  | Area-level: No difference | [Twohig-Bennett and Jones (2018)](https://www.sciencedirect.com/science/article/pii/S0160412018331751?via%3Dihub#bb0350) tool: 11/11 (high quality) |
| Dulin-Keita et al., 2015 [26] | Housing rentals and green spaces, play areas, walkways and bike paths | | | Low SES (not specified) community |  | Area-level: No difference | 1 |
| Fitzhugh et al., 2010^2^ [14] | Retrofit of an urban greenway | | | High poverty |  | Area-level: Reduced | [Twohig-Bennett and Jones (2018)](https://www.sciencedirect.com/science/article/pii/S0160412018331751?via%3Dihub#bb0350) tool: 8/11 |
| Gubbels et al., 2016^2^ [14] | Changes in greenery | | | Severely deprived neighbourhoods |  | Area-level: mixed (adolescents: =, +, adults: =)  Subgroup: No difference | [Twohig-Bennett and Jones (2018)](https://www.sciencedirect.com/science/article/pii/S0160412018331751?via%3Dihub#bb0350) tool: 11/11 (high quality) |
| Kodali et al., 2024 [27] | Park renovation | | | Low-income neighbourhoods |  | Area-level: Reduced | 5 |
| Lopes et al., 2023 [28] | Social housing policies | | |  | Income | Subgroup: Increased | 1 |
| Peschardt and Stigsdotter, 2014^2^ [14] | Park renovation | | |  | Education | Subgroup: Increased | [Twohig-Bennett and Jones (2018)](https://www.sciencedirect.com/science/article/pii/S0160412018331751?via%3Dihub#bb0350) tool: 6/11 |
| Veitch et al., 2012^2^ [14] | Park renovation | | | Most disadvantaged decile in state of Victoria |  | Area-level: Reduced | [Twohig-Bennett and Jones (2018)](https://www.sciencedirect.com/science/article/pii/S0160412018331751?via%3Dihub#bb0350) tool: 9/11 (high quality) |
| Zenk et al., 2021 [29] | Playground renovation | | |  | Income | Subgroup: Increased | 4 |
| **Schools** |  | |  | | | |  |
| Barbosa Filho et al., 2019 [30] | Health promotion intervention schools | | | Adolescents from schools in low Human Development Index areas |  | Area-level: Reduced | 5 |
| Carson et al., 2014 [31] | Professional development programme for PE teachers | | | Low-income student populations |  | Area-level: Mixed (=, +) | 2 |
| Cheadle et al., 2018 [8] | PA training for parent volunteers, teachers, or YMCA staff | | | Low-income communities |  | Area-level: Reduced | - |
| Hobin et al., 2014^2^ [2] | School PE policy | | |  | School neighbourhood disadvantage | Subgroup: Reduced | Cochrane Public Health Review Group recommended Effective Public Health Practice Project Quality Assessment Tool for Quantitative Studies: Moderate |
| Kim, 2012^2^ [2] | School PE requirements | | |  | Parental education, income | Subgroup: No difference | Cochrane Public Health Review Group recommended Effective Public Health Practice Project Quality Assessment Tool for Quantitative Studies: Moderate |
| Mendoza et al., 2009 [32] | Walking school bus (WSB) program | | | Low-income community |  | Area-level: Reduced | 4 |
| Nathan et al., 2015 [33] | State level mandatory policies and recommended programmes targeting the school environment | | |  | Socio-Economic Indexes for Australia (SEIFA) | Subgroup: No difference | 2 |
| Ridgers et al., 2007^2^ [25] | School playground renovation | | | Areas of high social and economic deprivation |  | Area-level: No difference | Cochrane Public Health Review Group-recommended Effective Public Health Practice Project Quality Assessment Tool for Quantitative Studies: Strong |
| Sutherland et al., 2016 [34] | PA intervention schools | | | Disadvantaged areas |  | Area-level: Reduced | 4 |
| Van der Ploeg et al., 2014 [35] | Health promotion intervention schools | | | Socioeconomically disadvantaged neighbourhoods |  | Area-level: Reduced | 5 |
| **Sport for all** |  | |  | | | |  |
| Andrade et al., 2018 [36] | Free access to PA activities, guided by PE professional | | | Areas of social vulnerability |  | Area-level: Reduced | 3 |
| Candio et al., 2020 [37] | Universal access to free off-peak leisure centre based exercise sessions | | |  | Deprivation | Subgroup: Reduced | 0 |
| Higgerson et al., 2018a [38] | Free swimming pool access | | | Areas of deprivation | Deprivation | Area-level: Reduced  Subgroup: Mixed (=, +) | 4 |
| Hoekman et al., 2017 [39] | Sport policy programmes | | |  | Income, education | Subgroup: Mixed (children =, adults =, -) | 1 |
| Hoekman et al., 2017 [39] | Higher municipal sport expenditure | | |  | Income, education | Subgroup: Mixed (adults =, children =, +) | - |
| Rabiee et al., 2015 [40] | Free access to leisure facilities | | | Deprived areas |  | Area-level: Reduced | 0 |
| Reilly et al., 2021 [41] | Sport voucher schemes | | | Low socioeconomic index | Socio-Economic Indexes For Australia (SEIFA) | Area-level: No difference  Subgroup: No difference | 0 |
| Taylor et al., 2011 [42] | Investment in sport and leisure facilities | | | Unemployed |  | Area-level: Increased | 1 |
| Virmasalo et al., 2023 [43] | Indoor sports restrictions | | |  | Education, employment, income | Subgroup: No difference | 1 |
| Williams, 2017 [44] | New leisure centre drifting away from initial commitments to equitable service access | | | Deprived neighbourhood |  | Area-level: Increased | 0 |
| **Mass Media** |  | |  | | | |  |
| Bauman et al., 2001^2^ [45] | Mass Media campaign | | |  | Education | Subgroup: No difference | 5 |
| Booth et al., 1992^2^ [45] | Mass Media campaign | | |  | Education | Subgroup: Reduced | 2 |
| Croker et al., 2012^2^ [45] | Mass Media campaign | | |  | Education | Subgroup: No difference | 3 |
| Hillsdon et al., 2001^2^ [45] | Mass Media campaign | | |  | Social grade (occupation) | Subgroup: No difference | 0 |
| Leavy et al., 2013^2^ [45] | Mass Media campaign | | |  | Education | Subgroup: Increased | 3 |
| Leavy et al., 2014^2^ [45] | Mass Media campaign | | |  | SES index for area, Education | Subgroup: No difference | 1 |
| Owen et al., 1995^2^ [45] | Mass Media campaign | | |  | Education | Subgroup: No difference | 1 |
| Pena-Y-Lillo and Lee, 2019 [46] | Mass Media campaign | | |  | Income, Education | Subgroup: Mixed (=, -) | 0 |
| **Healthcare** |  | |  | | | |  |
| Davison et al., 2011^2^ [25] | Integration of a community resource guide that links families with local PA resources into WIC counselling sessions with parents | | | All participants enrolled in WIC |  | Area-level: Mixed (parents =, children +) | Cochrane Public Health Review Group-recommended Effective Public Health Practice Project Quality Assessment Tool for Quantitative Studies: Moderate |
| Tomioka et al., 2012 [47] | Health promotion programme for patient group | | | Low education and income |  | Area-level: Mixed (=, +) | 1 |
| **Childcare** |  | |  | | | |  |
| Cheadle et al., 2018 [8] | Early childhood care sites making health-promoting changes | | | Low-income communities |  | Area-level: Reduced | - |
| Esquivel et al., 2016 [48] | Health promotion programme childcare services | | | Low-income children |  | Area-level: Reduced | 4 |
| Tomayko et al., 2017 [49] | A guide for improving childhood PA, along with provider training, microgrant support, and technical assistance | | | Low SES (not specified) |  | Area-level: No difference | 1 |
| Yoong et al., 2016 [50] | State specific programmes which may have facilitated the adoption of healthy eating and PA practices in the childcare sector | | |  | Socio-economic Indexes For Australia | Subgroup: No difference | 2 |
| **Social** | |  | | | | |  |
| Vahid Shahidi et al., 2019 [51] | Social assistance programmes that provide cash benefits | | |  | Employment | Subgroup: No difference | 4 |
| Spence et al., 2010^2^ [2] | Children's Fitness Tax Credit | | |  | Household income | Subgroup: Reduced | Cochrane Public Health Review Group recommended Effective Public Health Practice Project Quality Assessment Tool for Quantitative Studies: Weak |

Table legend

PA: physical activity, PE: physical education, SES: Socioeconomic status, WIC: Special Supplemental Nutrition Program for Women, Infants and Children.

^1^+/reduced inequities: lowest SES group responded more favourably to policy compared to highest SES group (subgroup) *or* low SES group responded favourably to policy (area-level)

=/no difference in inequities: lowest SES group and highest SES group responded similarly to policy (subgroup) *or* low SES group did not respond to policy (area-level)

-/increased inequities: lowest SES group responded less favourably to policy compared to highest SES group (subgroup) *or* low SES group responded negatively to policy (area-level)

^2^ Result extracted from review: Hansmann et al., 2022 [18], Hosford et al., 2021 [17], Hunter et al., 2019 [14], Nickel and von dem Kneseback, 2020 [6], Olstad et al., 2017 [25], Olstad et al., 2016 [2], Smith et al., 2017 [21], and Thomas et al., 2018 [45].

^3^ Summery of quality appraisal tools from primary studies extracted from reviews:

Hunter et al., 2019 [14]: used the tool developed by Twohig-Bennett and Jones (2018). Studies meeting 9 out of 11 criteria were classified as high quality.

Olstad et al., 2017 [25], Olstad et al., 2016 [2], Nickel and von dem Kneseback, 2020 [6], and Smith et al., 2017 [21] applied the *Effective Public Health Practice Project (EPHPP) Quality Assessment Tool for Quantitative Studies*. This tool evaluates selection bias, study design, confounders, data collection methods, and participant withdrawals or dropouts. Studies were rated as weak if they received two or more weak ratings, moderate if they received one weak rating, and strong if no weak ratings were assigned.

**References**

1. Andersen HB, Christiansen LB, Klinker CD, Ersbøll AK, Troelsen J, Kerr J, et al. Increases in Use and Activity Due to Urban Renewal: Effect of a Natural Experiment. Am J Prev Med. 2017;53(3):e81–e7. <https://doi.org/10.1016/j.amepre.2017.03.010>.

2. Olstad DL, Teychenne M, Minaker LM, Taber DR, Raine KD, Nykiforuk CI, et al. Can policy ameliorate socioeconomic inequities in obesity and obesity-related behaviours? A systematic review of the impact of universal policies on adults and children. Obes Rev. 2016;17(12):1198–217. <https://doi.org/10.1111/obr.12457>.

3. Aytur SA, Rodriguez DA, Evenson KR, Catellier DJ, Rosamond WD. The sociodemographics of land use planning: relationships to physical activity, accessibility, and equity. Health Place. 2008;14(3):367–85. <https://doi.org/10.1016/j.healthplace.2007.08.004>.

4. Bijlani C, Vrinten C, Junghans C, Chang K, Lewis E, Mulla U, et al. Changes in diet and physical activity following a community-wide pilot intervention to tackle childhood obesity in a deprived inner-London ward. BMC Public Health. 2024;24(1):800. <https://doi.org/10.1186/s12889-024-18192-8>.

5. Boelens M, Raat H, Jonkman H, Hosman CMH, Wiering D, Jansen W. Effectiveness of the Promising Neighbourhoods community program in 0-to 12-year-olds: A difference-in-difference analysis. SSM Popul Health. 2022;19:101166. <https://doi.org/10.1016/j.ssmph.2022.101166>.

6. Nickel S, von dem Knesebeck O. Do multiple community-based interventions on health promotion tackle health inequalities? INTERNATIONAL JOURNAL FOR EQUITY IN HEALTH. 2020;19(1):157. <https://doi.org/10.1186/s12939-020-01271-8>.

7. Buscemi J, Odoms-Young A, Stolley MR, Schiffer L, Blumstein L, Clark MH, et al. Comparative Effectiveness Trial of an Obesity Prevention Intervention in EFNEP and SNAP-ED: Primary Outcomes. Nutrients. 2019;11(5). <https://doi.org/10.3390/nu11051012>.

8. Cheadle A, Atiedu A, Rauzon S, Schwartz PM, Keene L, Davoudi M, et al. A Community-Level Initiative to Prevent Obesity: Results From Kaiser Permanente's Healthy Eating Active Living Zones Initiative in California. Am J Prev Med. 2018;54(5 Suppl 2):S150–s9. <https://doi.org/10.1016/j.amepre.2018.01.024>.

9. Derose KP, Williams MV, Flórez KR, Griffin BA, Payán DD, Seelam R, et al. Eat, Pray, Move: A Pilot Cluster Randomized Controlled Trial of a Multilevel Church-Based Intervention to Address Obesity Among African Americans and Latinos. Am J Health Promot. 2019;33(4):586–96. <https://doi.org/10.1177/0890117118813333>.

10. Heath GW, Bilderback J. Grow Healthy Together: Effects of Policy and Environmental Interventions on Physical Activity Among Urban Children and Youth. J Phys Act Health. 2019;16(2):172–6. <https://doi.org/10.1123/jpah.2018-0026>.

11. Herens M, Bakker EJ, van Ophem J, Wagemakers A, Koelen M. Health-Related Quality of Life, Self-Efficacy and Enjoyment Keep the Socially Vulnerable Physically Active in Community-Based Physical Activity Programs: A Sequential Cohort Study. PLoS One. 2016;11(2):e0150025. <https://doi.org/10.1371/journal.pone.0150025>.

12. Jalaludin B, Maxwell M, Saddik B, Lobb E, Byun R, Gutierrez R, et al. A pre-and-post study of an urban renewal program in a socially disadvantaged neighbourhood in Sydney, Australia. BMC Public Health. 2012;12:521. <https://doi.org/10.1186/1471-2458-12-521>.

13. Ruijsbroek A, Wong A, den Hertog F, Droomers M, van den Brink C, Kunst AE, et al. Do inhabitants profit from integrating a public health focus in urban renewal programmes? A Dutch case study. PLoS One. 2022;17(6):e0270367. <https://doi.org/10.1371/journal.pone.0270367>.

14. Hunter RF, Cleland C, Cleary A, Droomers M, Wheeler BW, Sinnett D, et al. Environmental, health, wellbeing, social and equity effects of urban green space interventions: A meta-narrative evidence synthesis. Environ Int. 2019;130:104923. <https://doi.org/10.1016/j.envint.2019.104923>.

15. Kramer D, Droomers M, Jongeneel-Grimen B, Wingen M, Stronks K, Kunst AE. The impact of area-based initiatives on physical activity trends in deprived areas; a quasi-experimental evaluation of the Dutch District Approach. INTERNATIONAL JOURNAL OF BEHAVIORAL NUTRITION AND PHYSICAL ACTIVITY. 2014;11:36. <https://doi.org/10.1186/1479-5868-11-36>.

16. Adams EJ, Cavill N. Engaging communities in changing the environment to promote transport-related walking: Evaluation of route use in the 'Fitter for Walking' project. Journal of Transport and Health. 2015;2(4):580–94. <https://doi.org/10.1016/j.jth.2015.09.002>.

17. Hosford K, Firth C, Brauer M, Winters M. The effects of road pricing on transportation and health equity: A scoping review. Transp Rev. 2021;41:766–87. <https://doi.org/10.1080/01441647.2021.1898488>.

18. Hansmann KJ, Grabow M, McAndrews C. Health equity and active transportation: A scoping review of active transportation interventions and their impacts on health equity. Journal of Transport and Health. 2022;25:101346. <https://doi.org/10.1016/j.jth.2022.101346>.

19. Cook T, O’Brien S, Jackson K, Findley D, Searcy S. Behavioral Effects of Completing a Critical Link in the American Tobacco Trail. Transportation Research Record: Journal of the Transportation Research Board. 2016;2598:19–26. <https://doi.org/10.3141/2598-03>.

20. Goodman A, Cheshire J. PP70 Inequalities in the London bicycle sharing system revisited: impacts of extending the scheme to poorer areas but then doubling prices. Journal of Transport Geography. 2014;41. <https://doi.org/10.1016/j.jtrangeo.2014.04.004>.

21. Smith M, Hosking J, Woodward A, Witten K, MacMillan A, Field A, et al. Systematic literature review of built environment effects on physical activity and active transport - an update and new findings on health equity. Int J Behav Nutr Phys Act. 2017;14(1):158. <https://doi.org/10.1186/s12966-017-0613-9>.

22. Iroz-Elardo N, Schoner J, Fox EH, Brookes A, Frank LD. Active travel and social justice: Addressing disparities and promoting health equity through a novel approach to Regional Transportation Planning. Soc Sci Med. 2020;261:113211. <https://doi.org/10.1016/j.socscimed.2020.113211>.

23. Martin A, Morciano M, Suhrcke M. Determinants of bicycle commuting and the effect of bicycle infrastructure investment in London: Evidence from UK census microdata. Econ Hum Biol. 2021;41:100945. <https://doi.org/10.1016/j.ehb.2020.100945>.

24. Brownson RC, Housemann RA, Brown DR, Jackson-Thompson J, King AC, Malone BR, et al. Promoting physical activity in rural communities: walking trail access, use, and effects. Am J Prev Med. 2000;18(3):235–41. <https://doi.org/10.1016/s0749-3797(99)00165-8>.

25. Olstad DL, Ancilotto R, Teychenne M, Minaker LM, Taber DR, Raine KD, et al. Can targeted policies reduce obesity and improve obesity-related behaviours in socioeconomically disadvantaged populations? A systematic review. Obes Rev. 2017;18(7):791–807. <https://doi.org/10.1111/obr.12546>.

26. Dulin-Keita A, Clay O, Whittaker S, Hannon L, Adams IK, Rogers M, et al. The influence of HOPE VI neighborhood revitalization on neighborhood-based physical activity: A mixed-methods approach. Soc Sci Med. 2015;139:90–9. <https://doi.org/10.1016/j.socscimed.2015.06.002>.

27. Kodali HP, Wyka KE, Costa SA, Evenson KR, Thorpe LE, Huang TT. Association of Park Renovation With Park Use in New York City. JAMA Netw Open. 2024;7(4):e241429. <https://doi.org/10.1001/jamanetworkopen.2024.1429>.

28. Lopes F, Figueiredo L, Gil J, Trigueiro E. Evaluating the impact of social housing policies: Measuring accessibility changes when individuals move to social housing projects. Environment and Planning B: Urban Analytics and City Science. 2023. <https://doi.org/10.1177/23998083231218774>.

29. Zenk SN, Pugach O, Ragonese-Barnes M, Odoms-Young A, Powell LM, Slater SJ. Did Playground Renovations Equitably Benefit Neighborhoods in Chicago? J Urban Health. 2021;98(2):248–58. <https://doi.org/10.1007/s11524-020-00472-4>.

30. Barbosa Filho VC, Bandeira ADS, Minatto G, Linard JG, Silva JAD, Costa RMD, et al. Effect of a Multicomponent Intervention on Lifestyle Factors among Brazilian Adolescents from Low Human Development Index Areas: A Cluster-Randomized Controlled Trial. Int J Environ Res Public Health. 2019;16(2). <https://doi.org/10.3390/ijerph16020267>.

31. Carson RL, Castelli DM, Pulling Kuhn AC, Moore JB, Beets MW, Beighle A, et al. Impact of trained champions of comprehensive school physical activity programs on school physical activity offerings, youth physical activity and sedentary behaviors. Prev Med. 2014;69 Suppl 1:S12–9. <https://doi.org/10.1016/j.ypmed.2014.08.025>.

32. Mendoza JA, Levinger DD, Johnston BD. Pilot evaluation of a walking school bus program in a low-income, urban community. BMC Public Health. 2009;9:122. <https://doi.org/10.1186/1471-2458-9-122>.

33. Nathan N, Wolfenden L, Williams CM, Yoong SL, Lecathelinais C, Bell AC, et al. Adoption of obesity prevention policies and practices by Australian primary schools: 2006 to 2013. Health Educ Res. 2015;30(2):262–71. <https://doi.org/10.1093/her/cyu068>.

34. Sutherland RL, Campbell EM, Lubans DR, Morgan PJ, Nathan NK, Wolfenden L, et al. The Physical Activity 4 Everyone Cluster Randomized Trial: 2-Year Outcomes of a School Physical Activity Intervention Among Adolescents. Am J Prev Med. 2016;51(2):195–205. <https://doi.org/10.1016/j.amepre.2016.02.020>.

35. Vander Ploeg KA, McGavock J, Maximova K, Veugelers PJ. School-based health promotion and physical activity during and after school hours. Pediatrics. 2014;133(2):e371–8. <https://doi.org/10.1542/peds.2013-2383>.

36. Andrade ACS, Mingoti SA, Fernandes AP, Andrade RG, Friche AAL, Xavier CC, et al. Neighborhood-based physical activity differences: Evaluation of the effect of health promotion program. PLoS One. 2018;13(2):e0192115. <https://doi.org/10.1371/journal.pone.0192115>.

37. Candio P, Meads D, Hill AJ, Bojke L. Cost-effectiveness of a proportionate universal offer of free exercise: Leeds Let's Get Active. J Public Health (Oxf). 2021;43(4):876–86. <https://doi.org/10.1093/pubmed/fdaa113>.

38. Higgerson J, Halliday E, Ortiz-Nunez A, Barr B. The impact of free access to swimming pools on children’s participation in swimming. A comparative regression discontinuity study. Journal of Public Health. 2018;41(2):214–21. <https://doi.org/10.1093/pubmed/fdy079>.

39. Hoekman R, Breedveld K, Kraaykamp G. Providing for the rich? The effect of public investments in sport on sport (club) participation of vulnerable youth and adults. European Journal for Sport and Society. 2017;14(4):327–47. <https://doi.org/10.1080/16138171.2017.1421510>.

40. Rabiee F, Robbins A, Khan M. Gym for Free: The short-term impact of an innovative public health policy on the health and wellbeing of residents in a deprived constituency in Birmingham, UK. Health Education Journal. 2015;74(6):691–704. <https://doi.org/10.1177/0017896914553957>.

41. Reilly K, Bauman A, Reece L, Lecathelinais C, Sutherland R, Wolfenden L. Evaluation of a voucher scheme to increase child physical activity in participants of a school physical activity trial in the Hunter region of Australia. BMC Public Health. 2021;21(1):570. <https://doi.org/10.1186/s12889-021-10588-0>.

42. Taylor P, Panagouleas T, Kung SP. Access to English public sector sports facilities by disadvantaged groups and the effect of financial objectives. Managing Leisure. 2011;16(2):128–41. <https://doi.org/10.1080/13606719.2011.559091>.

43. Virmasalo I, Hasanen E, Pyykönen J, Nurmi M, Simula M, Salmikangas AK, et al. Closed due to COVID-19: effects of indoor sports restrictions on suburban adults’ physical activity behaviours. International Journal of Sport Policy and Politics. 2023;15(2):249–69. <https://doi.org/10.1080/19406940.2023.2178479>.

44. Williams O. Identifying adverse effects of area-based health policy: An ethnographic study of a deprived neighbourhood in England. Health Place. 2017;45:85–91. <https://doi.org/10.1016/j.healthplace.2017.02.011>.

45. Thomas M, Phongsavan P, McGill B, O'Hara B, Bauman A. A review of the impact of physical activity mass media campaigns on low compared to high socioeconomic groups. Health education research. 2018;33. <https://doi.org/10.1093/her/cyy032>.

46. Pena-Y-Lillo M, Lee CJ. A Communication Inequalities Approach to Disparities in Physical Activities: The Case of the VERB Campaign. JOURNAL OF HEALTH COMMUNICATION. 2019;24(2):111–20. <https://doi.org/10.1080/10810730.2019.1583699>.

47. Tomioka M, Braun KL, Compton M, Tanoue L. Adapting Stanford's Chronic Disease Self-Management Program to Hawaii's multicultural population. Gerontologist. 2012;52(1):121–32. <https://doi.org/10.1093/geront/gnr054>.

48. Esquivel M, Nigg CR, Fialkowski MK, Braun KL, Li F, Novotny R. Head Start Wellness Policy Intervention in Hawaii: A Project of the Children's Healthy Living Program. Child Obes. 2016;12(1):26–32. <https://doi.org/10.1089/chi.2015.0071>.

49. Tomayko EJ, Prince RJ, Hoiting J, Braun A, LaRowe TL, Adams AK. Evaluation of a multi-year policy-focused intervention to increase physical activity and related behaviors in lower-resourced early care and education settings: Active Early 2.0. Prev Med Rep. 2017;8:93–100. <https://doi.org/10.1016/j.pmedr.2017.08.008>.

50. Yoong SL, Finch M, Nathan N, Wiggers J, Lecathelinais C, Jones J, et al. A longitudinal study assessing childcare services' adoption of obesity prevention policies and practices. J Paediatr Child Health. 2016;52(7):765–70. <https://doi.org/10.1111/jpc.13252>.

51. Vahid Shahidi F, Sod-Erdene O, Ramraj C, Hildebrand V, Siddiqi A. Government social assistance programmes are failing to protect the health of low-income populations: evidence from the USA and Canada (2003-2014). J Epidemiol Community Health. 2019;73(3):198–205. <https://doi.org/10.1136/jech-2018-211351>.
