# Supplementary material for: The impact of public policy on socioeconomic equity in physical activity: a systematic review
Source: Int J Behav Nutr Phys Act. 2026 Feb 4;23:20. doi: 10.1186/s12966-026-01880-6 (PMC12964968; doi:10.1186/s12966-026-01880-6)
Supplement: Supplementary file 6 — Additional file 6. Overview of study results. [file 12966_2026_1880_MOESM6_ESM.docx]

Additional file 6: Overview of study results

| **No.** | **Author, year, country** | **Public policy intervention** | **Equity aspect** | **Outcome: PA behaviour or PA environment** | **Overall effect on PA** | **Impact on inequities** | **Summary results^2^** |  |
| --- | --- | --- | --- | --- | --- | --- | --- | --- |
| **Community wide domain** | | | | | | | |  |
| 1 | Andersen et al., 2017, DK [1] | A multicomponent urban renewal project of approximately 35 million Euros in a disadvantaged neighborhood in the capital of Denmark occurred between 2010 and 2012. The urban renewal was a multicomponent strategy including, for example, renovation of public housing and courtyards; adding streetlights; renovation or establishment of new urban green spaces, playgrounds, and sport facilities; and opening of two civic centers offering social gatherings and sport activities. Participants: adolescents aged 11–16 years. | Population: Disadvantaged district | PA behaviour - Time spent in MVPA | NA | Reduced inequities | Time spent in the area was greater in 2012 than 2010 with an additional 24.6 minutes per day (p=0.017). Of this time, 7.8 minutes were spent in light and 4.5 minutes in MVPA. Table 2 - both sig.  No comparison group. |  |
| 1 | Andersen et al., 2017, DK [1] |  | Population: Disadvantaged district | PA behaviour - Time spent in light PA | NA | Reduced inequities |  |  |
| 2 | Aytur et al., 2007^1^, US [2] | North Carolina counties’ land use and transportation plans. Participants: adults | Overall population: 67 counties in North Carolina Subgroup measure: Household income | PA behaviour - Transportation-related PA | Not available in review | No difference | Review: Neutral (The lowest and highest SEP groups responded similarly to the policy)  Primary study: no adaptation |  |
| 3 | Aytur et al., 2008, US [3] | Counties’ land use plans that included non-automobile transportation improvements and more comprehensive policies to guide development. Participants: adults | Overall population: Generally representative sample of the state (sample had higher median income) Subgroup measure: Income | PA behaviour - Transportation-related PA | Increase | Reduced inequities | Overall effects: The coefficients for more comprehensive implementation tool sets showed signs in the positive direction across the spectrum of PA outcomes. Stronger associations were observed for the most comprehensive tool sets (sig.), especially with respect to transportation-related PA and bicycling.  Health equity effects: Because lower-income individuals are more likely to walk or bicycle for transportation, we further examined relationships between transportation related PA and the comprehensiveness of implementation tools, stratified by household income level (Figure 1). Among lower-income individuals (household income <$25,000), the prevalence of transportation-related PA for residents of counties with the most comprehensive implementation tool sets was three times higher (30%) compared with residents of counties with the least comprehensive tool sets (11%). Among adults with higher household incomes (>$25,000), the prevalence of transportation-related PA did not differ appreciably across categories of comprehensiveness.  % walking or bicycling for transportation high-income households with most comprehensive tools: 17% *- no test for sig.* |  |
| 4 | Bijlani et al., 2024, UK [4] | The Go-Golborne intervention aimed to shape the local environment in the Golborne ward (deprived inner-Londen ward) across multiple settings  with the engagement of a large number of local government and community stakeholders in a joint approach. Activities focused on six co-created themes to make changes to local environments and reduce sugary snacks and beverage consumption, increase fruit and vegetable intake, promote healthy snacks, increase active play and travel, and reduce screen time. Participants: children aged 6-11 years. | Overall population: The second most deprived ward in London Subgroup measure: Deprivation | PA behaviour - School commute | No effect | No difference | Overall effects (disadvantaged population): After three years of follow-up, there were reductions in sugar-sweetened beverage consumption (adjusted beta -0·43 occasions/day, 95% CI -0·55 to -0·32), fruit and vegetable consumption (adjusted beta -0.22 portions, 95% CI -0.44 to 0.001) and car travel to and from school (adjusted OR 0·19, 95% CI 0·06 to 0·66), while screen time increased (high versus moderate/low: OR 2·30, 95% CI 1·36 to 3·90). For other behavioural outcomes, there was no statistically sig. evidence of changes.  No control group.  Health equity effects: Tests of interaction terms show no difference in changes of study outcomes between subgroups of gender, ethnicity, weight status, or deprivation, except for a significantly lower consumption of sugary snacks identified among the least derived (quintile 3–5) at 2-year follow-up (-0.48 occasions/day; 95% CI: -0.91, -0.05). |  |
| 4 | Bijlani et al., 2024, UK [4] |  | Overall population: The second most deprived ward in London Subgroup measure: Deprivation | PA behaviour - Active play | No effect | No difference |  |  |
| 5 | Boelens et al., 2022, NL [5] | The Promising Neighbourhoods program was developed by the municipality of Rotterdam with the aim to increase the health, safety and talent development of youth living  in Rotterdam, and contribute to the reduction of socioeconomic inequalities. The program is a collaborative community-based approach that includes community stakeholders, works with data-driven priority setting, knowledge- and theory-based policies and focuses on implementation of evidence-based interventions. The program is managed by municipal district advisors. Together with community stakeholders and key-leaders from the neighbourhood  network the municipal district advisor plans and develops a tailored intervention package for the neighbourhood. Participants: 0-12 years old. | Overall population: Random sample municipal population register Subgroup measure: SES | PA behaviour - Sport club membership (4-12 years old) | No effect | No difference | Overall effects: Difference-in-difference analysis: There is an intervention effect of the Promising Neighbourhoods program on outdoor-play. No other intervention effects on the outcomes were found (Model 1).  Health equity effects: There were no sig. different intervention effects for children with a lower or higher SES on the outcomes. |  |
| 5 | Boelens et al., 2022, NL [5] |  | Overall population: Random sample municipal population register Subgroup measure: SES | PA behaviour - Outdoor play (0-12 years old) | Increase | No difference |  |  |
| 6 | Buscail et al., 2016^1^, FR [6] | 1) Social marketing (flyers and informative brochures on physical activity)  3) Networking/partnership (questioning residents on barriers)  4) Environmental change (offering and access to physical activity at community centers; pedestrian orientation paths)  Participants: adults | Overall population: Socially disadvantaged community (low-income neighborhood)  Subgroup measure: professional status | PA behaviour – World Health Organization guidelines | Increase | No difference | Review: Reduced inequalities (area level)  Primary study:  Area level: no adaptation  Increased leisure, global, and walking scores; no effect on regular sport participation or work-related physical activity.  Subgroup level: adaptation required  None of the other explored interactions (e.g. professional status) were significant. |  |
| 6 | Buscail et al., 2016^1^, FR [6] |  | Overall population: Socially disadvantaged community (low-income neighborhood)  Subgroup measure: professional status | PA behaviour – leisure score | Increase | No difference |  |  |
| 6 | Buscail et al., 2016^1^, FR [6] |  | Overall population: Socially disadvantaged community (low-income neighborhood)  Subgroup measure: professional status | PA behaviour – work score | No effect | No difference |  |  |
| 6 | Buscail et al., 2016^1^, FR [6] |  | Overall population: Socially disadvantaged community (low-income neighborhood)  Subgroup measure: professional status | PA behaviour – walking | Increase | No difference |  |  |
| 6 | Buscail et al., 2016^1^, FR [6] |  | Overall population: Socially disadvantaged community (low-income neighborhood)  Subgroup measure: professional status | PA behaviour – Regularly practicing sport | No effect | No difference |  |  |
| 7 | Buscemi et al., 2019, US [7] | Hip-Hop to Health (HH): Evidence-based obesity prevention intervention, delivered through Expanded Food and Nutrition Education Program (EFNEP) and the Supplemental Nutrition Assistance Program-Education (SNAP-Ed). HH is a school-based nutrition and physical activity obesityprevention program for low-income preschool children. Parent–child dyads (one parent/child per household) in HH received an eight-lesson obesity prevention intervention that was delivered over 6–8 weeks. Participants: parent–child dyads. | Population: Low-income parents and children | PA behaviour - Godin leisure time exercise, moderate and strenuous only (parents) | NA | No difference | No differences in child or caregiver diet, PA, or screen time by group were found. | |
| 7 | Buscemi et al., 2019, US [7] |  | Population: Low-income parents and children | PA behaviour - Godin leisure time exercise, moderate and strenuous only (children) | NA | No difference |  |  |
| 7 | Buscemi et al., 2019, US [7] |  | Population: Low-income parents and children | PA behaviour - Godin leisure time exercise (parents) | NA | No difference |  |  |
| 7 | Buscemi et al., 2019, US [7] |  | Population: Low-income parents and children | PA behaviour - Godin leisure time exercise (children) | NA | No difference |  |  |
| 8 | Cheadle et al., 2018, US [8] | The Healthy Eating Active Living Zones design targeted places and people through policy, environmental, and programmatic strategies. Each Healthy Eating Active Living Zone is a small, low-income community of 10,000 to 20,000 residents with high obesity rates and other health disparities. Community coalitions planned and implemented strategies in each community.  The article focused on two regions: Northern California and Southern California (design and evaluations were closely aligned). Participants: youth and adults. **Five of the six HEAL Zones added trails and new equipment to parks. Some sites began promoting the new equipment/trails through exercise programs.** | Population: Low-income communities | PA behaviour - Intensity of exercise | NA | No difference | No overall increase in intensity of exercise among park users.  No control  *- no test for sig.* |  |
| 9 | Derose et al., 2019, US [9] | Multi-level church based intervention to address obesity. Various components were implemented over 5 months and included 2 sermons by pastor, educational handouts, church vegetable and fruit gardens, cooking and nutrition classes, daily mobile messaging, community mapping of food and physical activity environments, and identification of congregational policy changes to increase healthy meals. Study area: LA County Service Planning Area 6, disadvantaged residents in terms of socioeconomic status. South LA offers an important setting in which to address health disparities [...]  Participants: adult congregants | Population: Disadvantaged in terms of SES (e.g., income, education, wealth, employment) | PA behaviour - Minutes spent in PA each week | NA | No difference | Among those completing follow-up (68%), the intervention resulted in statistically significantly less weight gain and greater weight loss (-0.05 effect sizes; 95% CI [CI] = -0.06 to -0.04), lower body mass index (-0.08; 95% CI = -0.11 to -0.05), and healthier diet (-0.09; 95% CI = -0.17 to -0.00). There was no evidence of an intervention impact on PA minutes per week. |  |
| 10 | Goodman et al., 2013a^1^, UK [6] | 2) Individual or group education (e.g. cycle training in schools and colleges)  4) Environmental change (e.g. cycle lanes, cycle parking stands at workplaces)  Participants: commuters | Overall population: Socially disadvantaged community Subgroup measure: Deprived area | PA behaviour - Walking to work | Increase | Decreased inequities | Review: Reduced inequalities at area level, but smaller in the most of the deprived areas  Primary study:  Area level: no adaptation  Cycling: Compared with the matched comparison group, this represented an absolute intervention effect of +0.69 (95% CI 0.60, 0.77) percentage points  Walking: the difference-in-differences for the intervention vs. matched comparison group was +0.73 (95% CI 0.59, 0.87) percentage points  Public transport: By contrast, the small increase in using public transport was similar to that observed in the comparison groups (e.g. difference-in-differences 0.04 (−0.09, 0.17) percentage points for intervention vs. matched comparison group  Subgroup level: adaptation required  Cycling: Cycling in the intervention towns increased significantly among commuters living in all fifths of small-area deprivation  Walking: The prevalence of walking in the intervention towns increased across all fifths of deprivation, and increased by more than any of the comparison groups (although in the most affluent fifth this difference was not significant)  Public transport: The change in public transport use across deprivation fifths was generally similar between all groups, |  |
| 10 | Goodman et al., 2013a^1^, UK [6] |  | Overall population: Socially disadvantaged community Subgroup measure: Deprived area | PA behaviour - Cycling to work | Increase | No difference |  |  |
| 10 | Goodman et al., 2013a^1^, UK [6] |  | Overall population: Socially disadvantaged community Subgroup measure: Deprived area | PA behaviour – Public transport to work | No effect | No difference |  |  |
| 11 | Heath and Bilderback, 2019, US [10] | Grow Healthy Together Chattanooga: implementing evidence-based strategies for promoting child/youth health and reduce childhood obesity in east and south Chattanooga, home to residents identified as the most vulnerable to overweight and obesity. The goal of increasing access to places for physical activity was achieved by increasing the number of acres of green space per 1000 residents through increased numbers of parks/recreational sites, length of added sidewalks in each community, and length of designated bicycle lanes in each community (i.e., new parks, after school open use).  Participants: children/youth | Population: Economically depressed areas | PA behaviour - Observed level of VPA | NA | Increased inequities | Table 3: Sig. increase in walking and MVPA. Sig. decrease in VPA. No control group. |  |
| 11 | Heath and Bilderback, 2019, US [10] |  | Population: Economically depressed areas | PA behaviour - Observed level of walking | NA | Reduced inequities |  |  |
| 11 | Heath and Bilderback, 2019, US [10] |  | Population: Economically depressed areas | PA behaviour – Observed level of MVPA | NA | Reduced inequities |  |  |
| 12 | Herens et al., 2016, NL [11] | Community-based health enhancing physical activity (CBHEPA) programs, summarised under the denominator ‘Communities on the Move’ (CoM). CoM was developed and disseminated by the Netherlands Institute for Sports and PA (NISB) from 2003 to 2012. CoM targets inactive, socially vulnerable groups with the aim of enhancing PA levels, hence contributing to participants’ health-related quality of life.  Participants from 19 groups (10–20 participants) in on-going CBHEPA programs targeting socially vulnerable groups in seven different municipalities. | Overall population: Socially vulnerable groups (low-income, low-education) Subgroup measure: Education | PA behaviour - leisure-time PA | No effect | Increased inequities | Overall effects (disadvantaged population): We did not find evidence to confirm the hypothesis (Hypothesis 3) that participation in a community-based health-enhancing PA program leads to an increase in its participants’ leisure-time PA levels over time.  No sig. differences were found between the ‘active’ and ‘control group by proxy’ for log transformed leisure-time PA (LOG LTPA) (t(11) 1.14, p = 0.28) and (log transformed) total PA (t(11) -0.57, p = 0.58).  Health equity effects: Findings relating to the full model (model 8) for differences in educational level suggested that LOG LTPA was significantly higher (p<0.050) among participants with higher educational levels, but that there was no sig. difference in educational level between participants and program dropouts. |  |
| 13 | Higgerson et al., 2018b^1^, UK [6] | 1) Social marketing (considerable promotional activities to raise awareness)  2) Individual or group education (full time equivalent health trainers)  3) Networking/partnership (Healthy Communities Partnership)  4) Environmental change (free access to leisure facilities) | Overall population: Areas of deprivation in the UK  Subgroup measure: Socio-economic group | PA behaviour – participating in gym or swim activity at least once in the past month | Increase | No difference | Review results: Slightly reduced inequalities (area level), greater in the most disadvantaged subgroup.  Primary study results:  Area level: no adaptation  The intervention was associated with an additional 3.9% of the population in Blackburn with Darwen participating in at least 30 min of moderate-intensity gym or swim sessions during the last four weeks (95% CI 3.6 to 4.1).  In terms of overall participation in physical activity, the intervention was associated with an additional 1.9% of the population participating in any sport or active recreation of at least moderate intensity for at least 30 min on at least 12 days out of the last four weeks (95% CI 1.7 to 2.1).  Subgroup level: adaptation required  Fig 3  Participating in gym or swim activity at least once in the past month: all subgroups significant  Participating in any sport or active recreation for a least 30 min on at least 12 days over the last four weeks: This effect was much larger in the more disadvantaged routine and manual group (3.6%, 95% CI 3.3 to 3.8) and was not significant in the more advantaged socioeconomic groups. |  |
|  |  |  | Overall population: Areas of deprivation in the UK  Subgroup measure: Socio-economic group | PA-behaviour - participating in any sport or active recreation for a least 30 min on at least 12 days over the last four weeks | Increase | Reduced inequities |  |  |
| 14 | Jalaludin et al., 2012, AUS [12] | The urban renewal program was conducted in a socially disadvantaged neighbourhood, over 16 months and consisted of internal upgrades (including internal painting; replacement of kitchens, bathrooms and carpets; general maintenance), external upgrades (including property painting; new fencing, carports, letterboxes, concrete driveways, drainage and landscaping), general external maintenance, and social interventions such as community engagement activities, employment initiatives, and building a community meeting place. Setting: conducted in a social housing neighbourhood in south-Western Sydney.  Participants: Adult population | Population: Socially disadvantaged community | PA behaviour - Adequate PA | NA | No difference | There were no sig. differences in the proportion of daily smoking, hazardous alcohol intake, adequate PA, and overweight/obesity before and after the urban renewal program.  No control group. |  |
| 15 | Mohan et al., 2017^1^, UK [6] | 2) Individual or group education (e.g. employability and educational courses)  3) Networking/partnership (e.g. 3-years-action plan by local stakeholders and residents)  4) Environmental change (e.g. housing quality, land developed for green space)  5) Regulatory interventions (e.g. traffic calming schemes, security measures)  6) Sense of community (social neighborhood environment) | Overall population: Socially disadvantaged community (deprived areas)  Subgroup measure: education, employment | PA behaviour - Exercise | No effect | No difference | Review: No difference or only small trend towards a reduction in inequalities (area level)  Primary study:  Area level: no adaptation  The percentage of respondents undertaking weekly exercise in NRAs increased from 56% in wave 2 to 70% in wave 12, while control groups did not experience a corresponding upsurge. However, modelling indicates that the changes between NRAs and non-NRAs over the decade were not statistically significant.  Subgroup level: adaptation required  No sig. interaction by education and employment. |  |
| 16 | O’Loughlin et al., 1999^1^, CA [6] | 1) Social marketing (e.g. nutrition campaign, menu-labeling in local restaurants, contests)  2) Individual or group education (e.g. smoking-cessation and nutrition workshops, screening for CVD risk). | Population: Socially disadvantaged community (low-income, innercity neighborhood)  Subgroup: Education | PA behaviour - level of PA | No effect | No difference | Review: No difference or only small increase in frequency of cholesterol checkups (area level)  Primary study:  Area level: no adaptation  Subgroup level: adaptation required  None of the other subgroup analyses provide evidence for a program effect |  |
| 17 | Phillips et al., 2014^1^, UK [6] | 2) Individual or group education (e.g. physical activity sessions, healthy cooking classes)  3) Networking/partnership (e.g. partnerships with local and city-wide organizations)  4) Environmental change (e.g. community gardens and redevelopment of greenspaces, availability of healthy food)  6) Sense of community (intercultural and intergenerational approaches) | Overall population: Socially disadvantaged community (deprived neighborhoods)  Subgroup measure: Education, employment | PA behaviour - Not specified | No effect | No difference | Review: No difference (area level)  Primary study:  Area level: no adaptation  There was no evidence of impact on physical activity (RR:1.01, 95% CI 0.88 to 1.16). Also not for secondary PA outcomes.  Subgroup level: adaptation required  There was no indication of any differential effects in subgroups defined by age, gender, ethnicity, educational attainment or employment status. |  |
| 18 | Raine et al., 2013^1^, CA [6] | 2) Individual or group education (e.g. leisure activities to encourage people to be active)  3) Networking/partnership (e.g. regular tele-conferences, team meetings)  4) Environmental change (e.g. walking and cycling trails, community gardens)  5) Regulatory interventions (e.g. food security initiatives)  6) Sense of community (promote social inclusion) | Population: Socially disadvantaged community (socioeconomically diverse areas) | PA behaviour - Not specified | NA | Increased inequities | Review: No difference in health outcomes (area level)  Primary study: adaptation required  Follow-up assessment revealed a drop in physical activity levels among HAC communities while Alberta secular trends reported increased physical activity (–0.05; p b 0.001). |  |
| 19 | Ruijsbroek et al., 2022, NL [13] | The Healthy District Experiments (HDE) was implemented in 19 of the 40 target districts of the Dutch District Approach (13 municipalities) and comprised of activities to improve the health of the local population, such as creating small scale sport fields and playgrounds or providing health information in foreign languages. These activities were an addition to the activities on the five policy themes of the District Approach (i.e. employment, education, housing and the physical environment, safety, and social cohesion). The aim of the District Approach was to improve living conditions of the 40 most deprived districts of the Netherlands. The implementation of the HDE started between 2009 and 2011. Even though the initial aim was to improve the health in the HDE districts in a period of ten years, the experiments stopped in February 2014.  Data comprised the adult population (18+) only. | Population: Deprived areas | PA behaviour - Sport participation | NA | No difference | In the Healthy District Experiments target districts, leisure time cycling increased between the pre-intervention and late intervention period (41.9% and 45.8% respectively), but this was not a statistically sig. change. The other outcomes either remained stable over time (mental health, smoking, sport participation) or worsened (general health, overweight, obesity, leisure time walking) between the pre-intervention and late intervention period in the Healthy District Experiments target districts. The change in obesity was borderline sig. (p-value 0.05), the other changes were not statistically sig.. Table 3 difference-in-difference analyses: no sig. results |  |
| 19 | Ruijsbroek et al., 2022, NL [13] |  | Population: Deprived areas | PA behaviour - Leisure-time cycling | NA | No difference |  |  |
| 19 | Ruijsbroek et al., 2022, NL [13] |  | Population: Deprived areas | PA behaviour - Leisure-time walking | NA | No difference |  |  |
| 20 | Schulz et al., 2015^1^, US [6] | 2) Individual or group education (training and support lay health promoters, walking group)  3) Networking/partnership (long-standing collaboration among community groups, health service providers, and researchers)  4) Environmental change (e.g. improvements to parks and greenways, safety environment) | Population: Socially disadvantaged community (low-to-moderate income area) | PA behaviour - Not specified | NA | Reduced inequities | Review: Reduced inequalities (area level), no difference by SES  Area level: no adaptation  Subgroup: adaptation required.  No subgroup results available |  |
| 21 | Tester and Baker, 2009^1^, US [14] | Major renovations to 2 parks: lighting, fencing, artificial turf, landscaping, picnic benches, goal posts, walkways and a park initiative to improve family and youth involvement (e.g. expanded hours of park operation (e.g. playfield lights kept on during later evening hours), professional training and skills development for park and recreation program staff, and expanded programs driven by community input (e.g. dances organized by teens for teens). | Population: Resource poor neighbourhoods, median household income $34–56,000 | PA behaviour - SOPARC park use | NA | Reduced inequities | Review: Sig increases of >4-fold magnitude among children and adults of both genders at the intervention park playfields, but not in the control park; Sig park use in non-play fields.  significantly positive post-intervention effect for increasing park usage and PA  Primary study: adaptation required  Table 3  Males: no effect moderate PA  Increase vigorous PA  Females: increase moderate PA  Increase vigorous PA |  |
| 21 | Tester and Baker, 2009^1^, US [14] |  | Population: Resource poor neighbourhoods, median household income $34–56,000 | PA behaviour - Vigorous PA females | NA | Reduced inequities |  |  |
| 21 | Tester and Baker, 2009^1^, US [14] |  | Population: Resource poor neighbourhoods, median household income $34–56,000 | PA behaviour -Moderate PA females | NA | Reduced inequities |  |  |
| 21 | Tester and Baker, 2009^1^, US [14] |  | Population: Resource poor neighbourhoods, median household income $34–56,000 | PA behaviour -Moderate PA males | NA | No difference |  |  |
| 21 | Tester and Baker, 2009^1^, US [14] |  | Population: Resource poor neighbourhoods, median household income $34–56,000 | PA behaviour - Vigorous PA males | NA | Reduced inequities |  |  |
| 22 | Kramer et al., 2014, NL [15] | The District Approach aims to alleviate problems of employment, education, housing and the physical environment, safety, and social integration in 40 of the most deprived districts of the Netherlands. Districts have been selected based on their accumulation of economic, physical, and social problems, judged on statistics and survey data. Each district developed its own mix of socio-economic and environmental interventions (housing quality, neighbourhood regeneration, green space, footpaths and cycle tracks, play grounds, sports facilities and activities, social capital, nuisance and conflicts, nuisance from youth, physical disorder, burglary, traffic safety). Participants: adults. | Population: Deprived districts | PA behaviour - Walking | NA | Reduced inequities | Deprived target districts showed a significantly positive change in walking trend between the pre-intervention and intervention period. The trend change in the deprived target districts was significantly larger compared to the rest of the Netherlands, but not compared to other deprived districts. For cycling and sports, neither deprived districts nor control districts showed a sig. trend change.  Control groups in study: Rest of the Netherlands (main control group), Other deprived districts (same city) | |
| 22 | Kramer et al., 2014, NL [15] |  | Population: Deprived districts | PA behaviour - Sports | NA | No difference |  |  |
| 22 | Kramer et al., 2014, NL [15] |  | Population: Deprived districts | PA behaviour - Cycling | NA | No difference |  |  |
| **Transport domain** | | | | | | | | |
| 23 | Adams and Cavill, 2015, UK [16] | The Fitter for Walking (FFW) project was launched in 2008 and completed in early 2012. The main aims of the FFW project were to: (1) improve the local neighbourhood walking environment; (2) increase the number of people walking on a specific route targeted for environmental improvements; and (3) encourage communities and local residents to work together to promote walking. Twelve local authority (LA) partners from five regions of England were recruited by Living Streets to take part in the project. The LAs were recruited on the basis of having low levels of reported physical activity [...] and being based in an area of high deprivation. Activities were delivered across three areas: (1) local authority-led infrastructural changes e.g. new street lighting, dropped curbs, removal of street furniture such as bollards or railings; (2) community-led environmental changes e.g. bulb planting, street cleaning; and (3) promotional and awareness raising activities e.g. led walks to increase awareness of a newly improved route. Participants: minor 0–15; adult 16–59 or older adult 60+. | Population: Deprived communities in twelve local authority areas in England | PA behaviour - Meeting PA recommendations | NA | Reduced inequities | Table 3: T1-T2 sig. increase PA  Table 4: Pedestrian route use (no test for sig.) T1-T3  Table 5: Other outcomes (based on sig.) T1-T2  Main outcome: route use  No control group. | |
| 23 | Adams and Cavill, 2015, UK [16] |  | Population: Deprived communities in twelve local authority areas in England | PA behaviour - Perceived change in route use in last 12 months | NA | No difference |  |  |
| 23 | Adams and Cavill, 2015, UK [16] |  | Population: Deprived communities in twelve local authority areas in England | PA behaviour - % Use route on a daily-weekly basis at night | NA | Increased inequities |  |  |
| 23 | Adams and Cavill, 2015, UK [16] |  | Population: Deprived communities in twelve local authority areas in England | PA behaviour - % Use route on a daily-weekly basis during the day | NA | Increased inequities |  |  |
| 23 | Adams and Cavill, 2015, UK [16] |  | Population: Deprived communities in twelve local authority areas in England | PA behaviour - Time spent walking on journey (minutes spent walking) | NA | No difference |  |  |
| 23 | Adams and Cavill, 2015, UK [16] |  | Population: Deprived communities in twelve local authority areas in England | PA behaviour - Use of other modes of travel that promote PA by pedestrian route users | NA | No difference |  |  |
| 23 | Adams and Cavill, 2015, UK [16] |  | Population: Deprived communities in twelve local authority areas in England | PA behaviour - Pedestrian route use week day | NA | Reduced inequities |  |  |
| 23 | Adams and Cavill, 2015, UK [16] |  | Population: Deprived communities in twelve local authority areas in England | PA behaviour - Pedestrian route use weekend | NA | Increased inequities |  |  |
| 24 | Agarwal and Koo, 2016^1^, SG [17] | Road pricing (congestion toll rate adjustment) | Overall population: Not available Subgroup measure: Income | PA behaviour - Mode shift to public transport | Increase | Reduced inequities | Review: Bus ridership increased more in lower income areas compared to higher income areas after increases in toll prices.  Primary study: no adaptation | |
| 25 | Chang et al., 2017^1^, MX [18] | New transit infrastructure/access (Bus rapid transit). Setting: Mexico City; transit line. Participants: adults | Overall population: General population Subgroup measure: Education | PA behaviour - Walking for transport females | Increase | Reduced inequities | Review overall effects: Increase in walking for transport (29 minutes per week on average).  Health equity effects: Female participants with low education experienced greater increases in walking for transport compared with female participants with high education.  Primary study: adaptation required  For females recreation and transport walking increases more in low education vs high education group  For males transport walking increases more in low education vs high education, but recreational walking decreases in low education and increases in high education group | |
|  | Chang et al., 2017^1^, MX [18] |  | Overall population: General population Subgroup measure: Education | PA behaviour - Walking for recreation females | Not available in review | Reduced inequities |  |  |
| 25 | Chang et al., 2017^1^, MX [18] |  | Overall population: General population Subgroup measure: Education | PA behaviour - Walking for recreation males | Not available in review | Increased inequities |  |  |
| 25 | Chang et al., 2017^1^, MX [18] |  | Overall population: General population Subgroup measure: Education | PA behaviour - Walking for transport males | Not available in review | Reduced inequities |  |  |
| 26 | Cook et al., 2016, US [19] | A critical link in the American Tobacco Trial: the construction of a bicycle-and-pedestrian bridge over Interstate 40 (I-40) and corresponding paved connections that linked the two unconnected trail segments to form a continuous22-mi shared-use path. The American Tobacco Trail runs on a former railroad corridor south from Durham, North Carolina, and is part of the larger East Coast Greenway network.  Participants: trail users (children and adults) | Overall population: Typical trail user: high education and income Subgroup measure: Income | PA behaviour - Duration of active travel portion of trip | Increase | Increased inequities | Overall effects: An increase in PA on the trail as demonstrated by an increase from 138 to 162 minutes of active travel per week associated with use of the trail. The average duration of the active portion of a trip for those using the American Tobacco Trail after the bridge construction (63 minutes) increased by nearly 7% from that reported in 2013, as shown in Table 3.  No control group.  Health equity effects: Although the proportion of survey respondents in household income brackets of less than $50,000 per year remained relatively the same before and after the bridge construction, duration of the active portion of trips increased after the bridge by 5 minutes for those reporting household incomes of less than $15,000 and by 7 minutes for those with incomes of $30,000 to $44,999. Those with household incomes of $15,000 to $29,999 reported only a slight increase in trip duration. Generally, as household income increases, the average duration of the active portion of one’s trip also increases, a trend that did not change from the before-bridge to the after-bridge surveys. *- no test for sig.* | |
| 27 | Coronini-Cronberg et al., 2012^1^, UK [2] | UK free national bus pass for older adults.  Participants: adults | Overall population: England Subgroup measure: home ownership | PA behaviour - Bus use | Not available in review | No difference | Review results:  Mixed (50% Positive + 50% Neutral) Positive equity impact: The lowest SEP group responded more favourably to the policy relative to the highest SEP group Neutral impact: The lowest and highest SEP groups responded similarly to the policy.  Primary study: no adaptation  In the fully adjusted models, having a bus pass was associated with a greater rate of active travel among those who rented their homes (incidence rate ratio [IRR] = 1.14; 95% CI = 1.04, 1.24; P < .001), but not among those who owned their home (IRR = 1.08; 95% CI = 1.00, 1.17; P < .068). The odds of doing any active travel were similar among pass holders who owned their homes (adjusted odds ratio [AOR] = 4.72; 95% CI = 3.99, 5.59; P < .001) and those who rented their homes (AOR = 4.06; 95% CI = 3.35, 4.86; P < .001).  In the fully adjusted model, the odds of doing any bus travel were similar among pass holders who owned their homes (AOR = 7.11; 95% CI = 5.65, 8.94; P < .001) and those who rented their homes (AOR = 7.03; 95% CI = 5.53, 8.94; P < .001). No sig. results in negative binomial model for rent, bus and own, bus.  We observed a sig. positive association (AOR = 1.15; 95% CI = 1.07, 1.12; P < .001) between having a bus pass and walking 3 or more times a week (Table 4). This association was similar across pass holders who both did and did not own their own homes. | |
| 27 | Coronini-Cronberg et al., 2012^1^, UK [2] |  | Overall population: England Subgroup measure: home ownership | PA behaviour - Walking frequency | Not available in review | No difference |  |  |
| 27 | Coronini-Cronberg et al., 2012^1^, UK [2] |  | Overall population: England Subgroup measure: home ownership | PA behaviour - Active travel | Not available in review | Reduced inequities |  |  |
| 28 | Goodman and Cheshire, 2014a, UK [20] | Extension of BSS to some of London’s poorest areas. No information on participant age.  *Context BSS* *policy*: LBSS was launched by the public body Transport for London on 30th July 2010. The scheme’s bicycles can be taken from any docking station and returned to any other docking station, with the scheme operating 24 h a day, 365 days a year. To hire a bicycle, users can either register online for an access key using a UK credit/debit card (‘registered use’, minimum age 18), or else pay by at docking stations by a UK or international credit/debit card (‘casual use’). [...]. Access originally cost £1 for 1 day, £5 for 7 days, and £45 for 1 year: **from 1st January 2013, these prices doubled.** […] On 8th March 2012 the scheme extended east to cover a larger area. The expanded scheme now encompasses the prosperity of the Canary Wharf business district in Docklands. It also encompasses **much more of London’s relatively deprived East End**, including the poor-quality, high-density housing of London’s poorest borough, Tower Hamlets. | Population: Highly-deprived areas  *No comparison with affluent areas available* | PA behaviour - BSS use - Casual user trips | NA | Reduced inequities | As such, it seems highly likely that the proportion of casual users from more deprived areas increased following the eastern extension, and it is plausible that this occurred to an even more marked degree than was the case for registered users.  *- no test for sig.*  The proportion of trips by registered users from ‘highly-deprived areas’ (in the top tenth nationally for income deprivation) rose from 6% to 12%.  *- no test for sig.* | |
| 28 | Goodman and Cheshire, 2014a, UK [20] |  | Population: Highly-deprived areas *No comparison with affluent areas available* | PA behaviour - BSS use - Registered user trips | NA | Reduced inequities |  |  |
| 28 | Goodman and Cheshire, 2014a, UK [20] | Doubling of BSS prices after extension of BSS to some of London’s poorest areas. No information on participant age.  . | Population: Highly-deprived areas  *No comparison with affluent areas available* | PA behaviour - BSS use - Casual user trips | NA | Increased inequities | This therefore provides indirect evidence that the overall declines in casual use observed after the price increase in January 2013 may have disproportionately occurred among users living in poorer areas. *- no test for sig.*  There was little suggestion of any change in the proportion of registered users living in deprived areas in the 7 months following the price rise *- no test for sig.*  No control group. | |
| 28 | Goodman and Cheshire, 2014a, UK [20] |  | Population: Highly-deprived areas  *No comparison with affluent areas available* | PA behaviour - BSS use - Registered user trips | NA | No difference |  |  |
| 29 | Goodman et al., 2014b^1^, UK [21] | Cardiff: a traffic-free bridge was built over Cardiff Bay; Kenliworth: a traffic-free bridge was built over a busy trunk road; Southampton: informal riverside footpath was turned in to a boardwalk.  Participants: adults (18-89) | Overall population: Not available Subgroup measure: Employment | PA behaviour - Walking and cycling | Increase | No difference | Review: Overall effects: Review Table 1.  At year 2, individuals living closer to the intervention reported sig. increases in walking and cycling relative to those living farther away (an effect of 15.3 minutes per week per kilometre closer to the intervention; 95% CI 6.5, 24.2 minutes per week).  Health equity effects: No sig. interaction between the intervention and education, income or employment when using walking and cycling as the outcome.  Primary study: no adaptation | |
| 29 | Goodman et al., 2014b^1^, UK [21] |  | Overall population: Not available Subgroup measure: Income | PA behaviour - Walking and cycling | Increase | No difference |  |  |
| 29 | Goodman et al., 2014b^1^, UK [21] |  | Overall population: Not available  Subgroup measure: Education | PA behaviour - Walking and cycling | Increase | No difference |  |  |
| 30 | Iroz-Elardo et al., 2020, US [22] | San Joaquin Council of Governments 2018 Regional Transportation plan: New housing units, higher levels of expanded transit (bus rapid transit and commuter rail) and active transportation facilities. These changes to the built environment were spread across the region. Modelling study. | Overall population: entire county  Subgroup measure: Poverty areas | PA behaviour - Transport walking | Increase | Reduced inequities | Overall effects: Predicted walking for transport and leisure and body mass index results for the entire county overall indicate that all three scenarios are expected to modestly improve health (Table 2).  Health equity effects: Table 5: For poverty: areas of concern had a lower predicted increase in leisure walking and higher predicted increase in transport walking compared to balance of region. *- no test for sig.*  An equity area performing better than the balance of the region suggests equity gains from the planned investments of the regional transportation plan.  Comparison group: balance of the region | |
| 30 | Iroz-Elardo et al., 2020, US [22] |  | Overall population: entire county  Subgroup measure: Poverty areas | PA behaviour - Leisure walking | Increase | Increased inequities |  |  |
| 31 | Karlstrom and Franklin, 2009^1^, SE [17] | Road pricing (Stockholm trial) | Overall population: Not available  Subgroup measure: Income | PA behaviour - Mode shift to public transport | Not available in review | No difference | Review: Income not a sig. determinant of switching modes.  Primary study: no adaptation | |
| 32 | Martin et al., 2021, UK [23] | Area-level cycling infrastructure investment  Participants: employed London residents 16-74 years | Overall population: Representative sample London Subgroup measure: SES | PA behaviour - Cycling | Increase | Increased inequities | Overall effects: We conducted a cost-effectiveness analysis which showed that expenditure on cycling infrastructure was associated with increased cycling at a marginal rate of £4915 per additional commuter cyclist, with some variation between groups: ethnic minorities were more responsive, and females, older people and those with lower SES appeared less responsive (Health equity effects). | |
| 33 | Norwood et al., 2014^1^, UK [18] | Active transport intervention (including walking infrastructure). Setting: multiple towns in Scotland.  Participants: adults. | Overall population: General population Subgroup measure: Education | PA behaviour - Meeting PA recommendations | Overall decrease, less in intervention group | Reduced inequities | Review: Overall effects: PA decreased over study period (but less so in intervention group compared to control)  Health equity effects: Statistically sig. positive interaction effect of education (lower education level) on meeting PA recommendations.  Primary study: no adaptation (Fig. 1) | |
| 34 | Panter et al., 2017^1^, UK [18] | Walking and cycling infrastructure. Setting: multiple towns in the UK. Participants: adults | Overall population: General population Subgroup measure: Income | PA behaviour - Walking for transport | Increase | Reduced inequities | Review: Overall effects: Increase in walking for transport  Health equity effects: Indicators of low SES (education level, income, and vehicle ownership) were associated with increases in walking for transport.  Primary study: adaptation required  Table 4  Walking for transport, only no interaction by employment included  Walking for recreation not included by review authors (not socioeconomically patterned) | |
| 34 | Panter et al., 2017^1^, UK [18] |  | Overall population: General population Subgroup measure: Education | PA behaviour - Walking for transport | Increase | Reduced inequities |  |  |
| 34 | Panter et al., 2017^1^, UK [18] |  | Overall population: General population Subgroup measure: Employment | PA behaviour - Walking for transport | Increase | No difference |  |  |
| 34 | Panter et al., 2017^1^, UK [18] |  | Overall population: General population Subgroup measure: Income | PA behaviour - Walking for recreation | Not available in review | No difference |  |  |
| 34 | Panter et al., 2017^1^, UK [18] |  | Overall population: General population Subgroup measure: Education | PA behaviour - Walking for recreation | Not available in review | No difference |  |  |
| 34 | Panter et al., 2017^1^, UK [18] |  | Overall population: General population Subgroup measure: Employment | PA behaviour - Walking for recreation | Not available in review | No difference |  |  |
| **Urban Design** | | | | | | | | |
| 35 | Bohn-Goldhaum et al., 2013^1^, AUS [14] | 1 park underwent renovations: new children's play equipment, upgrading paths, adding new greenery, lighting and facilities (e.g., park furniture), green space was created by opening the adjacent sports field to public use.  Participants: 2-12 y olds and their parents or care givers. | Population: Low socioeconomic neighbourhood | PA behaviour - SOPARC park use | NA | No difference | Review: No sig. Difference between parks for usage or the number of children engaging in MVPA at follow up.  Primary study: no adaptation | |
| 35 | Bohn-Goldhaum et al., 2013^1^, AUS [14] |  | Population: Low socioeconomic neighbourhood | PA behaviour - SOPARC MVPA | NA | No difference |  |  |
| 36 | Brownson et al., 2000, US [24] | Environmental and policy interventions are being conducted in two community-based intervention projects (i.e., the Bootheel and Ozark Heart Health projects) in 12 rural, southeastern Missouri counties, comprising a population of approximately 280,000. These projects are collaborations between the Missouri Department of Health and the Prevention Research Center at Saint Louis University. Compared with the rest of Missouri and the United States, this region has significantly more poverty, is medically underserved, and has lower educational levels. Each project is targeting change in behavioral risk factors for heart disease. […] A major recent focus of the coalitions has been on the construction of new walking trails and enhancement of existing trails. Participants: adults | Overall population: Region with more poverty, and lower educational levels Subgroup measure: Income | PA behaviour - Walking | Increase | Reduced inequities | Overall effects (disadvantaged population): Among persons who had used the trails, 55.2% reported they had increased their amount of walking since they began using the trail.  No control group.  Health equity effects: Nearly 62% of persons with a high school education or less reported increasing walking—this was statistically different from persons with a college education (45.5%). Similarly, lower-income groups were more likely to have increased walking due to trail use than were higher income persons. | |
| 36 | Brownson et al., 2000, US [24] |  | Overall population: Region with more poverty, and lower educational levels Subgroup measure: Education | PA behaviour - Walking | Increase | Reduced inequities |  |  |
| 37 | Cohen et al., 2009^1^, US [25] | Construct or refurbish parks including gymnasiums, fields, paths, picnic and playground areas; 3–14 months’ policy exposure.  Setting: neighbourhoods in a Southern California city  Participants: adults | Population: 31% of neighbourhood households in poverty | PA behaviour - Exercise frequency | NA | No difference | Review: No intervention effect on odds of exercising ≥3 times weekly  Primary study: no adaptation | |
| 38 | Cohen et al., 2012^1^, US [14] | 12 parks involving installation of Family Fitness zones (outdoor gyms), 8 pieces of equipment at each park (average cost $45,000 for each park) Mean park size 14.4 acres (range, 1–29 acres); served an average of 40,964 individuals within 1-mile radius | Population: Mean 29% of households in poverty | PA behaviour - SOPARC park use | NA | No difference | Review: Non-sig increase in park usage (11% in intervention parks compared to control parks)  Primary study: adaptation required  Table 4 PA outcomes:   - No sig. change in METS - No sig. change in average # exercise session/week | |
| 38 | Cohen et al., 2012^1^, US [14] |  | Population: Mean 29% of households in poverty | PA behaviour - METS | NA | No difference |  |  |
| 38 | Cohen et al., 2012^1^, US [14] |  | Population: Mean 29% of households in poverty | PA behaviour – average # exercise sessions/week | NA | No difference |  |  |
| 39 | Cohen et al., 2014^1^, US [14] | Creation of 3 pocket parks (0.15–0.32 acres) from vacant lots and undesirable urban parcels; playground equipment and benches installed, walking path developed around the perimeter, all fenced and enclosed by lockable gates (average cost $1 m funded by local non-profit groups) | Population: 30–41% household poverty | PA behaviour - SOPARC park use | NA | No difference | Review: Pocket parks were used as frequently or more often than playground areas in neighbourhood parks. However, they were vacant during the majority of observations.  There was no evidence to support the provision of pocket parks for increased usage and PA  Primary study: no adaptation | |
| 39 | Cohen et al., 2014^1^, US [14] |  | Population: 30–41% household poverty | PA behaviour - SOPARC PA | NA | No difference |  |  |
| 40 | Droomers et al., 2015^1^, NL [14] | Dutch District Approach (5 million euros): new public parks replacing vacant land (n = 9), refurbishing existing parks (n = 9), n = 6 improving paths, drainage, landscaping, planting flower bulbs in front yards; constructing wall gardens; greening streets, developing a greenway.  Setting: 24 neighbourhoods | Population: Severely deprived neighbourhoods | PA behaviour - Not specified | NA | No difference | Review: Intervention areas did not show an increase in PA and general health compared to the different groups of control areas for adults.  No impact on PA and general health following a suite of park-based and greening interventions (costing EUR 5 million) in 24 severely deprived neighbourhoods in the Netherlands compared to control areas.  Primary study: no adaptation | |
| 41 | Dulin-Keita et al., 2015, US [26] | HOPE VI (Housing Opportunities for People Everywhere) was implemented in 1992 by an act of the United States' Congress, with the goals of replacing distressed public housing, improving surrounding neighborhoods, reducing the concentration of low-income families, and building sustainable communities. Federal level policies and programs such as HOPE VI attempt to revitalize distressed communities by: 1) redressing the effects of concentrated poverty on economically disadvantaged communities; 2) altering the physical environment to create mixed income housing developments in low-income communities; and 3) improving the physical activity environment by incorporating principles of new urbanism to create green spaces such as parks and walking trails that are expected to promote physical activity.  Setting: community in Birmingham, Alabama  Participants: adults (>18 years) | Population: Low SES community | PA behaviour - Leisure time walking/jogging | NA | No difference | Table 4: distance from hope VI, not sig.  No control group. | |
| 42 | Fitzhugh et al., 2010^1^ , US [14] | Retrofit of an urban greenway (2.9 miles long; 8-ft wide) to enhance connectivity of pedestrian infrastructure with nearby retail establishments and schools (cost: $2.1 m). Participants: children, adolescents and adults | Population: 32.2% living in poverty | PA behaviour - Total PA | NA | Reduced inequities | Review: Pre and post intervention changes between experimental and control neighbourhoods were sig different for total PA (p=0.001); walking (p=0.001) and cycling (p=0.038). There was no sig change over time for active transport to school.  sig. positive intervention effect on total PA, walking and cycling.  Primary study: no adaptation | |
| 42 | Fitzhugh et al., 2010^1^ , US [14] |  | Population: 32.2% living in poverty | PA behaviour - Cycling | NA | Reduced inequities |  |  |
| 42 | Fitzhugh et al., 2010^1^ , US [14] |  | Population: 32.2% living in poverty | PA behaviour - Walking | NA | Reduced inequities |  |  |
| 42 | Fitzhugh et al., 2010^1^ , US [14] |  | Population: 32.2% living in poverty | PA behaviour - Active transport to school | NA | No difference |  |  |
| 43 | Gubbels et al., 2016^1^, NL [14] | Dutch District Approach (5 million euros): new public parks replacing vacant land (n = 9), refurbishing existing parks (n = 9), n = 6 improving paths, drainage, landscaping, planting flower bulbs in front yards; constructing wall gardens; greening streets, developing a greenway.  Setting: 24 neighbourhoods  Participants: Adolescents (12–15 years) and adults | Overall population: Severely deprived neighbourhoods  Subgroup measure: education | PA behaviour - Leisure time cycling adolescents | Reduced inequities | No difference | Review: Leisure time walking (decrease 89.2 minutes per week) and cycling (decrease 62.7 minutes per week) significantly decreased.  Here, the greening interventions were associated with a small decline in leisure time cycling.  Primary study: adaptation required  Area level: Greenery interventions were significantly associated with more leisure time cycling in adolescents (β¼0.19, po0.05), but not with any other dependent variable in both adolescents and adults (p40.05; see Table 3). Adolescents who lived in neighborhoods in which greenery interventions were implemented, showed less decrease in their cycling in leisure time (decrease 5.6 min/day, on average) than adolescents who lived in neighborhoods without greenery improvements (14.6 min/day; po0.05)  Subgroup level: no sig. interactions for relevant exposures and outcomes (results not tabulated) | |
| 43 | Gubbels et al., 2016^1^, NL [14] |  | Overall population: Severely deprived neighbourhoods | PA behaviour - Leisure time walking adolescents | No effect | No difference |  |  |
| 43 | Gubbels et al., 2016^1^, NL [14] |  | Subgroup measure: education | PA behaviour - Leisure time cycling adults | No effect | No difference |  |  |
| 43 | Gubbels et al., 2016^1^, NL [14] |  | Overall population: Severely deprived neighbourhoods | PA behaviour - Leisure time walking adults | No effect | No difference |  |  |
| 44 | Kodali et al., 2024, US [27] | the NYC Department of Parks & Recreation (NYC Parks) launched the Community Parks Initiative (CPI) in 2014, a $318 million city-led and equity-based redesign and renovation of 67 neighborhood parks In New York City. Common features of designs included adding more seating and shaded areas, planting more trees and vegetation, restoring lawns, renovating ball courts and playground equipment, and improving the aesthetics of the spaces.  Participants: different age groups (child 12 years or younger, teen aged 13 to 20 years, adult aged 21 to 59 years, or senior 60 years or older). | Population: Low-income neighbourhoods | PA behaviour - Total park use | NA | Reduced inequities | Results difference-in-difference analyses: Overall, the community parks initiative was associated with a greater net number of park users at intervention vs control parks over time, and this association was sustained from wave 2 to wave 3 (wave 2 vs wave 1 difference-in-difference relative rate ratio (DID RRR), 1.59 [95% CI, 1.16-2.18] users/scan [P = .004]; wave 3 vs wave 1 DID RRR, 1.69 [95% CI, 1.22-2.35] users/scan [P = .002])   Furthermore, there was a net positive number of users engaged in walking or VPA at intervention vs control parks over time (wave 2 vs wave 1 DID RRR, 1.84 [95% CI, 1.21-2.80] users/scan [P = .004]; wave 3 vs wave 1 DID RRR, 2.01 [95% CI, 1.28-3.15] users/scan [P = .002]) (Table 2)  After the community parks initiative renovation, a net positive change in MET in intervention vs control parks occurred over time (wave 2 vs wave 1 difference-in-difference absolute difference (DID AD), 0.48 [95% CI, 0.11-0.86] U/scan [P = .01]; wave 3 vs wave 1 DID AD, 0.57 [95% CI, 0.22-0.93] U/scan [P = .002]) (Table 2) | |
| 44 | Kodali et al., 2024, US [27] |  | Population: Low-income neighbourhoods | PA behaviour – Walking or VPA | NA | Reduced inequities |  |  |
| 44 | Kodali et al., 2024, US [27] |  | Population: Low-income neighbourhoods | PA behaviour – MET units | NA | Reduced inequities |  |  |
| 45 | Lopes et al., 2023, BR [28] | The social housing buildings financed by the Minha Casa, Minha Vida (My House, My Life, MCMV) social housing program in Natal, Brazil. The program is an integral component of a national housing policy initiated in 2009 and remains in operation to this day. It is estimated that it has delivered approximately 4 million housing units since its inception.  Spatial data | Overall population: Middle to low income families Subgroup measure: Income | PA environment - Cycling accessibility | Decreased accessibility | Increased inequities | The results of the study revealed a sig. decrease in accessibility across all transportation modes when individuals moved to the new housing estates. The decline was particularly pronounced among individuals with lower incomes, potentially raising their regular expenses after relocation and, ultimately, leading to spatial isolation and social exclusion.  A notable contrast in accessibility emerges among housing projects designed for different income levels. Specifically, developments built for lower-income individuals (level-01) exhibit lower access to essential daily services when compared to their middle-income (level-02 and level-03) counterparts across all scales.  No control group. | |
| 45 | Lopes et al., 2023, BR [28] |  | Overall population: Middle to low income families Subgroup measure: Income | PA environment - Public transport accessibility | Decreased accessibility | Increased inequities |  |  |
| 45 | Lopes et al., 2023, BR [28] |  | Overall population: Middle to low income families Subgroup measure: Income | PA environment - Walking accessibility | Decreased accessibility | Increased inequities |  |  |
| 46 | Peschardt and Stigsdotter, 2014^1^, DK [14] | A pocket park (932m^2^) in a dense urban area was redesigned to increase seating areas and walking trails. | Overall population: No clear indication of disadvantage Subgroup measure: Education | PA behaviour - Park use | No effect | Increased inequities | Review: No sig change in number of park users but demographics of park users changed slightly with more men, people aged 15–29 and more educated people using the park.  Primary study: no adaptation | |
| 47 | Veitch et al., 2012^1^, AUS [14] | 1 park (size 25,200 m^2^): involving establishment of a fenced leash-free area for dogs (12,800m^2^); an all-abilities playground; a 365 m walking track; BBQ area; landscaping; fencing to prevent motor vehicle access to the park | Population: Most disadvantaged decile in state of Victoria | PA behaviour - SOPARC walking | NA | Reduced inequities | Review: Sig. increase from pre to post-improvement in number of park users for intervention park (T1=235, T3=985) and number of people walking (T1=155, T3=369) and being vigorously active (T1=38, T3=257)  Study showed a sig. intervention effect for increases in PA, park usage  and perceptions of safety.  Primary study: no adaptation | |
| 447 | Veitch et al., 2012^1^, AUS [14] |  | Population: Most disadvantaged decile in state of Victoria | PA behaviour - SOPARC vigorously active | NA | Reduced inequities |  |  |
| 47 | Veitch et al., 2012^1^, AUS [14] |  | Population: Most disadvantaged decile in state of Victoria | PA behaviour - SOPARC park use | NA | Reduced inequities |  |  |
| 48 | Zenk et al., 2021, US [29] | Through the Chicago Plays! Initiative, between 2013 and 2016, the Chicago Park District invested $44 million to renovate 327 playgrounds in need of repair across Chicago. A noteworthy feature of the initiative was the engagement of community groups in the renovation process. These playgrounds were disproportionately located in low-income neighborhoods […]. Participants: neighbourhood level data | Overall population: low-income, medium-income, and high-income neighbourhoods (but playgrounds were disproportionately located in low-income neighbourhoods) Subgroup measure: Income | PA behaviour - Park-based MVPA | Not available | Increased inequities | In low-income neighbourhoods, renovations were associated with reductions in park use and park-based MVPA over the longer term. In contrast, renovations were associated with short- and longer-term increases in park use and park-based MVPA in medium-income neighbourhoods and with longer-term increases in MVPA in high-income neighbourhoods. Table 2 | |
| 480 | Zenk et al., 2021, US [29] |  | Overall population: low-income, medium-income, and high-income neighbourhoods (but playgrounds were disproportionately located in low-income neighbourhoods) Subgroup measure: Income | PA behaviour - Park use | Not available | Increased inequities |  |  |
| **Schools** | | | | | | | | |
| 49 | Barbosa Filho et al., 2019, BR [30] | A multicomponent intervention on lifestyle factors among adolescents from schools in low Human Development Index (HDI < 0.500) municipalities in the northeast region of Brazil. The four-month intervention included strategies focused on training teachers, new opportunities for physical activity in the school environment, and health education strategies for the school community (including parents). | Population: Adolescents from schools in low Human Development Index (HDI < 0.500) areas | PA behaviour - Meeting PA guidelines | NA | Reduced inequities | In the intervention schools, a sig. increase occurred in the number of adolescents who met PA guidelines (5.3%; 95% CI = 0.8; 9.8) after intervention. No changes were observed in the control schools. At the end of the intervention, adolescents from intervention schools were more likely to practice PA at recommended levels (OR = 1.44; 95% CI = 1.00; 2.08) than adolescents from control schools. | |
| 50 | Carson et al., 2014, US [31] | A yearlong professional development program designed to equip PE teachers with the knowledge, skills and confidence to become a PA champion who facilitates the implementation of at least one new school PA program beyond the Comprehensive School Physical Activity Program component of PE.  A total of 779 public schools located in 39 parishes with a demographic composition exceeding the 2010–2011 statewide averages in both (a) student poverty level [...], and (b) minority (non-white) student population ( >53%) were targeted for participation in the program (low-income, non-white student population).  Participants: teachers and students | Population: Elementary and middle schools serving primarily low-income student populations | PA behaviour - Student PA during school - girls | NA | Reduced inequities | For in-school MVPA, a sig. condition-by-time interaction effect was observed indicating the control boys and girls spend less time in MVPA by 2.2 minutes d− 1 (95% CI 0.5 to 3.8) and 3.4 minutes d− 1 (95% CI 1.2 to 5.6), respectively, compared to boys and girls attending intervention schools. These data represent an overall in-school decline of ~ 3 minutes d− 1 in control boys and girls compared to a decline of ~ 1 minute d− 1 for girls and no change for boys attending intervention schools.  No changes were observed for total daily MVPA for either boys or girls.  Bonferroni's adjustment technique for multiple comparison presented in Table 1 indicates intervention teachers reportedly provided a significantly more PA offerings during school (3.35 vs. 2.37) and that involve staff (1.43 vs. 0.90), whereas control teachers reportedly provided significantly more before/after school PA offerings (2.25 vs. 1.77). | |
| 50 | Carson et al., 2014, US [31] |  | Population: Elementary and middle schools serving primarily low-income student populations | PA behaviour - Student PA during school - boys | NA | Reduced inequities |  |  |
| 50 | Carson et al., 2014, US [31] |  | Population: Elementary and middle schools serving primarily low-income student populations | PA behaviour - Student total daily PA - girls | NA | No difference |  |  |
| 50 | Carson et al., 2014, US [31] |  | Population: Elementary and middle schools serving primarily low-income student populations | PA behaviour - Student total daily PA - boys | NA | No difference |  |  |
| 50 | Carson et al., 2014, US [31] |  | Population: Elementary and middle schools serving primarily low-income student populations | PA environment - Teacher reported school PA offerings | NA | Mixed |  |  |
| 8 | Cheadle et al., 2018, US [8] | The Healthy Eating Active Living Zones design targeted places and people through policy, environmental, and programmatic strategies. Each Healthy Eating Active Living Zone is a small, low-income community of 10,000 to 20,000 residents with high obesity rates and other health disparities. Community coalitions planned and implemented strategies in each community.  The article focused on two regions: Northern California and Southern California (design and evaluations were closely aligned). Participants: students.  **Parent volunteers, teachers, or YMCA staff were trained to help engage students in steady physical activity during daytime school recess periods.** | Population: Low-income communities | PA behaviour – MVPA overall | NA | Reduced inequities | Pre- to post-MVPA increases ranged from 17% to 19%  Increases in MVPA during recess in 3 of 4 communities.  *- no test for sig.*  No control. | |
| 8 | Cheadle et al., 2018, US [8] |  | Population: Low-income communities | PA behaviour – MVPA during recess | NA | Reduced inequities |  |  |
| 51 | Hobin et al., 2014^1^, CA [2] | Manitoba provincial school PE policy  Participants: children | Overall population: 31 schools in Manitoba Subgroup measure: School neighbourhood disadvantage | PA behaviour - MVPA | Not available in review | Reduced inequities | Review: Positive (The lowest SEP group responded more favourably to the policy relative to the highest SEP group)  Primary study: no adaptation | |
| 52 | Kim, 2012^1^, US [2] | US state and school PE requirements | Overall population: 32 US states with state representative samples of schools and children Subgroup measure: Parental income | PA behaviour - Parent-reported days of week with VPA | Not available in review | No difference | Review: Neutral (The lowest and highest SEP groups responded similarly to the policy)  Primary study: no adaptation | |
| 52 | Kim, 2012^1^, US [2] |  | Overall population: 32 US states with state representative samples of schools and children; Subgroup measure: Parental education | PA behaviour - Parent-reported days of week with VPA | Not available in review | No difference |  |  |
| 53 | Mendoza et al., 2009, US [32] | A walking school bus (WSB) is a group of children who walk to and from school chaperoned by responsible adults, usually parents. The Seattle Public Schools and Feet First, a pedestrian advocacy organization, obtained funding for a single WSB program from the Washington State Department of Transportation. Three public elementary schools were identified and recruited […], based on their diverse and socioeconomically disadvantaged populations.  Participants: students in kindergarten-5^th^ grade (aged 5–11 years) | Population: Low-income, urban community | PA behaviour - Walking to school | NA | Reduced inequities | At 12-month follow up, higher proportions of students (n = 643, p = 0.001)) walked to the intervention (25% +/- 2%) versus the control schools (7% +/- 1%). Comparing baseline to 12-month follow up, the numbers of students who walked to the intervention school increased while the numbers of students who used the other forms of transport did not change (p < 0.0001). In contrast, the numbers of students who walked to the control schools decreased while the numbers of students who used the other forms of transport did not change (p < 0.0001).  Outcome ‘school bus’ not reported/relevant. | |
| 54 | Nathan et al., 2015, AUS [33] | A number of specific state level mandatory policies and recommended programmes targeting the school environment have been published. This study attempts to measure some of these such as; a mandatory policy for Government schools regarding the amount of planned physical activity in the school physical education curriculum (1998); the mandatory teaching of nutrition education through the Personal Development, Health and Physical Education (PDHPE) syllabus (1999) […] To support schools’ adoption of these policies and programmes, the state made available professional learning programmes and resources for teachers regarding nutrition and physical education (2008) and enhanced funding for implementation of such programmes (2011). Participants: primary schools in the state New South Wales | Overall population: Representative randomly selected cohort of primary schools  Subgroup measure: SES | PA environment - School PA policies and practices | No effect on the proportion of schools adopting 3/5 PA policies, decrease in 1/5 policies, and increase in 1/5 policies | No difference | Overall effects: The prevalence of all four of the healthy eating practices and one PA practice significantly increased, while the prevalence of one PA practice significantly decreased. Five PA practices in total.  Health equity effects: No differences were found in the adoption of policies and practices according to socio-economic status.  No control. | |
| 55 | Ridgers et al., 2007^1^, UK [25] | Funding allocated to improve playground environment (new multicolour playground markings and physical structures); 6 weeks’ policy exposure  Setting: 26 schools in one local authority in a city in the Northwest of England  Participants: children | Population: Schools situated in areas of high social and economic deprivation | PA behaviour - VPA during the school day | NA | No difference | Review: No intervention impact on  MVPA or VPA during the  school day.  Primary study: no adaptation | |
| 55 | Ridgers et al., 2007^1^, UK [25] |  | Population: Schools situated in areas of high social and economic deprivation | PA behaviour - MVPA during the school day | NA | No difference |  |  |
| 56 | Sutherland et al., 2016, AUS [34] | A multicomponent school-based intervention based on the Health Promoting Schools Framework was implemented. The intervention consisted of seven physical activity promotion strategies that targeted the curriculum (teaching strategies to increase physical activity in physical education lessons, student physical activity plans, and modification of school sport program); school environment (recess/lunchtime activities, school physical activity policy); parents (parent newsletters); and community (community physical activity provider promotion). Six additional strategies supported school implementation of the physical activity intervention strategies. The trial was undertaken within secondary schools located in disadvantaged communities in New South Wales, Australia, Participants: students aged 12 years at baseline. | Population: Secondary school students in disadvantaged areas | PA behaviour - Percentage wear time in MPA | NA | No difference | At 24-month follow-up, there were sig. effects in favour of the intervention group for daily minutes of MVPA. The adjusted mean difference in change in daily MVPA between groups was 7.0 minutes (95% CI=2.7, 11.4, p<0.002)  There were sig. effects in favour of the intervention group for five of the six secondary PA outcomes: minutes per day of VPA, minutes per day of MPA, percentage wear time in MVPA and VPA, and total daily accelerometer counts. There were no sig. intervention effects for percentage of wear time in MPA. | |
| 56 | Sutherland et al., 2016, AUS [34] |  | Population: Secondary school students in disadvantaged areas | PA behaviour - Minutes per day spent in MPA | NA | Reduced inequities |  |  |
| 56 | Sutherland et al., 2016, AUS [34] |  | Population: Secondary school students in disadvantaged areas | PA behaviour - Percentage wear time in VPA | NA | Reduced inequities |  |  |
| 56 | Sutherland et al., 2016, AUS [34] |  | Population: Secondary school students in disadvantaged areas | PA behaviour - Total daily accelerometer counts | NA | Reduced inequities |  |  |
| 56 | Sutherland et al., 2016, AUS [34] |  | Population: Secondary school students in disadvantaged areas | PA behaviour - Percentage wear time in MVPA | NA | Reduced inequities |  |  |
| 56 | Sutherland et al., 2016, AUS [34] |  | Population: Secondary school students in disadvantaged areas | PA behaviour - Minutes per day spent in VPA | NA | Reduced inequities |  |  |
| 56 | Sutherland et al., 2016, AUS [34] |  | Population: Secondary school students in disadvantaged areas | PA behaviour - Mean student duration (minutes) of MVPA per day | NA | Reduced inequities |  |  |
| 57 | Van der Ploeg et al., 2014, CA [35] | The Alberta Project Promoting Active Living and Healthy Eating in Schools (APPLE Schools) builds on and extends previous school-based health promotion interventions by offering the placement of a full-time staff member dedicated to facilitating healthy living programming and curricula.  Intervention schools had to be located in socioeconomically disadvantaged neighborhoods in Edmonton.  Participants: 5th grade students | Population: Socioeconomically disadvantaged neighbourhoods | PA behaviour - School days, steps/day | NA | Reduced inequities | A sig. interaction was observed between group and time in the adjusted multilevel model, such that children from APPLE Schools experienced increases of 1221 steps per day (95% CI: 306 to 2135) on school days, 2001 steps per day (95% CI: 600 to 3402) on weekend days, and 1399 steps per day (95% CI: 485 to 2312) during a typical week beyond the increases observed on these days among children from comparison schools. The intervention effect was also sig. when assessing the change in hourly steps outside of school hours between APPLE Schools and comparison schools (β** = 137; 95% CI: 31 to 242) Table 2  No control, but comparison group. | |
| 57 | Van der Ploeg et al., 2014, CA [35] |  | Population: Socioeconomically disadvantaged neighbourhoods | PA behaviour - Non–school days, steps/day | NA | Reduced inequities |  |  |
| 57 | Van der Ploeg et al., 2014, CA [35] |  | Population: Socioeconomically disadvantaged neighbourhoods | PA behaviour - Typical week, steps/day | NA | Reduced inequities |  |  |
| 57 | Van der Ploeg et al., 2014, CA [35] |  | Population: Socioeconomically disadvantaged neighbourhoods | PA behaviour - Non–school hours, steps/hour | NA | Reduced inequities |  |  |
| 57 | Van der Ploeg et al., 2014, CA [35] |  | Population: Socioeconomically disadvantaged neighbourhoods | PA behaviour - School hours, steps/hour | NA | No difference |  |  |
| **Sport for all** | | | | | | | | |
| 58 | Andrade et al., 2018, BR [36] | *Academias da Cidade* Program: The program aims to promote physical activity and improve the quality of life of the population by providing access to free activities such as gymnastics, dance, games, sports, fights, and walking, all of which are guided by a Physical Education professional. The activities are offered preferably for adults over 18 years, three times a week and one hour a day in up to two times per day. Admission is by referral from the basic health unit or by spontaneous desire. The centers are strategically installed in areas of social vulnerability and in public places. The municipality of Belo Horizonte has 75 program centers integrated into the Brazilian Unified Health System and composed of spaces with infrastructure, equipment and qualified human resources to guide bodily practices, physical activity and healthy lifestyles. | Population: The centres are strategically installed in areas of social vulnerability | PA behaviour – Leisure time PA | NA | Reduced inequities | The overall prevalence of the PA in leisure time was 26.5% in the exposed group and 22.7% in the unexposed group. The exposed group was more likely to be active in leisure time (OR = 1.05; CI 95%: 1.01-1.10). When considering the interaction between exposed group and distance, individuals in the exposed group who lived less than 500 meters from the program centre were more likely to be active in leisure time (OR = 1.18, CI 95%: 1.03-1.35) compared to their counterparts. | |
| 59 | Candio et al., 2020, UK [37] | Leeds Let’s Get Active (LLGA) was a city-wide programme developed by the Local Authority and funded in collaboration with Sport England and Public Health England, which was aimed to reduce physical inactivity levels in the local adult population by the provision of universal access to free off-peak City Council leisure centre-based exercise sessions to all city residents. In order to encourage residents from low socio-economic backgrounds to take up the offer, LLGA sessions were provided in 17 centres located in the most deprived areas of the city (i.e. proportionate universal offer). Exercise sessions included the use of free weight areas, swimming pool access and fitness classes. | Overall population: 80.5% living in non-deprived areas of the city  Subgroup measure: deprivation | PA behaviour – Active days in past week | Increase | Reduced inequities | Overall effects: 50.5% increased their baseline PA category, 36.9% did not change it, whereas 12.4% reported a lower PA level.  Health equity effects: Participants from deprived areas started at an overall lower PA level at baseline than the non-deprived group. Post-registration distributions of PA categories were found to be comparable between the two subgroups, indicating an only marginal difference in terms of intervention effect. Table 2.   - *no test for sig.* | |
| 60 | Higgerson et al., 2018a, UK [38] | Blackpool Borough Council provides free use of its two swimming pools for children under 16 years old during the school holidays. The council has funded this free offer since 2010 when the national free swimming scheme for children ended. We investigated the impact of this free offer to children in Blackpool in 2014. Blackpool is a deprived local authority in the North West of England. | Overall population: Areas of deprivation in the UK  Subgroup measure: Area deprivation | PA behaviour – Children swimming per 100 population | Increase | No difference | Overall effects (disadvantaged): Free swimming during the summer holidays was associated with an additional 6% of children swimming and an additional 33 swims per 100 children per year.  Health equity effects: Figure 2 shows the estimates of the effect of the free-swimming offer from the comparative regression discontinuity model. Overall the free swimming offer was associated with an additional 6 children swimming per 100 children per year (95% CI: 4–9). There was no significant difference in this outcome across deprivation quintiles, although the effect was greatest in quintile 3. Overall the free-swimming offer was associated with an additional 33 swims per 100 population per year (95% CI: 21–44). This rate was higher in quintiles 3 and 4, but lower in the most and least deprived areas of Blackpool. In terms of the number of additional swims the effect in the least deprived areas was not statistically sig. at the 5% level.  *comparison quantile 1 and quantile 5. | |
| 60 | Higgerson et al., 2018a, UK [38] |  | Overall population: Areas of deprivation in the UK  Subgroup measure: Area deprivation | PA behaviour – Swims per 100 population | Increase | Reduced inequities |  |  |
| 61 | Hoekman et al., 2017, NL [39] | Municipal sport policy programs – Neighbourhood Sport Coaches who initiate sport activities in lower socio-economic status neighbourhoods | Overall population: Nationally representative sample Subgroup measure: Education | PA behaviour – Sport club participation in adults | No effect | Increased inequities | Overall effects: Municipal sport expenditures per inhabitant are positively related to sport participation and sport club participation for youth. The Youth Sport Fund and the Neighbourhood Sport Coaches are not related to more sport (club) participation for youth.  For adults (see Table 2b), a sig. negative relation was found between municipal sport expenditures and Neighbourhood Sport Coaches and sport participation. No sig. effects were found between aspects of sport policy (sport expenditures and policy programs) and sport club participation of adults.  Health equity effects: Evidence for the assumed cross-level interaction (macro-micro level) of the municipal sport expenditures and policy programs with individual socio-economic position were only detected with regard to sport club participation.  Figure 1 shows, in line with the compensation premise, a negative cross-level interaction of individual household income and municipal sport expenditures for sport club participation of youth. Higher municipal sport expenditures thus correspond with smaller differences in sport club participation between youth of higher and lower household incomes.  For adults, no cross-level interactions were found for municipal sport expenditures on either sport club participation or sport participation.  Contrary to our expectations a negative cross-level interaction was noted for sport club participation of adults for lower income groups and lower educational levels, meaning that the participation gap between income groups increases with an increasing number of Neighbourhood Sport Coaches per 10.000 inhabitants. | |
| 61 | Hoekman et al., 2017, NL [39] | Municipal sport policy programs – Neighbourhood Sport Coaches who initiate sport activities in lower socio-economic status neighbourhoods | Overall population: Nationally representative sample Subgroup measure: Income | PA behaviour – Sport club participation in adults | No effect | Increased inequities |  |  |
| 61 | Hoekman et al., 2017, NL [39] | Municipal sport policy programs – Neighbourhood Sport Coaches (i.e. initiate sport activities in lower socio-economic status neighbourhoods) and Youth Sport Fund (i.e. covers the costs of sport participation for people from low income households) | Overall population: Nationally representative sample Subgroup measure: socio-economic position | PA behaviour – Sport club participation in children | No effect | No difference |  |  |
| 61 | Hoekman et al., 2017, NL [39] | Higher municipal sport expenditure | Overall population: Nationally representative sample Subgroup measure: socio-economic position | PA behaviour – Sport club participation in adults | No effect | No difference |  |  |
| 61 | Hoekman et al., 2017, NL [39] | Higher municipal sport expenditure | Overall population: Nationally representative sample Subgroup measure: Income | PA behaviour – Sport club participation in children | Increase | Reduced inequities |  |  |
| 61 | Hoekman et al., 2017, NL [39] | Municipal sport policy programs – Neighbourhood Sport Coaches who initiate sport activities in lower socio-economic status neighbourhoods | Overall population: Nationally representative sample Subgroup measure: socio-economic position | PA behaviour - Sport participation in adults | Decrease | No difference |  |  |
| 61 | Hoekman et al., 2017, NL [39] | Municipal sport policy programs – Neighbourhood Sport Coaches (i.e. initiate sport activities in lower socio-economic status neighbourhoods) and Youth Sport Fund (i.e. covers the costs of sport participation for people from low income households) | Overall population: Nationally representative sample Subgroup measure: socio-economic position | PA behaviour - Sport participation in children | No effect | No difference |  |  |
| 61 | Hoekman et al., 2017, NL [39] | Higher municipal sport expenditure | Overall population: Nationally representative sample Subgroup measure: socio-economic position | PA behaviour - Sport participation in adults | Decrease | No difference |  |  |
| 61 | Hoekman et al., 2017, NL [39] | Higher municipal sport expenditure  Participants: children (6-17 y) and adults (25-79 y) living in 399 Dutch municipalities. | Overall population: Nationally representative sample Subgroup measure: socio-economic position | PA behaviour - Sport participation in children | Increase | No difference |  |  |
| 62 | Rabiee et al., 2015, UK [40] | The Gym For Free scheme, a joint initiative between a Primary Care Trust (PCT) and the local authority in Birmingham. The scheme started without any feasibility or needs assessment. There was a perception among local professionals that cost could be a barrier to the uptake of leisure facilities. To test this perception, the Heart of Birmingham PCT transferred £500,000 towards free access to leisure facilities for adults in one economically deprived constituency in the city for 6 months. Access remained free if adults used this service a minimum of four times per month. | Population: Deprived areas | PA behaviour - Use of leisure facilities | NA | Reduced inequities | Findings showed that the pilot scheme increased the uptake of exercise particularly for women in an economically deprived inner city area. The use of leisure facilities also increased markedly (p<.05).  No control. | |
| 63 | Reilly et al., 2021, AUS [41] | In New South Wales (NSW), the state government introduced the Active Kids (AK) scheme in 2018. The AK scheme is a four year investment of greater than $200 million, to help families across the entire state meet the cost of getting children into organized sport and recreation activities. The AK scheme aims to reduce the financial barrier to child organized sport participation and help increase the physical activity behaviours of children 4.5 to 18 years of age. In 2018, parents/carers of school-enrolled children in NSW were able to register for one $100 AK voucher valid for redemption throughout the calendar year, which was only to be claimed through an online government platform. Vouchers can only be redeemed through an accredited provider authorised by NSW government. Physical activity providers eligible to register as an ‘accredited AK provider’ are those that provide activities including participation in moderate intensity organised sport, lasting a minimum of eight weeks. | Overall population: Low socio-economic index (68%) Subgroup measure: SES | PA behaviour - PA outside of school hours | No effect | No difference | Overall effects (disadvantaged): Those that redeemed the voucher had three times the odds of organized team sports participation than those that did not. No changes for individual sports.  Additionally, there were no sig. differences in changes in child PA among those that redeemed and did not redeem a voucher.  Health equity effects: Table 3, 4: subgroup analyses SES (not sig.) | |
| 63 | Reilly et al., 2021, AUS [41] |  | Overall population: Low socio-economic index (68%) Subgroup measure: SES | PA behaviour - PA on weekends | No effect | No difference |  |  |
| 63 | Reilly et al., 2021, AUS [41] |  | Overall population: Low socio-economic index (68%) Subgroup measure: SES | PA behaviour - Participation in organised team sports | Increase | No difference |  |  |
| 63 | Reilly et al., 2021, AUS [41] |  | Overall population: Low socio-economic index (68%) Subgroup measure: SES | PA behaviour - Participation in organised individual sports | No effect | No difference |  |  |
| 64 | Taylor et al., 2011, UK [42] | Central government subsidies for local authority sport and leisure centres.  Participants: young people and adults | Population: Unemployed | PA behaviour - Sports and leisure centre usage | NA | Increased inequities | Performance of the two lowest socioeconomic groups appears to have improved strongly, after a fall in 2001. However, this is an illusion because the statistical definition and measurement of these groups changed radically for the 2006 indicator, as explained in the Methods section. Therefore, the increase shown reflects the change in the way socioeconomic groups are identified, rather than real increases in the two lowest groups' participation.  For the disabled and the unemployed, the evidence is of slight decline in usage over the 10 years covered. *- no test for sig.*  No control. | |
| 64 | Taylor et al., 2011, UK [42] |  | Population: Low socioeconomic groups | PA behaviour - Sports and leisure centre usage | NA | Unclear impact |  |  |
| 65 | Virmasalo et al., 2023, FI [43] | Closure and restrictions, concerning indoor sport facilities and organized physical activity (during COVID-19).  The present study focuses on the residents of two case areas: the suburb of Huhtasuo is located in the city of Jyväskylä in central Finland, and the Kontula suburb is part of the capital city, Helsinki, in southern Finland.  Participants: adults (18-79 y) | Overall population: Lower SES  Subgroup measure: Education | PA behaviour - PA levels | Decrease | No difference | Overall effects (disadvantaged): figure 2 and figure 3. Overall effect is not relevant; not a specific policy for a disadvantaged population.  Health equity effects: A risk of reduced use of indoor sport facilities was associated with the female gender, higher education, and higher reported PA level. Among those who had reduced their use of indoor sport facilities, a decrease in overall PA was associated with the female gender and a non-native language. In general, PA declined, but indoor PA was often replaced through other environments. The constraints on indoor sport facilities seem to not have intensified the socioeconomic polarisation of PA.   The low importance of SES is noteworthy: low labour market status, income level or education do not constitute a sig. risk for a decrease in total PA.  No control. | |
| 65 | Virmasalo et al., 2023, FI [43] |  | Overall population: Lower SES Subgroup measure: Employment | PA behaviour - PA levels | Decrease | No difference |  |  |
| 65 | Virmasalo et al., 2023, FI [43] |  | Overall population: Lower SES  Subgroup measure: Income | PA behaviour - PA levels | Decrease | No difference |  |  |
| 65 | Virmasalo et al., 2023, FI [43] |  | Overall population: Lower SES  Subgroup measure: Education | PA behaviour - Use of indoor sport facilities for PA | Decrease | Reduced inequities |  |  |
| 65 | Virmasalo et al., 2023, FI [43] |  | Overall population: Lower SES Subgroup measure: Employment | PA behaviour - Use of indoor sport facilities for PA | Decrease | No difference |  |  |
| 65 | Virmasalo et al., 2023, FI [43] |  | Overall population: Lower SES Subgroup measure: Income | PA behaviour - Use of indoor sport facilities for PA | Decrease | No difference |  |  |
| 66 | Williams, 2017, UK [44] | The city's new flagship swimming pool and leisure centre in North Kingsland, a low SES-neighbourhood in England. This was a local government project subsequently incorporated into the regeneration plan. Kingsland Leisure Centre opened in 2004. It cost approximately £10 million to build with approximately £1 million sourced from NDC funds. The leisure centre drifted away from initial commitments to equitable service access.  Participants: adults? | Population: Deprived neighbourhood | PA behaviour - Visits to leisure centre | NA | Increased inequities | Through a process of inequity drift access to the Leisure Centre became less equitable over time. Initially, the area based interventions transformed the physical environment and provided targeted support that addressed multiple structural barriers that inhibited residents’ PA. However, the downgrading and removal of services that accompanied these improvements to the local environment made access to the Leisure Centre less equitable and thus, like other untargeted interventions, it became increasingly liable to reproducing the inequality paradox. The relatively high initial participation rates of North Kingsland residents, combined with the gradual decline in services to support their PA, suggests that potentially reproducing the inequality paradox was not an inevitable outcome due to the compositional characteristics of the target group *- no test for sig.* | |
| **Mass media** | | | | | | | | |
| 67 | Bauman et al., 2001^1^ ,AUS [45] | Mass media campaign targeting a whole population, including paid television advertising  Participants: adults (25-60 years) | Overall population: Representative population sample Subgroup measure: Education | PA behaviour - Not specified | Not available in review | No difference | Review: No difference in increased PA pre to post between three education levels  Primary study: no adaptation | |
| 68 | Booth et al., 1992^1^, AUS [45] | Mass media campaign targeting a whole population, including paid television advertising  Participants: 14-60+ years/ general population | Overall population: Representative population sample Subgroup measure: Education | PA behaviour - Walking | Not available in review | Reduced inequities | Review: Increase in walking only sig. for least educated 66.3– 72.6% (z=2.74 P=0.006)  Primary study: no adaptation | |
| 69 | Croker et al., 2012^1^, UK [45] | Mass media campaign targeting a whole population, including paid television advertising  Participants: parents of 5-11 year old children. | Overall population: Representative population sample Subgroup measure: Education | PA behaviour - Days/week active | No effect | No difference | Review: No sig difference in treatment effect (intervention versus control) and no interaction effect (education groups) for childrens’ days/week active  Primary study: no adaptation | |
| 70 | Hillsdon et al., 2001^1^, UK [45] | Mass media campaign targeting a whole population, including paid television advertising  Participants: 16-74 years/ general population | Overall population: Representative population sample Subgroup measure: Social grade (based on occupation) | PA behaviour - Meeting moderate/vigorous PA guidelines | Not available in review | No difference | Review: No difference in % meeting moderate/ vigorous PA guidelines by social grade  Primary study: no adaptation | |
| 71 | Leavy et al., 2013^1^, AUS [45] | Mass media campaign targeting a whole population, including paid television advertising  Participants: General adult population (specifically those 20–54 years) | Overall population: Representative population sample Subgroup measure: Education | PA behaviour - Not specified | Not available in review | Increased inequities | Review: University education (Adjusted Odds Ratio (AOR) 1.85, 95% CI 1.50–2.28) more likely to achieve PA post-campaign than those with less than TEE  Primary study: no adaptation | |
| 72 | Leavy et al., 2014^1^, AUS [45] | Mass media campaign targeting a whole population, including paid television advertising  Participants: General adult population (specifically those 20–54 years) | Overall population: Representative population sample Subgroup measure: Education | PA behaviour - Total PA | Not available in review | No difference | Review: Highest educated had sig. decrease in weekly minutes walking T1–T4 (P<0.001); no sig difference in weekly minutes walking between high and low SES; no sig difference in total PA by education or SES  Primary study: no adaptation | |
| 72 | Leavy et al., 2014^1^, AUS [45] |  | Overall population: Representative population sample Subgroup measure: SES | PA behaviour - Total PA | Not available in review | No difference |  |  |
| 72 | Leavy et al., 2014^1^, AUS [45] |  | Overall population: Representative population sample Subgroup measure: SES | PA behaviour - Weekly minutes walking | Not available in review | No difference |  |  |
| 72 | Leavy et al., 2014^1^, AUS [45] |  | Overall population: Representative population sample Subgroup measure: Education | PA behaviour - Weekly minutes walking | Not available in review | Reduced inequities |  |  |
| 73 | Owen et al., 1995^1^, AUS [45] | Mass media campaign targeting a whole population, including paid television advertising  Participants: 14+ years/ general population | Overall population: Representative population sample Subgroup measure: Education | PA behaviour - Walking | Not available in review | No difference | Review: No sig difference between education groups in likelihood of walking, for 1990 campaign; no sig difference between education groups in likelihood of walking, for 1991 campaign  Primary study: no adaptation | |
| 74 | Pena-Y-Lillo and Lee, 2019, US [46] | VERB was the first publicly funded mass media campaign aimed to promote exercise among children between ages 9 and 13 (tweens). It was created by the Centers for Disease Control and Prevention (CDC). The VERB campaign devoted a big portion of its budget ($125 million only in its first year) to paid advertising on cable television networks and specialized magazines popular with the target audience. The campaign also utilized donated media time, Internet websites, radio, and community- and school-based activities. The literature has not noted any additional effort by the VERB campaign to tailor the messages for low-SES tweens.  Participants: child-parent dyads | Overall population: Nationally representative sample Subgroup measure: Education | PA behaviour - Number of organized time PA sessions | Increase | No difference | Figure 2 and 3 are discussed within research team (FH & NRdB). | |
| 74 | Pena-Y-Lillo and Lee, 2019, US [46] |  | Overall population: Nationally representative sample Subgroup measure: Income | PA behaviour - Number of organized time PA sessions | Increase | Increased inequities |  |  |
| **Healthcare** | | | | | | | | |
| 75 | Davison et al., 2011^1^, UK [25] | Integration of a community resource guide that links families with local PA resources into the Special Supplemental Nutrition Program for Women, Infants and Children (WIC) counselling sessions with parents;1-year policy exposure  Setting: 4 WIC clinics in New York  Participants: adults and children. | Population: All participants enrolled in a Special Supplemental Nutrition Program for Women, Infants and Children (WIC) | PA behaviour - Meeting PA recommendations parents | NA | No difference | Review: Intervention and control group parents had equal odds of meeting PA recommendations at follow-up. Parents in intervention group more likely to report their child plays outdoors for ≥60 minutes daily at follow-up  Primary study: no adaptation | |
| 75 | Davison et al., 2011^1^, UK [25] |  | Population: All participants enrolled in a Special Supplemental Nutrition Program for Women, Infants and Children (WIC) | PA behaviour - Outdoor play children ≥60 minutes daily | NA | Reduced inequities |  |  |
| 76 | Tomioka et al., 2012, HI [47] | Stanford’s Chronic Disease Self-Management Program aims to increase self-efficacy through six weekly, 2.5-hr sessions that promote mastery of new knowledge (e.g., about chronic disease) and skills (e.g., in health care communication and goal setting). Leaders model good self-management behaviors, and the group engages in problem-solving activities, support, and feedback sessions each week. Participants: older Asian and Pacific Islander adults with low levels of education and income. | Population: Low education and income | PA behaviour - Aerobic exercise | NA | No difference | Native Hawaiians and Pacific Islanders - the intervention resulted in a sig. increase in minutes/week of both strengthening, stretching and non sig. increase in aerobic exercise.  No control. | |
| 76 | Tomioka et al., 2012, HI [47] |  | Population: Low education and income | PA behaviour - Strengthening/stretching | NA | Reduced inequities |  |  |
| **Childcare** | | | | | | | | |
| 8 | Cheadle et al., 2018, US [8] | The Healthy Eating Active Living Zones design targeted places and people through policy, environmental, and programmatic strategies. Each Healthy Eating Active Living Zone is a small, low-income community of 10,000 to 20,000 residents with high obesity rates and other health disparities. Community coalitions planned and implemented strategies in each community.  The article focused on two regions: Northern California and Southern California (design and evaluations were closely aligned) Participants: early childhood care sites.  **In Community 1, 24 small early childhood care sites worked with a YMCA Health Educator to increase healthy offerings. In Community 2, one center serving 144 children was certified as a healthy site, by offering more fruits and vegetables and making other health-promoting changes.** | Population: Low-income communities | PA environment - % of sites providing more than 60 minutes of PA | NA | Reduced inequities | Percentage of sites providing more than 60 minutes of PA increased from 56% to 71% *- no test for sig.* | |
| 77 | Esquivel et al., 2016, HI [48] | A child care centers based intervention that used training and technical assistance and employee wellness activities in collaboration with Head Start teachers to help with the implementation of Head Start wellness policies for childhood obesity prevention in Hawaii. Head Start is a federally funded preschool program serving low-income children 3–5 years of age across the United States and its jurisdictions.  This research was embedded within the randomized community trial, the Children's Healthy Living Program for Remove Underserved Minority Populations in the Pacific Region (CHL). Communities for the research project were chosen from four randomized CHL communities in Hawaii. | Population: Low-income children | PA environment - Classroom EPAO PA | NA | Reduced inequities | The intervention showed a positive and sig. effect on classroom EPAO PA and EPAO total scores | |
| 78 | Tomayko et al., 2017, US [49] | A statewide partnership developed the Active Early guide to target increasing physical activity opportunities in early care and education (ECE) settings. The aim of Active Early 2.0 was to evaluate the Active Early guide in combination with technical support and microgrant assistance in ECE settings serving a high proportion of children from families of lower socioeconomic status [...]. | Population: Low SES | PA environment - Written activity policy | NA | Reduced inequities | Overall minutes of *total* teacher-led PA increased to 61.5 ± 29.0 minutes (p < 0.05). (indoor sig., outdoor non-sig.) We did not observe improvements in child PA as measured by accelerometery. There was no significant change in Total PA Score or any EPAO subscale over the intervention period. Minutes of outdoor playtime did not significantly change over the intervention period (p = 0.13). The percentage of sites with written activity policies significantly increased.  No control. | |
| 78 | Tomayko et al., 2017, US [49] |  | Population: Low SES | PA environment - Observed free playtime | NA | No difference |  |  |
| 78 | Tomayko et al., 2017, US [49] |  | Population: Low SES | PA environment - EPAO total and subscale scores | NA | No difference |  |  |
| 78 | Tomayko et al., 2017, US [49] |  | Population: Low SES | PA environment - Observed minutes of teacher-led PA | NA | Reduced inequities |  |  |
| 78 | Tomayko et al., 2017, US [49] |  | Population: Low SES | PA behaviour - PA measured by accelerometery | NA | No difference |  |  |
| 79 | Yoong et al., 2016, AUS [50] | To provide contextual information regarding the external environment in which this study was conducted state specific programmes which may have facilitated the adoption of healthy eating and physical activity practices in the childcare sector during this time are briefly described. Such programmes include the Good for Kids, Good for Life programmes in the Hunter New England Region in 2006 and The Munch and Move program introduced in NSW from 2008. Briefly, these programmes sought to support childcare service staff with implementing healthy eating and physical activity promoting practices by providing staff training and support offered by local health promotion units within the region.  Participants: childcare services in the state of New South Wales | Overall population: Randomly selected proportion of childcare services within an entire jurisdiction  Subgroup measure: SES | PA environment - Written PA policies, staff trained in PA, scheduled time for fundamental movement skills and outdoor play | Increase in the prevalence of all PA services, except for outdoor play | No difference | Overall effects: A sig. increase in the prevalence of services adopting all but one (PA) practice, between 2006 and 2013 was identified. Ninety one percent of services adopted four or more practices, a sig. increase from 38% in 2006.  No control.  Health equity effects: No sig. difference by locality or SES was identified when examining adoption of practices in 2013. Table 3 | |
| **Social** | | | | | | | | |
| 80 | Vahid Shahidi et al., 2019, US, CA [51] | In each country, we examined the largest social assistance programmes that provide means-tested cash benefits to individuals who demonstrate both financial need and a willingness to work.  US: federal Temporary Assistance for Needy Families (TANF) programme.  CA: main provincial social assistance programmes (eg, Ontario Works).  Participants: 18-64 years | Overall population: Low-income Subgroup measure: Employment | PA behaviour - PA inactivity | Not available | No difference | Differences in the remaining outcomes (e.g., PA) were not statistically sig.. | |
| 81 | Spence et al., 2010^1^, CA [2] | Canadian children's Fitness Tax Credit  Participants: adults | Overall population: Nationally representative sample of Canadian parents from an online panel  Subgroup measure: Income | PA behaviour – Participation of children in organized PA | Not available | Reduced inequities | Review: PA: Positive  Primary study: no adaptation  Among parents who had claimed the CFTC for 2007, 15.6% agreed the CFTC had increased their child's participation in organized PA. Level of agreement varied by household income, *χ* *^2^*(3, 262) = 14.69, *p* = .002, with those in the lowest-income quartile (37.5%) being much more likely to agree the CFTC increased their child's PA than those in the second (24.4%), third (11.5%), or highest-income quartile (10.4%). | |

*BSS: Bike Share System, CI: Confidence Interval, EPAO: Environment and Policy Assessment and Observation, MET: Metabolic Equivalent of a Task, MVPA: Moderate-to-Vigorous Physical Activity, NA: Not Applicable, OR: Odds Ratio, PA: Physical Activity, PE: Physical Education, SEP: Socioeconomic Position, SES: Socioeconomic Status, sig.: significant, SOPARC: System for Observing Play and Recreation in Communities, T = timepoint, VPA: Vigorous Physical Activity.*

^1^ Result extracted from review: Hansmann et al., 2022 [18], Hosford et al., 2021 [17], Hunter et al., 2019 [14], Nickel and von dem Kneseback, 2020 [6], Olstad et al., 2017 [25], Olstad et al., 2016 [2], Smith et al., 2017 [21], and Thomas et al., 2018 [45].

^2^ Adaptation refers to harmonisation steps required to align data with the predefined extraction framework.

**References**

1. Andersen HB, Christiansen LB, Klinker CD, Ersbøll AK, Troelsen J, Kerr J, et al. Increases in Use and Activity Due to Urban Renewal: Effect of a Natural Experiment. Am J Prev Med. 2017;53(3):e81–e7. <https://doi.org/10.1016/j.amepre.2017.03.010>.

2. Olstad DL, Teychenne M, Minaker LM, Taber DR, Raine KD, Nykiforuk CI, et al. Can policy ameliorate socioeconomic inequities in obesity and obesity-related behaviours? A systematic review of the impact of universal policies on adults and children. Obes Rev. 2016;17(12):1198–217. <https://doi.org/10.1111/obr.12457>.

3. Aytur SA, Rodriguez DA, Evenson KR, Catellier DJ, Rosamond WD. The sociodemographics of land use planning: relationships to physical activity, accessibility, and equity. Health Place. 2008;14(3):367–85. <https://doi.org/10.1016/j.healthplace.2007.08.004>.

4. Bijlani C, Vrinten C, Junghans C, Chang K, Lewis E, Mulla U, et al. Changes in diet and physical activity following a community-wide pilot intervention to tackle childhood obesity in a deprived inner-London ward. BMC Public Health. 2024;24(1):800. <https://doi.org/10.1186/s12889-024-18192-8>.

5. Boelens M, Raat H, Jonkman H, Hosman CMH, Wiering D, Jansen W. Effectiveness of the Promising Neighbourhoods community program in 0-to 12-year-olds: A difference-in-difference analysis. SSM Popul Health. 2022;19:101166. <https://doi.org/10.1016/j.ssmph.2022.101166>.

6. Nickel S, von dem Knesebeck O. Do multiple community-based interventions on health promotion tackle health inequalities? INTERNATIONAL JOURNAL FOR EQUITY IN HEALTH. 2020;19(1):157. <https://doi.org/10.1186/s12939-020-01271-8>.

7. Buscemi J, Odoms-Young A, Stolley MR, Schiffer L, Blumstein L, Clark MH, et al. Comparative Effectiveness Trial of an Obesity Prevention Intervention in EFNEP and SNAP-ED: Primary Outcomes. Nutrients. 2019;11(5). <https://doi.org/10.3390/nu11051012>.

8. Cheadle A, Atiedu A, Rauzon S, Schwartz PM, Keene L, Davoudi M, et al. A Community-Level Initiative to Prevent Obesity: Results From Kaiser Permanente's Healthy Eating Active Living Zones Initiative in California. Am J Prev Med. 2018;54(5 Suppl 2):S150–s9. <https://doi.org/10.1016/j.amepre.2018.01.024>.

9. Derose KP, Williams MV, Flórez KR, Griffin BA, Payán DD, Seelam R, et al. Eat, Pray, Move: A Pilot Cluster Randomized Controlled Trial of a Multilevel Church-Based Intervention to Address Obesity Among African Americans and Latinos. Am J Health Promot. 2019;33(4):586–96. <https://doi.org/10.1177/0890117118813333>.

10. Heath GW, Bilderback J. Grow Healthy Together: Effects of Policy and Environmental Interventions on Physical Activity Among Urban Children and Youth. J Phys Act Health. 2019;16(2):172–6. <https://doi.org/10.1123/jpah.2018-0026>.

11. Herens M, Bakker EJ, van Ophem J, Wagemakers A, Koelen M. Health-Related Quality of Life, Self-Efficacy and Enjoyment Keep the Socially Vulnerable Physically Active in Community-Based Physical Activity Programs: A Sequential Cohort Study. PLoS One. 2016;11(2):e0150025. <https://doi.org/10.1371/journal.pone.0150025>.

12. Jalaludin B, Maxwell M, Saddik B, Lobb E, Byun R, Gutierrez R, et al. A pre-and-post study of an urban renewal program in a socially disadvantaged neighbourhood in Sydney, Australia. BMC Public Health. 2012;12:521. <https://doi.org/10.1186/1471-2458-12-521>.

13. Ruijsbroek A, Wong A, den Hertog F, Droomers M, van den Brink C, Kunst AE, et al. Do inhabitants profit from integrating a public health focus in urban renewal programmes? A Dutch case study. PLoS One. 2022;17(6):e0270367. <https://doi.org/10.1371/journal.pone.0270367>.

14. Hunter RF, Cleland C, Cleary A, Droomers M, Wheeler BW, Sinnett D, et al. Environmental, health, wellbeing, social and equity effects of urban green space interventions: A meta-narrative evidence synthesis. Environ Int. 2019;130:104923. <https://doi.org/10.1016/j.envint.2019.104923>.

15. Kramer D, Droomers M, Jongeneel-Grimen B, Wingen M, Stronks K, Kunst AE. The impact of area-based initiatives on physical activity trends in deprived areas; a quasi-experimental evaluation of the Dutch District Approach. INTERNATIONAL JOURNAL OF BEHAVIORAL NUTRITION AND PHYSICAL ACTIVITY. 2014;11:36. <https://doi.org/10.1186/1479-5868-11-36>.

16. Adams EJ, Cavill N. Engaging communities in changing the environment to promote transport-related walking: Evaluation of route use in the 'Fitter for Walking' project. Journal of Transport and Health. 2015;2(4):580–94. <https://doi.org/10.1016/j.jth.2015.09.002>.

17. Hosford K, Firth C, Brauer M, Winters M. The effects of road pricing on transportation and health equity: A scoping review. Transp Rev. 2021;41:766–87. <https://doi.org/10.1080/01441647.2021.1898488>.

18. Hansmann KJ, Grabow M, McAndrews C. Health equity and active transportation: A scoping review of active transportation interventions and their impacts on health equity. Journal of Transport and Health. 2022;25:101346. <https://doi.org/10.1016/j.jth.2022.101346>.

19. Cook T, O’Brien S, Jackson K, Findley D, Searcy S. Behavioral Effects of Completing a Critical Link in the American Tobacco Trail. Transportation Research Record: Journal of the Transportation Research Board. 2016;2598:19–26. <https://doi.org/10.3141/2598-03>.

20. Goodman A, Cheshire J. PP70 Inequalities in the London bicycle sharing system revisited: impacts of extending the scheme to poorer areas but then doubling prices. Journal of Transport Geography. 2014;41. <https://doi.org/10.1016/j.jtrangeo.2014.04.004>.

21. Smith M, Hosking J, Woodward A, Witten K, MacMillan A, Field A, et al. Systematic literature review of built environment effects on physical activity and active transport - an update and new findings on health equity. Int J Behav Nutr Phys Act. 2017;14(1):158. <https://doi.org/10.1186/s12966-017-0613-9>.

22. Iroz-Elardo N, Schoner J, Fox EH, Brookes A, Frank LD. Active travel and social justice: Addressing disparities and promoting health equity through a novel approach to Regional Transportation Planning. Soc Sci Med. 2020;261:113211. <https://doi.org/10.1016/j.socscimed.2020.113211>.

23. Martin A, Morciano M, Suhrcke M. Determinants of bicycle commuting and the effect of bicycle infrastructure investment in London: Evidence from UK census microdata. Econ Hum Biol. 2021;41:100945. <https://doi.org/10.1016/j.ehb.2020.100945>.

24. Brownson RC, Housemann RA, Brown DR, Jackson-Thompson J, King AC, Malone BR, et al. Promoting physical activity in rural communities: walking trail access, use, and effects. Am J Prev Med. 2000;18(3):235–41. <https://doi.org/10.1016/s0749-3797(99)00165-8>.

25. Olstad DL, Ancilotto R, Teychenne M, Minaker LM, Taber DR, Raine KD, et al. Can targeted policies reduce obesity and improve obesity-related behaviours in socioeconomically disadvantaged populations? A systematic review. Obes Rev. 2017;18(7):791–807. <https://doi.org/10.1111/obr.12546>.

26. Dulin-Keita A, Clay O, Whittaker S, Hannon L, Adams IK, Rogers M, et al. The influence of HOPE VI neighborhood revitalization on neighborhood-based physical activity: A mixed-methods approach. Soc Sci Med. 2015;139:90–9. <https://doi.org/10.1016/j.socscimed.2015.06.002>.

27. Kodali HP, Wyka KE, Costa SA, Evenson KR, Thorpe LE, Huang TT. Association of Park Renovation With Park Use in New York City. JAMA Netw Open. 2024;7(4):e241429. <https://doi.org/10.1001/jamanetworkopen.2024.1429>.

28. Lopes F, Figueiredo L, Gil J, Trigueiro E. Evaluating the impact of social housing policies: Measuring accessibility changes when individuals move to social housing projects. Environment and Planning B: Urban Analytics and City Science. 2023. <https://doi.org/10.1177/23998083231218774>.

29. Zenk SN, Pugach O, Ragonese-Barnes M, Odoms-Young A, Powell LM, Slater SJ. Did Playground Renovations Equitably Benefit Neighborhoods in Chicago? J Urban Health. 2021;98(2):248–58. <https://doi.org/10.1007/s11524-020-00472-4>.

30. Barbosa Filho VC, Bandeira ADS, Minatto G, Linard JG, Silva JAD, Costa RMD, et al. Effect of a Multicomponent Intervention on Lifestyle Factors among Brazilian Adolescents from Low Human Development Index Areas: A Cluster-Randomized Controlled Trial. Int J Environ Res Public Health. 2019;16(2). <https://doi.org/10.3390/ijerph16020267>.

31. Carson RL, Castelli DM, Pulling Kuhn AC, Moore JB, Beets MW, Beighle A, et al. Impact of trained champions of comprehensive school physical activity programs on school physical activity offerings, youth physical activity and sedentary behaviors. Prev Med. 2014;69 Suppl 1:S12–9. <https://doi.org/10.1016/j.ypmed.2014.08.025>.

32. Mendoza JA, Levinger DD, Johnston BD. Pilot evaluation of a walking school bus program in a low-income, urban community. BMC Public Health. 2009;9:122. <https://doi.org/10.1186/1471-2458-9-122>.

33. Nathan N, Wolfenden L, Williams CM, Yoong SL, Lecathelinais C, Bell AC, et al. Adoption of obesity prevention policies and practices by Australian primary schools: 2006 to 2013. Health Educ Res. 2015;30(2):262–71. <https://doi.org/10.1093/her/cyu068>.

34. Sutherland RL, Campbell EM, Lubans DR, Morgan PJ, Nathan NK, Wolfenden L, et al. The Physical Activity 4 Everyone Cluster Randomized Trial: 2-Year Outcomes of a School Physical Activity Intervention Among Adolescents. Am J Prev Med. 2016;51(2):195–205. <https://doi.org/10.1016/j.amepre.2016.02.020>.

35. Vander Ploeg KA, McGavock J, Maximova K, Veugelers PJ. School-based health promotion and physical activity during and after school hours. Pediatrics. 2014;133(2):e371–8. <https://doi.org/10.1542/peds.2013-2383>.

36. Andrade ACS, Mingoti SA, Fernandes AP, Andrade RG, Friche AAL, Xavier CC, et al. Neighborhood-based physical activity differences: Evaluation of the effect of health promotion program. PLoS One. 2018;13(2):e0192115. <https://doi.org/10.1371/journal.pone.0192115>.

37. Candio PA-OX, Meads D, Hill AJ, Bojke L. Cost-effectiveness of a proportionate universal offer of free exercise: Leeds Let's Get Active. (1741-3850 (Electronic)).

38. Higgerson J, Halliday E, Ortiz-Nunez A, Barr B. The impact of free access to swimming pools on children’s participation in swimming. A comparative regression discontinuity study. Journal of Public Health. 2018;41(2):214–21. <https://doi.org/10.1093/pubmed/fdy079>.

39. Hoekman R, Breedveld K, Kraaykamp G. Providing for the rich? The effect of public investments in sport on sport (club) participation of vulnerable youth and adults. European Journal for Sport and Society. 2017;14(4):327–47. <https://doi.org/10.1080/16138171.2017.1421510>.

40. Rabiee F, Robbins A, Khan M. Gym for Free: The short-term impact of an innovative public health policy on the health and wellbeing of residents in a deprived constituency in Birmingham, UK. Health Education Journal. 2015;74(6):691–704. <https://doi.org/10.1177/0017896914553957>.

41. Reilly K, Bauman A, Reece L, Lecathelinais C, Sutherland R, Wolfenden L. Evaluation of a voucher scheme to increase child physical activity in participants of a school physical activity trial in the Hunter region of Australia. BMC Public Health. 2021;21(1):570. <https://doi.org/10.1186/s12889-021-10588-0>.

42. Taylor P, Panagouleas T, Kung SP. Access to English public sector sports facilities by disadvantaged groups and the effect of financial objectives. Managing Leisure. 2011;16(2):128–41. <https://doi.org/10.1080/13606719.2011.559091>.

43. Virmasalo I, Hasanen E, Pyykönen J, Nurmi M, Simula M, Salmikangas AK, et al. Closed due to COVID-19: effects of indoor sports restrictions on suburban adults’ physical activity behaviours. International Journal of Sport Policy and Politics. 2023;15(2):249–69. <https://doi.org/10.1080/19406940.2023.2178479>.

44. Williams O. Identifying adverse effects of area-based health policy: An ethnographic study of a deprived neighbourhood in England. Health Place. 2017;45:85–91. <https://doi.org/10.1016/j.healthplace.2017.02.011>.

45. Thomas M, Phongsavan P, McGill B, O'Hara B, Bauman A. A review of the impact of physical activity mass media campaigns on low compared to high socioeconomic groups. Health education research. 2018;33. <https://doi.org/10.1093/her/cyy032>.

46. Pena-Y-Lillo M, Lee CJ. A Communication Inequalities Approach to Disparities in Physical Activities: The Case of the VERB Campaign. JOURNAL OF HEALTH COMMUNICATION. 2019;24(2):111–20. <https://doi.org/10.1080/10810730.2019.1583699>.

47. Tomioka M, Braun Kl Fau - Compton M, Compton M Fau - Tanoue L, Tanoue L. Adapting Stanford's Chronic Disease Self-Management Program to Hawaii's multicultural population. (1758-5341 (Electronic)).

48. Esquivel M, Nigg CR, Fialkowski MK, Braun KL, Li F, Novotny R. Head Start Wellness Policy Intervention in Hawaii: A Project of the Children's Healthy Living Program. Child Obes. 2016;12(1):26–32. <https://doi.org/10.1089/chi.2015.0071>.

49. Tomayko EJ, Prince RJ, Hoiting J, Braun A, LaRowe TL, Adams AK. Evaluation of a multi-year policy-focused intervention to increase physical activity and related behaviors in lower-resourced early care and education settings: Active Early 2.0. Prev Med Rep. 2017;8:93–100. <https://doi.org/10.1016/j.pmedr.2017.08.008>.

50. Yoong SL, Finch M, Nathan N, Wiggers J, Lecathelinais C, Jones J, et al. A longitudinal study assessing childcare services' adoption of obesity prevention policies and practices. J Paediatr Child Health. 2016;52(7):765–70. <https://doi.org/10.1111/jpc.13252>.

51. Vahid Shahidi F, Sod-Erdene O, Ramraj C, Hildebrand V, Siddiqi A. Government social assistance programmes are failing to protect the health of low-income populations: evidence from the USA and Canada (2003-2014). J Epidemiol Community Health. 2019;73(3):198–205. <https://doi.org/10.1136/jech-2018-211351>.
