# Supplementary material for: The impact of public policy on socioeconomic equity in physical activity: a systematic review
Source: Int J Behav Nutr Phys Act. 2026 Feb 4;23:20. doi: 10.1186/s12966-026-01880-6 (PMC12964968; doi:10.1186/s12966-026-01880-6)
Supplement: Supplementary file 7 — Additional file 7. Adapted PROSPERO protocol. [file 12966_2026_1880_MOESM7_ESM.docx]

Additional file 7: Adapted PROSPERO protocol

| **PROSPERO Item** | **Amendment** | **Reasons** |
| --- | --- | --- |
| Review title and basic details | The aim of the review has been revised to: 'This review aims to assess the current evidence on the effects of public policies on equity in PA’.  Review type updated from rapid review to systematic review | To gain a comprehensive understanding of how policies impact inequities in PA, we included not only those that promote equity or have a positive impact, but also those that increase inequities, have no effect, or show mixed results.  We made amendments to update the review to a systematic review, which included the addition of a Risk of Bias assessment. |
| Eligibility criteria: Context | The inclusion criteria were refined to focus specifically on studies examining *socioeconomic* inequities. Eligible studies had to include either a socioeconomically disadvantaged subgroup or an entire disadvantaged population, defined by factors such as income, education, occupation, or area-level disadvantage. | To ensure a focused and meaningful analysis, we refined our scope and, in line with other reviews on equity effects, chose to concentrate on *socioeconomic* inequities [1-3]. |
| Data collection process: Data extraction | The items for data extraction have been reduced to details related to policy, measure(s) of inequity, PA-related outcome measure(s), overall main finding(s) (if differential effects by SES were available; otherwise, this was not applicable), and finding(s) related to equity. | Given the relatively large number of included studies, we streamlined the process to improve efficiency while ensuring accuracy. |
| Data collection process:  Risk of Bias (quality) assessment | We added Risk of Bias assessment | Update to systematic review |

**References**

1. Olstad DL, Teychenne M, Minaker LM, Taber DR, Raine KD, Nykiforuk CI, et al. Can policy ameliorate socioeconomic inequities in obesity and obesity-related behaviours? A systematic review of the impact of universal policies on adults and children. Obes Rev. 2016;17(12):1198–217. <https://doi.org/10.1111/obr.12457>.

2. Sassi F, Belloni A, Mirelman AJ, Suhrcke M, Thomas A, Salti N, et al. Equity impacts of price policies to promote healthy behaviours. The Lancet. 2018;391(10134):2059–70. <https://doi.org/10.1016/S0140-6736(18)30531-2>.

3. Brown T, Platt S, Amos A. Equity impact of population-level interventions and policies to reduce smoking in adults: a systematic review. Drug Alcohol Depend. 2014;138:7–16. <https://doi.org/10.1016/j.drugalcdep.2014.03.001>.
